# New NSAID Conjugates as Potent and Selective COX-2 Inhibitors: Synthesis, Molecular Modeling and Biological Investigation

**DOI:** 10.3390/molecules28041945

**Published:** 2023-02-17

**Authors:** Riham M. Bokhtia, Siva S. Panda, Adel S. Girgis, Nermin Samir, Mona F. Said, Anwar Abdelnaser, Soad Nasr, Mohamed S. Bekheit, Abdelhameed S. Dawood, Horrick Sharma, Margaret Wade, Swapnil K. Sharma, Amany M. Ghanim

**Affiliations:** 1 Department of Pharmaceutical Organic Chemistry, Faculty of Pharmacy, Zagazig University, Zagazig 44519, Egypt; 2 Department of Chemistry & Physics, Augusta University, Augusta, GA 30912, USA; 3 Department of Pesticide Chemistry, National Research Centre, Dokki, Giza 12622, Egypt; 4 Department of Pharmaceutical Chemistry, Faculty of Pharmacy, Ain Shams University, Cairo 11566, Egypt; 5 Department of Pharmaceutical Chemistry, Faculty of Pharmacy, Cairo University, Cairo 11562, Egypt; 6 Institute of Global Health and Human Ecology, School of Sciences and Engineering, The American University in Cairo (AUC), Cairo 11835, Egypt; 7 College of Pharmacy, Southwestern Oklahoma State University, Weatherford, OK 73096, USA; 8 Department of Computer Science and Engineering, University of California, Merced, CA 95343, USA

**Keywords:** ibuprofen, indomethacin, anti-inflammatory, analgesic, COX, molecular modeling

## Abstract

New sets of ibuprofen and indomethacin conjugates comprising triazolyl heterocycle were synthesized via click chemistry, adopting an optimized protocol through the molecular hybridization approach affording the targeted agents in good yields. The new non-steroidal anti-inflammatory drug (NSAID) conjugates were designed and synthesized and could be considered as potential drug candidates for the treatment of pain and inflammation. The anti-inflammatory properties were investigated for all the synthesized conjugates. Among 14 synthesized conjugates, four (**5a**, **5b**, **5d**, and **5e**) were found to have significant anti-inflammatory properties potency 117.6%, 116.5%, 93.8%, and 109.1% in comparison to reference drugs ibuprofen (97.2%) and indomethacin (100%) in the rat paw edema carrageenan test without any ulcerogenic liability. The suppression effect of cytokines IL-6, TNF-α, and iNOS in addition to NO in the LPS-induced RAW264.7 cells supports the promising anti-inflammatory properties observed in the ibuprofen conjugates. In addition, several conjugates showed promising peripheral and central analgesic activity. The selectivity index (SI) of compound **5a** (23.096) indicates the significant efficacy and selectivity for COX-2 over COX-1. Molecular modeling (docking and QSAR) studies described the observed biological properties.

## 1. Introduction

Cyclooxygenase (COX) is an enzyme that produces prostaglandins, prostacyclins, and thromboxanes—substances called prostanoids that are responsible for the inflammatory response [1]. COX is known as a rate-limiting enzyme because it serves as the major pathway or key for the formation of these prostanoids. However, COX also plays a vital role in normal cellular processes. Three isoforms (COX-1, COX-2, and COX-3) of COX have been identified [2,3]. Both COX-1 and COX-2 are responsible for important physiological processes and are involved in the pathological process of cancer, pain and inflammation. COX-1 is essential for the prostaglandin-mediated functions of the gastrointestinal and cardiovascular systems. Under normal conditions, COX-2 is present at a low level. However, COX-2 is expressed in the response to pro-inflammatory and pathogenic stimuli. In addition, COX-2 plays a critical role, not only in inflammation [1,4] but also in various pathologies that include cancer [5,6], neurodegenerative diseases [7], and multidrug resistance [8].

**Figure 1:**
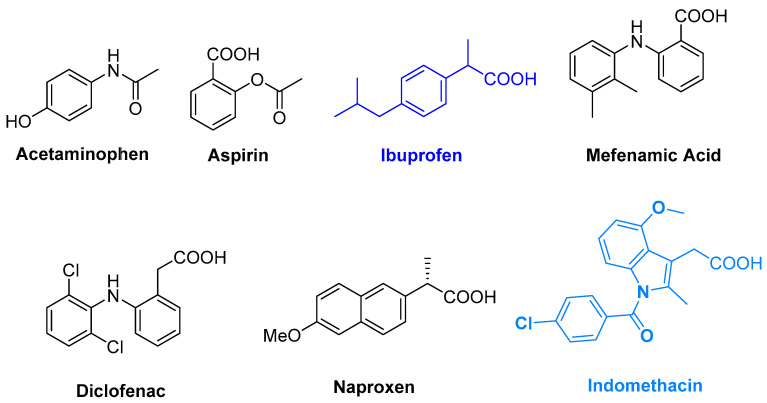
Clinically approved NSAIDs.

Since 1897, NSAIDs are in practice as the first-line drugs for pain and inflammation. The non-selective inhibition nature of traditional NSAIDs (Figure 1) against the cyclooxygenase isoenzymes (COX-1 and COX-2), restricts their uses because of many associated disadvantages like gastrointestinal and ulcerogenic side effects [9].

The development of new drug candidates with selective inhibitory effects against COX-2 over COX-1 is a challenging scientific subject because isoforms possess similar cellular expression, locations, and more than 60% sequence homology [10]. The molecular hybridization approach is one of the most powerful and attractive rational drug design strategies used for the development of new drug candidates. Moreover, the known COX-2 inhibitor drugs (e.g., celecoxib and rofecoxib) are serious drawbacks, especially for the cardiovascular system [11].

Ibuprofen (Ibu) is considered one of the safest and extensively used over-the-counter analgesic drugs. This is attributed to the approved efficiencies in musculoskeletal disorders, osteoarthritis, and rheumatoid arthritis. Indomethacin (Indo) is also a potent prescription NSAID. However, both Ibu and Indo are associated with several adverse effects in chronic use and have restricted use for the individual having other health complications [12,13].

**Figure 2:**
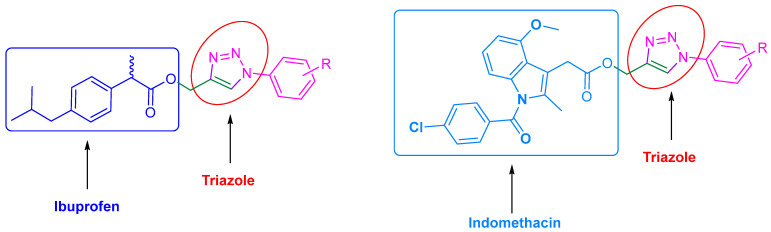
The targeted designed molecules: NSAID conjugates.

Including our group, several other research groups have been engaged in the development of potential NSAID conjugates [14,15,16,17,18,19]. Our recent attempts are to design and synthesize ibuprofen and indomethacin conjugates with potential anti-inflammatory and analgesic properties with minimal adverse effects. The ‘click’ chemistry approach is employed through the reaction of various azides with the alkyne moiety of the propargyl-containing ibuprofen and indomethacin (Figure 2). We considered ibuprofen and indomethacin as the scaffolds. The triazolyl heterocycle was considered due to its well-known diversified biological properties and importance in drug development [20,21,22,23,24,25,26,27]. The triazole ring framework is often considered under the bioisosterism approach because of its ability to interact non-covalently with the biomolecular targets and improve the therapeutic significance of the designed molecules. The targeted and well-characterized synthesized conjugates were screened for their anti-inflammatory and analgesic properties. Moreover, molecular modeling studies were also considered for the biological observations.

## 2. Results and Discussion

### 2.1. Chemistry

**Scheme 1:**
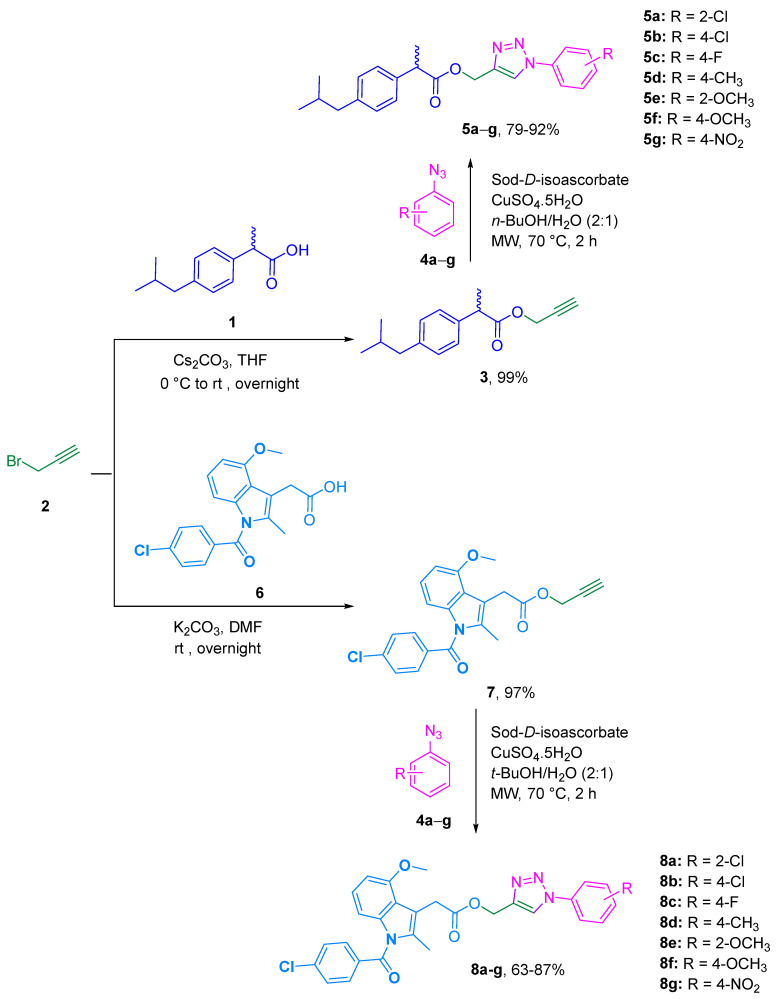
Synthesis of designed Ibu conjugates **5a**–**g** and Indo conjugates **8a**–**g**.

The synthetic pathway employed for the ibuprofen-containing triazolyl heterocycle **5** is depicted in Scheme 1. The alkyne component accessible for the click chemistry was developed through treating Ibu with propargyl bromide in presence of cesium carbonate (Cs_2_CO_3_) in THF (at 0 °C to room temp. incubated overnight). Further, the alkyne was treated with aromatic azides **4** (previously reported [27]) adopting our modified click chemistry technique [28] in presence of CuSO_4_·5H_2_O and sodium d-isoascorbate in *n*-butanol–water mixture under microwave irradiation for 2 h at 70 °C to obtain the desired conjugates **5a**–**g** in good yields (Scheme 1).

We followed a similar protocol to synthesize the indomethacin conjugates **8** from compound **7** using click chemistry (Scheme 1). However, the reaction yields are lower than the ibuprofen conjugates. We tried the reaction at different temperatures and reaction times, but we found microwave reaction gave a cleaner reaction with a better yield than conventional heating.

### 2.2. Biological Studies

#### 2.2.1. Anti-Inflammatory Properties

The well-established carrageenan-induced rat paw edema technique was adopted to determine the anti-inflammatory property of the synthesized conjugates [14,15,16]. Enhanced anti-inflammatory properties were revealed by some of the prepared conjugates with better potency than their precursors. However, no anti-inflammatory efficacy was observed for compounds **7**, **8b**, and **8f.**

**Figure 3:**
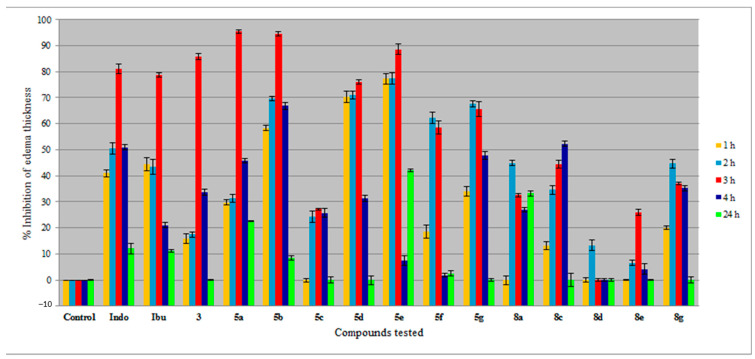
% Inhibition of edema thickness by the tested compounds.

SAR (structure–activity relationship) through the observed anti-inflammatory data (Table 1, Figure 3) optimizes a few items controlling the biological properties. Generally, conjugation of triazolyl heterocycle with ibuprofen scaffold affords better anti-inflammatory active agents than that of indomethacin (compound **5c** is an exception). Some conjugates with enhanced anti-inflammatory properties were observed relative to their parent drug [compounds **5a**, **5b**, and **5e** show % potency = 117.6, 116.5, 109.1, respectively, compared with % potency of ibuprofen (parent precursor) = 97.2].

For the ibuprofen–triazole conjugates, the chloro-substituted phenyl triazoles are of higher anti-inflammatory properties than those of methyl/methoxy substituted phenyls, as shown in compounds **5a**/**5b**/**5d**/**5e/5f**. Additionally, the ortho-substituted phenyl analogs are of better anti-inflammatory properties than the para-substituted conjugates as shown in pairs **5a**/**5b** and **5e**/**5f**. The last correlation was also noticed for indomethacin-triazole conjugates as exhibited in pairs **8a**/**8b** and **8e**/**8f**.

Compounds **5a** and **5e** that show high acute anti-inflammatory properties also reveal considerable potency after 24h relative to their standard drug (% inhibition of edema after 24 h = 22.5, 42.1, 11.2 for **5a**, **5e**, and ibuprofen, respectively). Although compound **8a** reveals mild acute anti-inflammatory activity, the enhanced property after 24 h was noticed relative to its reference standard (% inhibition of edema after 24 h = 33.3, 12.0 for **8a** and indomethacin, respectively).

#### 2.2.2. Analgesic Properties

##### Peripheral Analgesic

In vivo acetic acid-induced abdominal writhing assay in mice was undertaken for the peripheral analgesic testing of the prepared conjugates (10 mg/kg “animal body weight” indomethacin mol equivalent) [14,15]. Table 2 summarizes the observed results. It has been noticed that all the synthesized ibuprofen–triazole conjugates reveal peripheral analgesic properties with higher potencies (% potency = 88.2–121.9) than their parent drug (% potency of ibuprofen = 81.5). Additionally, few of the synthesized indomethacin–triazole conjugates are with enhanced biological properties (% potency of **8e**, **8g** = 112.5, 134.1, respectively) relative to their precursor (% potency of indomethacin = 100).

SAR can be assigned based on the revealed observations. Ibuprofen–triazole conjugates containing halogenated phenyl, are more effective agents than those with methyl- or methoxyphenyl analogs as in compounds **5b**/**5c**/**5d**/**5f** and **5a**/**5e**. Conjugate with chlorophenyl substituent is more potent than that of fluorophenyl ring as exhibited in pairs **5b**/**5c** (% potency = 121.9, 116.0, respectively). Additionally, the *p*-substituted phenyl conjugates are of higher analgesic properties than those of *ortho*-substituted phenyl analogs as shown in pairs **5a**/**5b** (% potency = 116.0, 121.9, respectively) and **5e**/**5f** (% potency = 88.2, 111.4, respectively).

Contrary, the *ortho*-substituted phenyl indomethacin–triazole conjugates show higher peripheral analgesic properties than those of the *p*-substituted phenyl conjugates as shown in pairs **8a**/**8b** and **8e**/**8f** (% potency = 68.7, 64.5 and 112.5, 93.0, respectively). Additionally, the methyl- and methoxyphenyl-containing indomethacin–triazole analogs are of better efficacies than the halogenated phenyl-containing conjugates as viewed in compounds **8d**/**8f**/**8c**/**8b** and **8e**/**8a**.

##### Central Analgesic

Central analgesic properties of the synthesized agents were undertaken by the hot plate assay in mice (10 mg/kg “animal body weight” indomethacin mol equivalent) [14,15]. It is noted that from the obtained results (Table 3), compound **8f** is the most effective agent synthesized with higher potency than its precursor (potency = 117.7, 100 for **8f** and indomethacin, respectively). Compound **5a** also reveals central analgesic potency comparable to its parent drug (potency = 96.5, 96.1 for **5a** and ibuprofen, respectively). It has also been noticed that, although compound **5c** reveals high analgesic properties at the first-time interval (1.8 folds % protection relative to its parent drug “ibuprofen at 30 min.”), the bio-properties drastically reduced by time (% protection = 18.0, 54.3 for **5c** and ibuprofen, respectively, at 120 min.). A similar observation was shown by conjugate **5f** (% protection = 90.1, 0.2; 61.7, 54.3 for **5f** and ibuprofen, respectively, at 30, 120 min. time intervals). Meanwhile, compound **8f** (the most potent agent synthesized) exhibits almost stable bio-observations throughout all the experimental time intervals in a similar profile to its parent drug (% protection = 66.3, 92.4, 68.4, 66.5; 87.1, 80.9, 57.9, 56.5 for **8f** and indomethacin at 30, 60, 90, 120 min., respectively).

SAR can be identified due to biological observations. For the ibuprofen–triazole conjugates, the halogenated phenyl-containing conjugates show higher central analgesic properties relative to the methyl- or methoxyphenyl containing compounds as shown in compounds **5b**/**5c**/**5d**/**5f** and **5a**/**5e**. The *ortho*-substituted phenyl containing triazoles show more potent biological properties than the *para*-substituted phenyl analogs as exhibited in pairs **5a**/**5b** (potency = 96.5, 48.0, respectively) and **5e**/**5f** (potency = 49.6, 0.4, respectively). Contrary, the methyl-, methoxyphenyl containing indomethacin–triazole conjugates show higher bio-properties than the fluoro-, chlorophenyl-containing conjugates “**8a** is an exception” [as shown in compounds **8d**/**8f** compared with **8b**/**8c** (potency = 86.0, 117.7, 17.2, 85.1, respectively)].

#### 2.2.3. Ulcerogenic Liability

The most promising anti-inflammatory active agents (**3**, **5a**, **5b**, **5d**, **5e**) were tested for ulcerogenic liability in mice [14,15]. Table 4 shows that none of the synthesized potential conjugates (**5a**, **5b**, **5d**, **5e**) show ulcers or erosions to the tested animal gastric which indicates their safe applicability for oral administration.

#### 2.2.4. Toxicological Bioassay

The most promising anti-inflammatory agents (**3**, **5a**, **5b**, **5d**, and **5e**) were tested for their toxicological effect in mice [14,15]. Five times the anti-inflammatory dose was orally administrated. No toxic symptoms or mortality were revealed by any of the tested compounds.

#### 2.2.5. Inhibitory Properties of COX-1 and COX-2

The inhibitory properties of the promising anti-inflammatory active agents (**3**, **5a**, **5b**, **5d**, and **5e**) against COX-1 and COX-2 were undertaken by the standard techniques obeying the manufacturer’s instructions [29]. It is noticed from the results observed (Table 5), that all the synthesized agents are with enhanced selectivity index [SI = IC_50 (COX-1)_/IC_50 (COX-2)_] towards COX-2 relative to COX-1. Conjugate **5a** is superior with a high SI = 23.096. Conjugate **5b** also has a promising SI value (SI = 9.619). It has also been noticed that the SI values are consistent with the anti-inflammatory % potency of most of the tested conjugates (SI = 2.262, 23.096, 9.619, 2.158 corresponding to % potency = 105.8, 117.6, 116.5, 93.8 for conjugates **3**, **5a**, **5b** and **5d**). Meanwhile, conjugate **5e** is with higher potency against COX-1 relative to its precursor supporting the anti-inflammatory properties observed (IC_50_ against COX-1 = 5.417, 13.16 μM corresponding to % potency = 109.1, 97.2, for **5e** and Ibu, respectively). Moreover, it is with a mild SI value (COX-1/COX-2) in similar behavior to its precursor (SI = 0.387, 0.106 for **5e** and Ibu, respectively).

#### 2.2.6. Evaluation of NO Production of LPS-Induced RAW264.7

**Figure 4:**
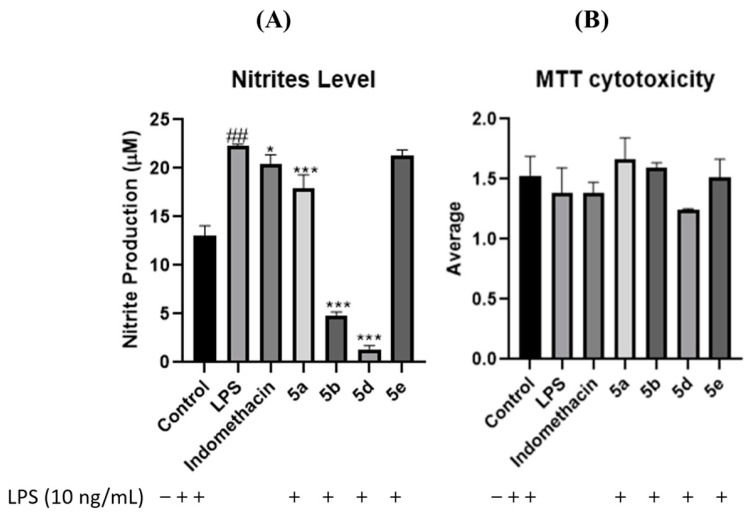
Evaluation of NO production and cell viability in LPS-stimulated RAW264.7 macrophages of Ibuprofen conjugates. (**A**) A concentration of 40 μg/mL of ibuprofen conjugates (**5a**, **5b**, **5d**, and **5e**) and indomethacin (positive control) was used for the treatment of LPS-stimulated RAW264.7 macrophages. The Griess method was used to evaluate the nitrite content in cell supernatants. Significant differences indicated in the graphs are all in comparison to LPS-stimulated cells only. (**B**) A concentration of 40 μg/mL of ibuprofen conjugates and indomethacin (positive control) was used for the treatment of LPS-stimulated RAW264.7 macrophages. Cytotoxicity of the macrophages was evaluated using an MTT assay. No significant cytotoxicity was observed for LPS-stimulated cells only (designated as ##)., Ibuprofen conjugates and indomethacin (positive control) in comparison to control. Values are mean ± SD (*n* = 3). * *p* < 0.05, *** *p* < 0.001.

The anti-inflammatory responses of ibuprofen conjugates (**5a**, **5b**, **5d**, **5e**) and indomethacin (reference standard) were assessed on LPS- stimulated RAW264.7 cells. The nitrite production in the supernatant in the culture medium was measured using Greiss reaction [30]. Based on the nitrite standard curve, it was found that the baseline of NO production in RAW 264.7 cells is 12.8 μM. More importantly, when RAW 264.7 cells were stimulated with LPS, NO production was significantly increased to 22 μM compared to the control (*** *p* < 0.001). Indomethacin (positive control) significantly decreased LPS-stimulated NO production to 18.8 ± 2.2 μM (* *p* < 0.05). However, the ibuprofen conjugates, **5a**, **5b**, and **5d**, significantly reduced the LPS-stimulated NO production to 17.7 μM ± 3.5, 4.88 ± 1 μM, and 1.45 ± 1.3 μM, respectively, compared to LPS-stimulated cells (*** *p* < 0.001, *** *p* < 0.001, *** *p* < 0.001, respectively). These results demonstrate that these ibuprofen conjugates **5a**, **5b**, and **5d** are superior to the positive control (indomethacin) in reducing NO production in LPS-stimulated RAW264.7 cells and highlighting the potential anti-inflammatory effect. Lastly, the ibuprofen conjugate **5e** showed an insignificant decrease (*p* > 0.05) in NO production in comparison to LPS-stimulated cells (Figure 4).

#### 2.2.7. Evaluation of mRNA Levels of Inflammatory Cytokines in LPS-Induced RAW264.7 Cells

**Figure 5:**
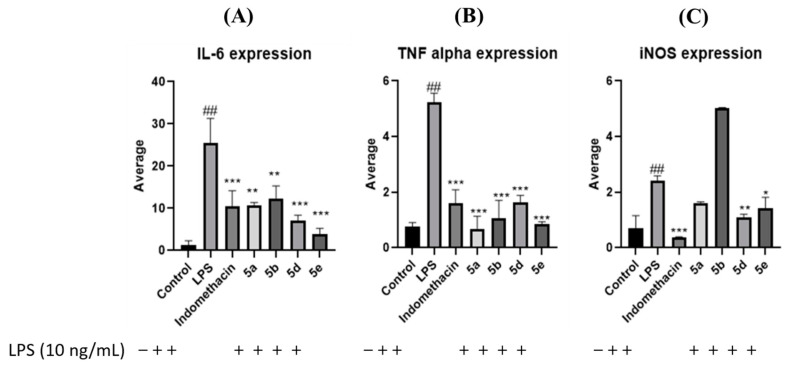
Expression levels of IL-6 (**A**), TNF-α (**B**), and iNOS mRNA (**C**) in LPS-stimulated RAW264.7 macrophages. A concentration of 40 μg/mL of indomethacin (positive control), **5a**, **5b**, **5d**, and **5e** was used for the treatment of LPS-stimulated RAW264.7 macrophages (10 ng/mL). The RT-qPCR was used to measure mRNA levels of IL-6, TNF-α, and iNOS using the comparative method (2^−ΔΔCT^). Significant differences indicated in the graphs are all in comparison to LPS-stimulated cells only (designated as ##). Values are means ± S.D. (*n* = 3). * *p* < 0.05, ** *p* < 0.001, *** *p* < 0.001.

Based on the previous results showing that the ibuprofen conjugates **5a**, **5b**, **5d**, and **5e** were able to decrease NO production in LPS-stimulated RAW 264.7 cells, we sought to determine the effect of these conjugates on the pro-inflammatory mRNA markers (IL-6, TNF-α, and iNOS). For this purpose, the mRNA levels of IL-6, TNF-α, and iNOS were measured using real-time qPCR (RT-qPCR). Our findings demonstrated that LPS resulted in a significant increase in the mRNA levels of IL-6, TNF-α, and iNOS (*** *p* < 0.001, *** *p* < 0.001, ** *p* < 0.01, respectively, in comparison to the control). However, treatment with the ibuprofen conjugates significantly decreased mRNA levels of IL-6, TNF-α, and iNOS in LPS-stimulated RAW264.7 cells (Figure 5). For IL-6 gene expression, a reduction in its levels was detected in LPS-stimulated RAW264.7 cells for the ibuprofen conjugates: 61.3, 42, 66.2 and 82% reduction for **5a**, **5b**, **5d** and **5e** (** *p* < 0.01, ** *p* < 0.01, *** *p* < 0.001, *** *p* < 0.001, respectively, in comparison to LPS-stimulated cells only). As for the positive control indomethacin, a 50% reduction in mRNA levels was observed. For TNF-α gene expression, a reduction in its levels was also detected in LPS-stimulated RAW264.7 cells for the ibuprofen conjugates: 88, 82, 72, 86% reduction for **5a**, **5b**, **5d**, and **5e** (*** *p* < 0.001, *** *p* < 0.001, *** *p* < 0.001, *** *p* < 0.001, respectively, in comparison to LPS-stimulated cells only). As for the positive control indomethacin, a 74% reduction in mRNA levels was observed. For iNOS gene expression, also a reduction in its levels was detected in LPS-stimulated RAW264.7 cells for the ibuprofen conjugates except for **5b**. A 34, 59, and 42% reduction in mRNA levels was observed for **5a**, **5d**, and **5e** (*p* > 0.05, ** *p* < 0.01, * *p* < 0.05, respectively, in comparison to LPS-stimulated cells only). As for the positive control indomethacin, an 85% reduction in mRNA levels was observed. Table S1 indicates the mRNA sequences used for the primers in RT-qPCR.

### 2.3. Molecular Modeling Studies

#### 2.3.1. Docking Studies

Molecular modeling is one of the computational techniques used intensively in medicinal chemistry. Among the compounds tested, compound **5a** is highly selective for COX-2, with a SI of 23.096 (Table 5). Compound **5e** is more potent in inhibiting COX-1 and is about three folds less active in inhibiting COX-2. To understand the underlying molecular basis of interaction leading to the selectivity of compounds, we carried out docking studies using the Glide program of the Schrodinger software, v2020-1 [31]. Glide extra precision (XP) mode, which uses a more sophisticated scoring system, is used for docking [32]. COX-2 crystal structure co-crystalized with selective inhibitor SC-558, PDB accession number 6COX, and COX-1 structure crystalized with COX-1 selective drug flurbiprofen (PDB entry: 3N8W) was used for docking simulations [2,32,33,34]. Glide poses were first validated by docking the native ligands of the 6COX and 3N8W structure in the receptor active site. Superimposition of the docked poses with their respective bioactive ligand conformations (Supplementary Materials Figure S1) yields a low root mean square deviation (RMSD) of 0.088 Å and 0.080 Å, respectively. It suggests that Glide XP docking can reproduce the ligand conformation in the COX crystal structures accurately.

**Figure 6:**
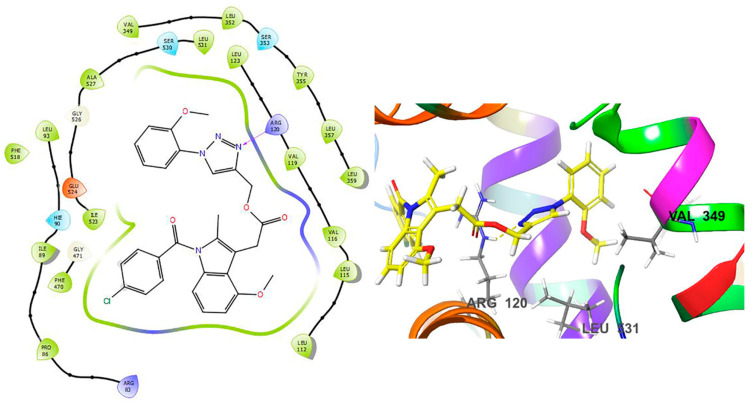
Binding interactions and docked pose of compound **5e** in the COX-1 crystal structure, PDB: 3N8W. Critical interactions with Arg120, Leu531, and Val349 are shown.

The binding mode of compound **5e** in the COX-1 active site (PDB 3N8W) reveals that the triazole ring lies perpendicular to the plane of the 2-OCH_3_ phenyl ring (Figure 6). In this orientation, the triazole ring makes a critical H-bonding interaction with Arg120, which is located at the entrance of the cyclooxygenase active site. Arachidonic acid (AA), the cyclooxygenase substrate, makes a salt bridge interaction with Arg120 of COX-1 with its carboxylic group [35]. Indeed, many non-selective NSAIDs, including the aryl acetic acid class of compounds, possess a carboxylic acid to mimic the interaction of AA with Arg120 [36]. The 2-methoxy substituent on the phenyl ring in **5e** fits into a hydrophobic groove near the enzyme active site. This 2-methoxy substituent may impart COX-1 inhibitory activity by interacting with residues Leu531 and Val349. Val349 in COX-1 helps position the substrate AA in the active site and confers catalytic activity to the enzyme leading to maximum PGG2 production [35]. In the drug indomethacin, the 2-methyl group on the indole ring interacts with Val349. Further, the 2ʹ-des methyl analog of indomethacin results in a complete loss of COX-1 activity and possesses only a very weak potency at COX-2 [37].

**Figure 7:**
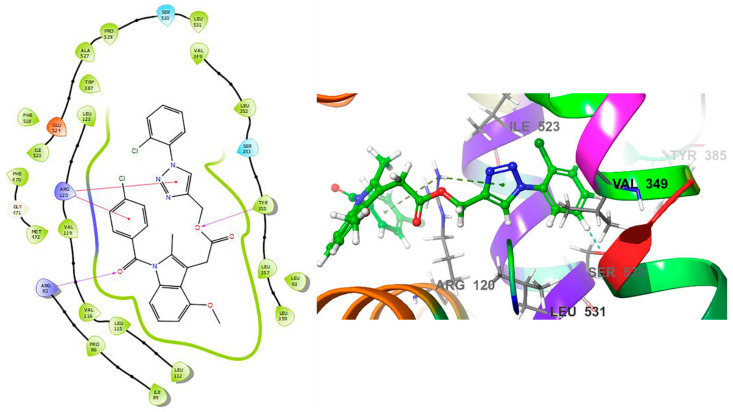
Binding interactions and docked pose of compound **5a** in the COX-1 crystal structure, PDB: 3N8W. There is a loss of interactions with Leu531 and Val349, whereas interaction with Ser530 is observed. The choro group is oriented on the other side of the binding pocket and interacts with Ile523.

The binding pose of **5a** (Figure 7). was analyzed to understand its weak COX-1 inhibitory activity. The triazole ring got flipped and is essentially coplanar with the 2-chloro phenyl ring. The chloro substituent makes vdW contact with Ile523. The drop of COX-1 potency in **5a** could be attributed to the loss of key interactions with Leu531 or Val 349. H-bonding interactions with Arg120 were also not observed, although a cation-pi interaction between Arg120 and the triazole ring could occur. The experimental data for **5a** and **5e** also agree with the observed Glide XP scores of −5.9 kcal/mol and −5.5 kcal/mol, respectively. Since docking scores are not a reliable indicator of binding free energies, we carried out MM-GBSA binding free energy (ΔG_bind_) calculations [38] on the docked poses of **5a** and **5e**. We observed a more significant difference in the binding free energy estimations (−87 kcal/mol for **5e** and −68 kcal/mol for **5a**), which correlated well with the observed COX-1 IC_50_s. The XP docking scores of **5b** and **5d** in structure 3N8W did not correlate with observed experimental data; however, their MM-GBSA-ΔG binding scores of −81.98 kcal/mol and −75.91 kcal/mol aligned well with observed potency. Overall, the COX-1 MM-GBSA-ΔG binding scores of all compounds **5a**, **5b**, **5d**, and **5e** correlated with observed potency values (Table 5).

**Figure 8:**
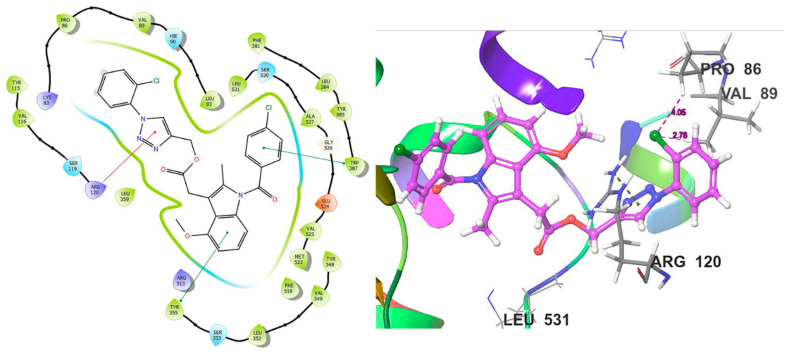
Binding interactions and docked pose of compound **5a** in the COX-2 crystal structure, PDB: 6COX. The choro group interacts with Pro86 and Val89, conferring **5a** COX-2 selectivity.

For COX-2, the substrate AA does not make a salt bridge with Arg120 but makes an H-bonding interaction with Tyr385 and Ser530. COX-2 has a larger side pocket formed by substituting His513 (in COX-1) with Arg 513 and mutation of Ile434 and 523 (in COX-1) to smaller valine residues [39]. In the COX-2 structure, the Leu531 is more flexible, oriented differently, and may not be critical for substrate binding or COX activity. In contrast, Leu531 mutations in COX-1 may lead to more than 90% loss of maximal cyclooxygenase activity [35]. To understand the greater COX-2 potency of **5a** and the relative loss of activity of **5e**, we docked these two compounds in the COX-2 crystal structure (PDB ID: 6COX). Interestingly, the Glide XP could dock only **5a** (Figure 8) but did not give any pose for compound **5e**. The docking simulations were carried out using default conditions, including Coulomb–van der Waals energy cut-off of 0 kcal/mol for pose filtering. To accept higher energy poses of **5e**, we relaxed the threshold to incorporate poses with the combined Coulomb and van der Waals interaction energy of 2 kcal/mol. However, the Glide XP again could not retrieve any pose for the compound, suggesting that **5e** may not optimally fit into the COX-2 active site. Switching the Glide XP mode to a less accurate Glide standard precision (SP) mode did dock **5e**; however, the SP pose was not considered for analysis. The MM-GBSA ΔG_bind_ calculations on the XP pose of compound **5a** gave a binding free energy of −69 kcal/mol, which was marginally better than the binding free energy of **5a** (68 kcal/mol) in the COX-1 active site. The triazole ring of **5a** in the COX-2 structure is oriented out of the plane (Figure 8) and interacts with Arg120 via cation-pi interactions. The presence of the 2-chloro substituent resulted in COX-2 selectivity, probably by interacting with Pro86 and Val89 through hydrophobic and vdW interactions. Val89 is a residue of the membrane binding domain of COX and is shown to confer greater COX-2 inhibitory potency [40]. We then docked compounds **5b** and **5d** in the 6COX active site. In agreement with the SAR we observed better docking (XP) and MMGBSA scores (−2.681, −59.66 kcal/mol) for **5d** over **5b** (1.145, −39.94 kcal/mol). MMGBSA binding free energy of **5a**, **5b**, and **5d** also aligned with the observed COX-2 experimental data (Table 5).

**Figure 9:**
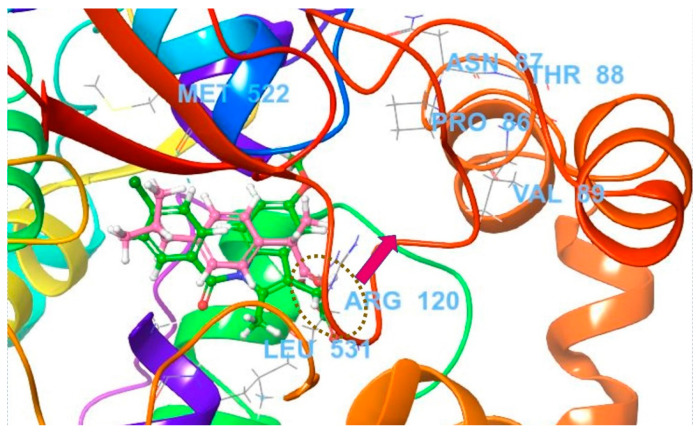
Overlay of docked (XP) poses of indomethacin (colored green) and ibuprofen (colored pink) in the COX-2 crystal structure, PDB: 6COX. The carboxylic acid moiety of indomethacin was used as a seed group for conjugation. Enhanced COX-2 potency was achieved by targeting Pro86 and Val89.

Compared to Indomethacin and Ibuprofen, our compound **5e** is more potent and selective for COX-2. Figure 9 shows an overlay of the docked poses of Indomethacin and Ibuprofen in the COX2 crystal structure, 6COX. The carboxylic acid group interacted with Arg120, but no contacts with Pro 86 and Val89 were observed. The binding mode indicates that the selectivity and potency for COX-2 could be achieved by appending groups that can target the loop residues, including Pro86, Asn87, Thr88, and Val89. We used the carboxylic acid of indomethacin as the seed group to which a phenyl-substituted triazole ring was conjugated. The position of the substituent on the phenyl ring proved critical to achieving selectivity and potency for COX-2, as demonstrated by the 2-chloro substituent in **5a**.

#### 2.3.2. D-QSAR Studies

QSAR can utilize the physicochemical parameters (descriptors) to express mathematically the biological properties. It is usable to rationalize the bio-properties exhibited. Prediction of new hits/leads based on a pre-assigned model and identification of parameters essential for bio-properties optimization are benefits of the QSAR technique [41,42].

#### 2.3.3. Anti-Inflammatory QSAR Model

CODESSA-Pro software was considered for the current QSAR studies [15]. A robust three-descriptor QSAR model (*R*^2^ = 0.979, *R*^2^cvOO = 0.951, *R*^2^cvMO = 0.963) describes the anti-inflammatory observations of the tested compounds (Supplementary Materials Tables S2–S4, Figure S2).

The charge-related-descriptor H-donors PSA (*t* = −5.545) negatively participated in the QSAR model with a coefficient value −0.0149. Therefore, a compound with a high mathematical descriptor value estimates low biological property, as exhibited in pairs **5c** and **5e** (descriptor value = 11.928, 1.909, corresponding to estimated anti-inflammatory property = 26.3, 89.7, respectively). The partial positively charged surface area is determined by Equation (1) [43].

(1) $$PPSA1\;\sum_{A}S_{A}\;\;A\in \left\{\delta_{A}>0\right\}$$

where, $S_{A}$ is the positively charged solvent-accessible atomic surface area.

Weighted PNSA is also a charge-related descriptor (*t* = −13.16). The low descriptor value of **5d** relative to that of **5c** explains its potent estimated anti-inflammatory property (descriptor value = 121.181, 148.1222, corresponding to estimated property = 84.8, 26.3, respectively) due to its negative coefficient sign (coefficient = −0.002) in the QSAR model. The surface-weighted charged partial negative charged surface area *WNSA*-1 is determined by Equation (2) [43].

(2) $$WNSA-1=\frac{PNSA1.TMSA}{1000}$$

*PNSA1* stands for the partial negatively charged molecular surface area, while *TMSA* stands for the total molecular surface area.

Relative negative-charged surface area is also a charge-related descriptor with a coefficient value −0.060. This explains the enhanced predicted anti-inflammatory property of **5b** relative to **5c** (descriptor value = 0.1223, 9.46504, corresponding to estimated property = 85.5, 26.3, respectively). The relative negative charge can be determined by Equation (3) [43].

(3) $$RNCG=\frac{\delta_{max}^{-}}{\sum_{A}^{}\delta_{A}}\;A\in \left\{\delta_{A}>0\right\}$$

where, $\delta_{max}^{-}$ stands for the maximum atomic negative charge in the molecule, while $\delta_{A}$ stands for the negative atomic charge in the molecule.

The statistical parameters (*F* = 123.9, *s*^2^ = 0.001) and the correlation of the observed and predicted anti-inflammatory properties of the tested compounds preserving their potencies among each other support the goodness of the QSAR model.

#### 2.3.4. Peripheral Analgesic QSAR Model

Statistically robust validated three-descriptor QSAR model expressed the observed analgesic properties (peripheral) of the synthesized analogs (Supplementary Materials Tables S5–S7, Figure S3). The semi-empirical descriptor average nucleophilic reactivity index for atom N possesses a coefficient value −0.981123 in the QSAR model determining 1/(property “% inhibition/protection”). The high descriptor value determines low potent analog as shown in compounds **5c** and **8c** (descriptor values = 0.00721, 0.00365 corresponding to the estimated % protection/inhibition = 86.0 and 42.5, respectively). Fukui atomic nucleophilic reactivity index can be calculated by Equation (4) [43].

(4) $$N_{A}=\sum_{i\in A}C_{iHOMO}^{2}/\left(1-\varepsilon_{HOMO}\right)$$

where $\varepsilon_{HOMO}$ and $C_{iHOMO}$ stand for the highest occupied molecular energy and coefficients, respectively.

Maximum atomic state energy for atom H is also a semi-empirical descriptor with a negative coefficient sign in the attained QSAR model. This explains the low estimated analgesic properties of compound **8c** relative to **8e** (descriptor values = 7.7989, 7.8328 corresponding to the estimated % protection/inhibition = 42.5 and 87.5, respectively). The electron–electron repulsion and attraction energies for a given atomic species can be determined by Equations (5) and (6), respectively [43].

(5) $$E_{ee}\left(A\right)=\sum_{B\neq A}\sum_{\mu ,\nu \in A}\sum_{\lambda ,\sigma \in B}P_{\mu \nu }P_{\lambda \sigma }\langle \mu \nu |\lambda \sigma \rangle$$

(6) $$E_{ne}\left(AB\right)=\sum_{B}\sum_{\mu ,\nu \in A}P_{\mu \nu }\langle \mu |\frac{Z_{B}}{R_{iB}}|\nu \rangle$$

where *A* and *B* are two different atoms, $P_{\mu \nu }$, $P_{\lambda \sigma }$ are the density matrix elements over atomic basis {μνλσ}, and 〈μν|λσ〉 is the electron repulsion integral on atomic basis {μνλσ}. $P_{\mu \nu }$ is the density matrix elements over atomic basis {μν}. $Z_{B}$ is the charge of atomic nucleus Β. $R_{iB}$ is the distance between the electron and atomic nucleus Β. $\langle \mu |\frac{Z_{B}}{R_{iB}}|\nu \rangle$ is the electron–nucleus attraction integral on atomic basis {μν}.

Again, the maximum electrophilic reactivity index for atom C is a semi-empirical descriptor with a negative coefficient value (−1.28007) in the 2D-QSAR model attained. This is why compound **5c** reveals potent estimated biological observation relative to **8a** (descriptor values = 0.02583, 0.02041 corresponding to the estimated % protection/inhibition = 86.0 and 47.4, respectively). Fukui’s atomic electrophilic reactivity index is determined by Equation (7) [43].

(7) $$E_{A}=\sum_{j\in A}C_{jLUMO}^{2}/\left(\varepsilon_{LUMO}+10\right)$$

where $\varepsilon_{LUMO}$ and $C_{jLUMO}$ are the lowest unoccupied molecular orbital energy and coefficients, respectively.

The comparative values of observed and predicted analgesic properties support the goodness of QSAR model attained (Supplementary Materials Table S6).

#### 2.3.5. Central Analgesic QSAR Model

CODESSA-Pro was utilized for optimizing the statistically robust three-descriptor QSAR model (*R*^2^ = 0.967, *R*^2^cvOO = 0.941, *R*^2^cvMO = 0.949) utilizing the homogeneous (non-diverse) bio-active conjugates revealing variable biological properties (observed % protection = 0.2–66.5) (Supplementary Materials Tables S8–S10, Figure S4). Maximum resonance energy for bond H–C is a semi-empirical descriptor positively participated in the QSAR model determining directly the % protection (property) of the tested agents with high coefficient value (coefficient = 402.343). The high descriptor value optimizes potent analgesic agents as shown in analogs **8f** and **5d** (descriptor value = 11.44, 11.3564 corresponding to predicted property = 68.4, −2.1, respectively). Resonance energy between two atoms can be calculated by Equation (8) [43].

(8) $$E_{R}AB\;\;\sum_{\mu \in A}\sum_{\nu \in B}P_{\mu \nu }\beta_{\mu \nu }$$

where *A* and *B* stand for two different atomic species. $P_{\mu \nu }$ and $\beta_{\mu \nu }$ stand for the density matrix elements and resonance integrals, respectively, over the atomic basis {μν}.

Fractional PNSA is a charge-related descriptor that also positively participated in the QSAR model with a high coefficient value (coefficient = 615.863). This explains the high estimated property of conjugate **8f** over **5g** (descriptor value = −0.0549, −0.08934 corresponding to predicted property = 68.4, 25.2, respectively). The fractional atomic charge for the weighted surface area (partially positive) can be calculated by Equation (9) [43].

(9) $$FPSA3=\frac{PPSA3}{TMSA}$$

Since PPSA3 and TMSA stand for the total charge (partially positive) weighted molecular surface area and the total molecular surface area, respectively.

The maximum 1-electron reactivity index for atom N is a semi-empirical descriptor that negatively participated in the QSAR model with the highest coefficient value among all the other descriptors (coefficient = −8834.15). So, the analog with a high descriptor value leads directly to low biologically active agents as revealed in compounds **5d** and **8f** (descriptor value = 0.00536, 0 with predicted property = −2.1, 68.4, respectively). Fukui atomic one-electron reactivity index can be calculated by Equation (10) [43].

(10) $$R_{A}=\sum_{i\in A}\sum_{j\in A}C_{iHOMO}C_{jLUMO}/\left(\varepsilon_{LUMO}-\varepsilon_{HOMO}\right)$$

where $C_{iHOMO}$ and $C_{jLUMO}$ stand for the highest occupied and the lowest unoccupied molecular oribital coefficients.

The predicted biological properties based on the QSAR model attained are comparable to the observed central analgesic properties (Supplementary Materials Table S9).

## 3. Experimental Section

### 3.1. Chemistry

Melting points were determined on a capillary point apparatus equipped with a digital thermometer and are uncorrected. NMR spectra were recorded in CDCl_3_ on a Bruker spectrometer operating at 500 MHz for ^1^H NMR (with TMS as an internal standard) and 125 MHz for ^13^C NMR. The microwave-assisted reaction was carried out with a single-mode cavity Discover Microwave Synthesizer (CEM Corporation, NC). The reaction mixtures were transferred into a 10 mL glass pressure microwave tube equipped with a magnetic stir bar. The tube was closed with a silicon septum and the reaction mixture was subjected to microwave irradiation (Discover mode; run time: 120 s; Power Max-cooling mode). High-resolution mass spectra were recorded with a TOF analyzer spectrometer by using electron spray mode.

### 3.2. Preparation of Prop-2-yn-1-yl 2-(4-isobutylphenyl) Propanoate (3)

To a solution of ibuprofen **1** (100 mg, 0.48 mmol) in THF (5 mL), cesium carbonate (316 mg, 0.97 mmol) and propargyl bromide **2** (0.10 mL, 0.97 mmol) were added. The reaction mixture was stirred starting at 0 °C, allowing the temperature to room temperature overnight and TLC monitored the progress of the reaction. After completion of the reaction, the solvent was evaporated under reduced pressure and the residue was treated with cold water then extracted with ethyl acetate and dried under vacuum to get the desired compound **3** in pure form. Colorless oil, yield: 99% (117 mg). IR: *ν*_max_/cm^−1^; 3046, 2954, 2869, 2200, 1739, 1512, 1198; ^1^H NMR δ: 7.18 (d, *J* = 8.1 Hz, 2H, Ar–H), 7.08 (d, *J* = 8.1 Hz, 2H, Ar–H), 4.69 (dd, *J* = 15.6, 2.5 Hz, 1H, CH_2_), 4.58 (dd, *J* = 15.6, 2.5 Hz, 1H, CH_2_), 3.72 (q, *J* = 7.2 Hz, 1H, CH), 2.44–2.41 (m, 3H, CH + CH_2_), 1.87–1.79 (m, 1H, CH), 1.49 (d, *J* = 7.2 Hz, 3H, CH_3_), 0.88 (d, *J* = 6.6 Hz, 6H, 2CH_3_); ^13^C NMR δ: 174.1, 140.9, 137.4, 129.6, 127.4, 77.9, 75.0, 52.4, 45.2, 45.1, 30.4, 22.6, 18.8; HRMS: *m*/*z* for C_16_H_20_O_2_ [M^+^] Calcd.: 244.1463, Found: 244.1460.

### 3.3. Preparation of Prop-2-yn-1-yl 2-(1-(4-chlorobenzoyl)-5-methoxy-2-methyl-1H-indol-3-yl) Acetate (7)

A round bottom flask (50 mL) containing a small stir bar was charged with a suspension of indomethacin **6** (500 mg, 1.40 mmol) in DMF (20 mL) along with anhydrous K_2_CO_3_ (386 mg, 2.79 mmol). The reaction mixture was stirred at room temperature for 30 min then propargyl bromide **2** (0.25 mL, 2.79 mmol) was added. The reaction mixture was stirred overnight, and TLC monitored the progress of the reaction. After completion of the reaction, the reaction mixture was poured into iced water and extracted with ethyl acetate (20 mL) three times. The combined organic layer was dried over sodium sulfate; then, the crude product was subjected to column chromatography to give pure compound **7**. Brown oil, yield: 97% (1.07 g). IR: *ν*_max_/cm^−1^; 3032, 2931, 2900, 2120, 1739, 1656, 1478, 1255, 835; ^1^H NMR δ: 7.63 (d, *J* = 8.4 Hz, 2H, Ar–H), 7.44 (d, *J* = 8.4 Hz, 2H, Ar–H), 6.94 (d, *J* = 2.5 Hz, 1H, Ar–H), 6.85 (d, *J* = 9.0 Hz, 1H, Ar–H), 6.65 (dd, *J* = 9.0, 2.4 Hz, 1H, Ar–H), 4.68 (d, *J* = 2.4 Hz, 2H, CH_2_), 3.81 (s, 3H, OCH_3_), 3.69 (s, 2H, CH_2_), 2.45 (s, 1H, CH), 2.36 (s, 3H, CH_3_); ^13^C NMR δ: 170.1, 168.4, 162.7, 156.2, 139.4, 136.2, 134.0, 131.3, 130.9, 130.6, 129.3, 115.1, 111.9, 101.3, 77.6, 75.3, 55.8, 52.6, 36.6, 31.6, 30.2, 13.5; HRMS: *m*/*z* for C_22_H_18_ClNO_4_ [M^+^] Calcd.: 395.0924, Found: 395.0931.

### 3.4. General Method for Preparation of 5a–g and 8a–g

A dried heavy-walled Pyrex tube containing a small stir bar was charged with a solution of the respective alkyne derivative (either **3** or **7**) (500 mg, 1.0 eq.) in *n*-BuOH/H_2_O or *t*-BuOH/H_2_O mixture (2:1, 3 mL) “in case of compounds **3** and **7**, respectively”. Sodium d-isoascorbate monohydrate (0.4 eq.) and copper sulfate pentahydrate (0.3 eq.) were added at room temperature and the corresponding aryl azide **4a**–**g** (1.2 eq.) was added. The reaction mixture was exposed to microwave irradiation (20 W) at 70 °C for 2 h and monitored by TLC. The mixture was allowed to cool down and then quenched with ice-cold water (15 mL). The product was extracted with ethyl acetate and the organic layer was washed with brine solution and dried over anhydrous sodium sulfate. The solvent was removed under reduced pressure and the targeted compounds **5a**–**g** and **8a**–**g** were isolated in good yields after purification using column chromatography (10% ethyl acetate/hexanes).

#### 3.4.1. *(1-(2-Chlorophenyl)-1H-1,2,3-triazol-4-yl)methyl 2-(4-isobutylphenyl)propanoate* (**5a**)

Yellow oil, yield: 79% (0.64 g). IR: *ν*_max_/cm^−1^; 3018, 2954, 2868, 1732, 1496, 1235, 1199, 759; ^1^H NMR δ: 7.80 (s, 1H, CH), 7.55–7.53 (m, 2H, Ar–H), 7.45–7.39 (m, 2H, Ar–H), 7.31 (d, *J* = 8.1 Hz, 2H, Ar–H), 7.04 (d, *J* = 8.1 Hz, 2H, Ar–H), 5.31 (s, 2H, CH_2_), 3.73 (q, *J* = 7.2 Hz, 1H, CH), 2.38 (d, *J* = 7.2 Hz, 2H, CH_2_), 1.81–1.75 (m, 1H, CH), 1.48 (d, *J* = 7.2 Hz, 3H, CH_3_), 0.84 (d, *J* = 6.6 Hz, 6H, 2CH_3_); ^13^C NMR δ: 178.6, 174.7, 143.1, 140.8, 137.5, 134.9, 131.0, 130.9, 128.8, 128.1, 127.9, 127.4, 127.3, 125.7, 58.0, 45.2, 30.3, 22.5, 18.5; HRMS: *m*/*z* for C_22_H_24_ClN_3_O_2_ [M^+^] Calcd.: 397.1557, Found: 397.1563.

#### 3.4.2. *(1-(4-Chlorophenyl)-1H-1,2,3-triazol-4-yl)methyl 2-(4-isobutylphenyl)propanoate* (**5b**)

Yellowish white solid, m.p. 92–94 °C, yield: 86% (0.70 g). IR: *ν*_max_/cm^−1^; 3046, 2972, 2882, 1734, 1503, 1231, 1197, 822; ^1^H NMR δ: 7.73 (s, 1H, CH), 7.60–7.57 (m, 2H, Ar–H), 7.47–7.45 (m, 2H, Ar–H), 7.17 (d, *J* = 8.0 Hz, 2H, Ar–H), 7.06 (d, *J* = 8.0 Hz, 2H, Ar–H), 5.28 (s, 2H, CH_2_), 3.72 (q, *J* = 7.1 Hz, 1H, CH), 2.40 (d, *J* = 7.2 Hz, 2H, CH_2_), 1.83–1.75 (m, 1H, CH), 1.48 (d, *J* = 7.2 Hz, 3H, CH_3_), 0.85 (d, *J* = 6.6 Hz, 6H, 2 CH_3_); ^13^C NMR δ: 174.8, 144.3, 141.0, 137.5, 135.5, 134.9, 130.1, 129.6, 127.4, 121.9, 121.6, 58.1, 45.2, 30.4, 22.6, 18.5; HRMS: *m*/*z* for C_22_H_24_ClN_3_O_2_ [M^+^] Calcd.: 397.1557, Found: 397.1559.

#### 3.4.3. *(1-(4-Fluorophenyl)-1H-1,2,3-triazol-4-yl)methyl 2-(4-isobutylphenyl)propanoate* (**5c**)

Yellowish white solid, m.p. 80–82 °C, yield: 85% (0.66 g). IR: *ν*_max_/cm^−1^; 3098, 2954, 1728, 1514, 1229, 1200, 1053; ^1^H NMR δ: 7.71 (s, 1H, CH), 7.63–7.59 (m, 2H, Ar–H), 7.18 (t, *J* = 8.3 Hz, 4H, Ar–H), 7.06 (d, *J* = 8.0 Hz, 2H, Ar–H), 5.28 (s, 2H, CH_2_), 3.72 (q, *J* = 7.1 Hz, 1H, CH), 2.40 (d, *J* = 7.2 Hz, 2H, CH_2_), 1.84–1.75 (m, 1H, CH), 1.48 (d, *J* = 7.2 Hz, 3H, CH_3_), 0.85 (d, *J* = 6.6 Hz, 6H, 2CH_3_); ^13^C NMR δ: 174.8, 163.7, 161.7, 144.2, 140.9, 137.5, 133.3, 129.6, 127.4, 122.8, 121.9, 117.0, 116.8, 58.1, 45.2, 30.4, 22.6, 18.5; HRMS: *m*/*z* for C_22_H_24_FN_3_O_2_ [M^+^] Calcd.: 381.1853, Found: 381.1859.

#### 3.4.4. *(1-(p-Tolyl)-1H-1,2,3-triazol-4-yl)methyl 2-(4-isobutylphenyl)propanoate* (**5d**)

Yellowish white solid, m.p. 84–86 °C, yield: 92% (0.71 g). IR: *ν*_max_/cm^−1^; 3016, 2973, 2926, 2868, 1735, 1520, 1232, 1198; ^1^H NMR δ: 7.73 (s, 1H, CH), 7.51 (d, *J* = 8.4 Hz, 2H, Ar–H), 7.27 (d, *J* = 8.2 Hz, 2H, Ar–H), 7.17 (d, *J* = 8.0 Hz, 2H, Ar–H), 7.06 (d, *J* = 8.1 Hz, 2H, Ar–H), 5.28 (s, 2H, CH_2_), 3.72 (q, *J* = 7.1 Hz, 1H, CH), 2.41 (s, 2H, CH_2_), 2.40 (s, 3H, CH_3_), 1.84–1.76 (m, 1H, CH), 1.48 (d, *J* = 7.2 Hz, 3H, CH_3_), 0.85 (d, *J* = 6.6 Hz, 6H, 2CH_3_); ^13^C NMR δ: 174.8, 143.8, 140.9, 139.2, 137.5, 134.8, 130.4, 129.5, 127.4, 121.7, 120.6, 58.2, 45.2, 30.3, 22.6, 21.3, 18.5; HRMS: *m*/*z* for C_23_H_27_N_3_O_2_ [M^+^] Calcd.: 377.2103, Found: 377.2110.

#### 3.4.5. *(1-(2-Methoxyphenyl)-1H-1,2,3-triazol-4-yl)methyl 2-(4-isobutylphenyl)propanoate* (**5e**)

Yellow oil, yield: 87% (0.62 g). IR: *ν*_max_/cm^−1^; 3048, 2954, 2868, 1732, 1602, 1506, 1254, 1198; ^1^H NMR δ: 8.00 (s, 1H, CH), 7.72 (dd, *J* = 7.9, 1.6 Hz, 1H, Ar–H), 7.40 (dt, *J* = 8.3, 1.6 Hz, 1H, Ar–H), 7.17 (d, *J* = 8.0 Hz, 2H, Ar–H), 7.09–7.04 (m, 4H, Ar–H), 5.33 (d, *J* = 12.8 Hz, 1H, CH_2_), 5.25 (d, *J* = 12.8 Hz, 1H, CH_2_), 3.84 (s, 3H, OCH_3_), 3.72 (t, *J* = 7.2 Hz, 1H, CH), 2.39 (d, *J* = 7.2 Hz, 2H, CH_2_), 1.83–1.75 (m, 1H, CH), 1.48 (d, *J* = 7.2 Hz, 3H, CH_3_), 0.85 (d, *J* = 6.6 Hz, 6H, 2 CH_3_); ^13^C NMR δ: 174.8, 151.3, 142.5, 140.8, 137.6, 130.4, 129.5, 127.4, 126.0, 125.7, 121.4, 112.4, 58.3, 56.1, 45.2, 30.4, 22.6, 18.7; HRMS: *m*/*z* for C_23_H_27_N_3_O_3_ [M^+^] Calcd.: 393.2052, Found: 393.2059.

#### 3.4.6. *(1-(4-Methoxyphenyl)-1H-1,2,3-triazol-4-yl)methyl 2-(4-isobutylphenyl)propanoate* (**5f**)

Colorless oil, yield: 85% (0.68 g), IR: *ν*_max_/cm^−1^; 3132, 2955, 2867, 2843, 1727, 1518, 1440; ^1^H NMR δ: 7.73 (s, 1H, CH), 7.55 (d, *J* = 9.0 Hz, 2H, Ar–H), 7.21 (d, *J* = 8.1 Hz, 2H, Ar–H), 7.08 (d, *J* = 8.1 Hz, 2H, Ar–H), 6.99 (d, *J* = 9.0 Hz, 2H, Ar–H), 5.30 (s, 2H, OCH_2_), 3.85 (s, 3H, OCH_3_), 3.75 (q, *J* = 7.2 Hz, 1H), 2.43 (d, *J* = 7.2 Hz, 2H, CH_2_), 1.87–1.78 (m, 1H, CH), 1.51 (d, *J* = 7.2 Hz, 3H, CH_3_), 0.88 (d, *J* = 6.6 Hz, 6H, 2 CH_3_); ^13^C NMR δ: 174.6, 159.9, 143.6, 140.7, 137.4, 130.3, 129.4, 127.2, 122.1, 121.7, 114.8, 56.0, 55.6, 45.0, 44.9, 30.2, 22.4, 18.3; HRMS: *m*/*z* for C_23_H_27_N_3_O_3_ [M]^+^ Calcd.: 393.2052. Found: 393.2055.

#### 3.4.7. *(1-(4-Nitrophenyl)-1H-1,2,3-triazol-4-yl)methyl 2-(4-isobutylphenyl)propanoate* (**5g**)

Yellow crystals, yield: 89% (0.74 g), m.p. 65 °C. IR: *ν*_max_/cm^−1^; 3139, 3098, 2954, 2866, 1740, 1598, 1517, 1371; ^1^H NMR δ: 8.43 (d, *J* = 9.1 Hz, 2H, Ar–H), 7.92 (d, *J* = 9.1 Hz, 2H, Ar–H), 7.88 (s, 1H, CH), 7.23 (d, *J* = 8.0 Hz, 2H, Ar–H), 7.11 (d, *J* = 8.0 Hz, 2H, Ar–H), 5.38–5.32 (m, 2H, OCH_2_), 3.78 (q, *J* = 7.1 Hz, 1H, CH), 2.46 (d, *J* = 7.2 Hz, 2H, CH_2_), 1.89–1.81 (m, 1H, CH), 1.54 (d, *J* = 7.1 Hz, 3H, CH_3_), 0.90 (d, *J* = 6.6 Hz, 6H, 2 CH_3_); ^13^C NMR δ: 174.6, 147.3, 144.8, 141.0, 140.8, 137.2, 129.4, 127.3, 125.5, 121.4, 120.5, 57.7, 45.0, 44.9, 30.2, 22.4, 18.2; HRMS: *m*/*z* for C_22_H_24_N_4_O_4_ [M]^+^ Calcd.: 408.1797. Found: 408.1801.

#### 3.4.8. *(1-(2-Chlorophenyl)-1H-1,2,3-triazol-4-yl)methyl 2-(1-(4-chlorobenzoyl)-5-methoxy-2-methyl-1H-indol-3-yl)acetate* (**8a**)

Yellow oil, yield: 65% (0.45 g). IR: *ν*_max_/cm^−1^; 3045, 2948, 2879, 1737, 1680, 1590, 1477, 1316, 1221, 729; ^1^H NMR δ: 7.92 (s, 1H, CH), 7.62 (d, *J* = 8.5 Hz, 2H, Ar–H), 7.54 (dd, *J* = 6.9, 2.4 Hz, 2H, Ar–H), 7.46–7.42 (m, 4H, Ar–H), 6.90 (d, *J* = 2.5 Hz, 1H, Ar–H), 6.85 (d, *J* = 9.0 Hz, 1H, Ar–H), 6.63 (dd, *J* = 9.0, 2.5 Hz, 1H, Ar–H), 5.34 (s, 2H, CH_2_), 3.76 (s, 3H, OCH_3_), 3.70 (s, 2H, CH_2_), 2.33 (s, 3H, CH_3_); ^13^C NMR δ: 170.9, 168.5, 156.3, 142.7, 139.5, 136.2, 134.8, 134.1, 131.4, 131.1, 131.0, 130.7, 129.3, 128.8, 128.1, 127.9, 126.1, 115.2, 112.4, 111.9, 101.4, 58.3, 55.9, 30.5, 13.6; HRMS: *m*/*z* for C_28_H_22_Cl_2_N_4_O_4_ [M^+^] Calcd.: 548.1018, Found: 548.1011.

#### 3.4.9. *(1-(4-Chlorophenyl)-1H-1,2,3-triazol-4-yl)methyl 2-(1-(4-chlorobenzoyl)-5-methoxy-2-methyl-1H-indol-3-yl)acetate* (**8b**)

Yellow oil, yield: 87% (0.59 g), IR: *ν*_max_/cm^−1^; 3152, 3112, 2924, 2851, 1735, 1677, 1309; ^1^H NMR δ: 7.85 (s, 1H, CH), 7.66 (d, *J* = 8.4 Hz, 2H, Ar–H), 7.62 (d, *J* = 8.8 Hz, 2H, Ar–H), 7.51 (d, *J* = 8.8 Hz, 2H, Ar–H), 7.49 (d, *J* = 8.4 Hz, 2H, Ar–H), 6.96 (d, *J* = 2.5 Hz, 1H, Ar–H), 6.91 (d, *J* = 9.0 Hz, 1H, Ar–H), 6.69 (dd, *J* = 9.0, 2.5 Hz, 1H, Ar–H), 5.36 (s, 2H, OCH_2_), 3.80 (s, 3H, OCH_3_), 3.75 (s, 2H, CH_2_), 2.39 (s, 3H, CH_3_); ^13^C NMR δ: 170.7, 168.3, 156.0, 143.8, 139.4, 136.1, 135.3, 134.7, 133.8, 131.2, 130.8, 130.5, 130.0, 129.2, 121.7, 115.0, 112.1, 111.6, 101.4, 58.1, 55.7, 30.3, 13.4; HRMS: *m*/*z* for C_28_H_22_Cl_2_N_4_O_4_ [M]^+^ Calcd.: 548.1018. Found: 548.1021.

#### 3.4.10. *(1-(4-Fluorophenyl)-1H-1,2,3-triazol-4-yl)methyl 2-(1-(4-chlorobenzoyl)-5-methoxy-2-methyl-1H-indol-3-yl)acetate* (**8c**)

White crystals, yield: 85% (0.57 g), m.p. 71 °C. IR: *ν*_max_/cm^−1^; 3093, 2925, 2853, 1731, 1688, 1525, 1477; ^1^H NMR δ: 7.84 (s, 1H, CH), 7.67–7.63 (m, 4H, Ar–H), 7.49 (d, *J* = 8.5 Hz, 2H, Ar–H), 7.25–7.22 (m, 2H, Ar–H), 6.96 (d, *J* = 2.5 Hz, 1H, Ar–H), 6.91 (d, *J* = 9.0 Hz, 1H, Ar–H), 6.69 (dd, *J* = 9.0, 2.5 Hz, 1H, Ar–H), 5.36 (s, 2H, CH_2_O), 3.79 (s, 3H, OCH_3_), 3.74 (s, 2H, CH_2_), 2.39 (s, 3H, CH_3_); ^13^C NMR δ: 170.7, 168.3, 162.5 (d, *J* = 249.4 Hz), 156.1, 143.6, 139.4, 136.1, 133.8, 133.1, 131.2, 130.8, 130.5, 129.2, 122.5 (d, *J* = 8.6 Hz), 122.0, 116.8 (d, *J* = 23.2 Hz), 115.0, 112.1, 111.6, 101.4, 58.2, 55.7, 30.3, 13.4; HRMS: *m*/*z* for C_28_H_22_ClFN_4_O_4_ [M]^+^ Calcd.: 532.1313. Found: 532.1321.

#### 3.4.11. *(1-(p-Tolyl)-1H-1,2,3-triazol-4-yl)methyl 2-(1-(4-chlorobenzoyl)-5-methoxy-2-methyl-1H-indol-3-yl)acetate* (**8d**)

Yellow solid, m.p. 130–132 °C, yield: 72% (0.48 g). IR: *ν*_max_/cm^−1^; 3002, 2954, 2918, 1725, 1673, 1608, 1519, 1332, 1216, 818; ^1^H NMR δ: 7.80 (s, 1H, CH), 7.61 (d, *J* = 8.5 Hz, 2H, Ar–H), 7.49 (d, *J* = 8.4 Hz, 2H, Ar–H), 7.42 (d, *J* = 8.5 Hz, 2H, Ar–H), 7.28 (d, *J* = 8.2 Hz, 2H, Ar–H), 6.92 (d, *J* = 2.5 Hz, 1H, Ar–H), 6.86 (d, *J* = 9.0 Hz, 1H, Ar–H), 6.64 (dd, *J* = 9.0, 2.5 Hz, 1H, Ar–H), 5.32 (s, 2H, CH_2_), 3.75 (s, 3H, OCH_3_), 3.70 (s, 2H, CH_2_), 2.40 (s, 3H, CH_3_), 2.34 (s, 3H, CH_3_); ^13^C NMR δ: 170.9, 168.5, 156.3, 143.5, 139.5, 139.3, 136.3, 134.7, 134.0, 131.4, 131.0, 130.7, 130.5, 129.3, 122.0, 120.6, 115.2, 112.4, 111.9, 101.5, 58.4, 55.9, 30.5, 21.3, 13.6; HRMS: *m*/*z* for C_29_H_25_ClN_4_O_4_ [M^+^] Calcd.: 528.1564, Found: 528.1571.

#### 3.4.12. *(1-(2-Methoxyphenyl)-1H-1,2,3-triazol-4-yl)methyl 2-(1-(4-chlorobenzoyl)-5-methoxy-2-methyl-1H-indol-3-yl)acetate* (**8e**)

Yellow paste, yield: 63% (0.43 g). IR: *ν*_max_/cm^−1^; 3061, 2987, 1729, 1681, 1589, 1532, 1311, 1217, 740; ^1^H NMR δ: 8.24 (s, 1H, CH), 8.07 (d, *J* = 8.8 Hz, 1H, Ar–H), 7.99 (dd, *J* = 8.8, 2.3 Hz, 1H, Ar–H), 7.93 (d, *J* = 2.2 Hz, 1H, Ar–H), 7.62–7.60 (m, 2H, Ar–H), 7.44–7.43 (m, 2H, Ar–H), 6.88–6.84 (m, 3H, Ar–H), 6.61 (dd, *J* = 9.0, 2.5 Hz, 1H, Ar–H), 5.33 (s, 2H, CH_2_), 3.97 (s, 3H, OCH_3_), 3.74 (s, 3H, OCH_3_), 3.69 (s, 2H, CH_2_), 2.33 (s, 3H, CH_3_); ^13^C NMR δ: 170.9, 168.5, 156.2, 150.8, 148.4, 142.9, 139.6, 136.2, 134.0, 131.4, 131.0, 130.9, 130.7, 129.3, 126.0, 125.4, 116.9, 115.1, 112.3, 111.9, 108.0, 101.5, 58.2, 57.0, 55.9, 30.4, 13.6; HRMS: *m*/*z* for C_29_H_25_ClN_4_O_5_ [M^+^] Calcd.: 544.1513, Found: 544.1511.

#### 3.4.13. *(1-(4-Methoxyphenyl)-1H-1,2,3-triazol-4-yl)methyl 2-(1-(4-chlorobenzoyl)-5-methoxy-2-methyl-1H-indol-3-yl)acetate* (**8f**)

Brown solid, m.p. 108–110 °C, yield: 67% (0.46 g). IR: *ν*_max_/cm^−1^; 3004, 2926, 1726, 1645, 1606, 1519, 1215, 831; ^1^H NMR δ: 7.77 (s, 1H, CH), 7.60 (d, *J* = 8.5 Hz, 2H, Ar–H), 7.51 (d, *J* = 9.0 Hz, 2H, Ar–H), 7.42 (d, *J* = 8.5 Hz, 2H, Ar–H), 6.97 (d, *J* = 9.0 Hz, 2H, Ar–H), 6.91 (d, *J* = 2.5 Hz, 1H, Ar–H), 6.86 (d, *J* = 9.0 Hz, 1H, Ar–H), 6.64 (dd, *J* = 9.0, 2.5 Hz, 1H, Ar–H), 5.31 (s, 2H, CH_2_), 3.84 (s, 3H, OCH_3_), 3.74 (s, 3H, OCH_3_), 3.69 (s, 2H, CH_2_), 2.33 (s, 3H, CH_3_); ^13^C NMR δ: 170.9, 168.5, 160.2, 156.2, 143.4, 139.5, 136.2, 134.0, 131.3, 131.0, 130.7, 130.4, 129.3, 122.3, 122.2, 115.2, 115.0, 112.4, 111.9, 101.5, 58.3, 55.8, 30.5, 13.6; HRMS: *m*/*z* for C_29_H_25_ClN_4_O_5_ [M^+^] Calcd.: 544.1513, Found: 544.1519.

#### 3.4.14. *(1-(4-Nitrophenyl)-1H-1,2,3-triazol-4-yl)methyl 2-(1-(4-chlorobenzoyl)-5-methoxy-2-methyl-1H-indol-3-yl)acetate* (**8g**)

Yellow crystals, yield 85% (0.60 g), m.p. 67 °C. IR: *ν*_max_/cm^−1^; 3093, 2925, 2853, 1731, 1688, 1596, 1525; ^1^H NMR δ: 8.42 (d, *J* = 9.1 Hz, 2H, ArH), 7.93 (s, 1H, Ar–H), 7.89 (d, *J* = 9.1 Hz, 2H, Ar–H), 7.67 (d, *J* = 8.5 Hz, 2H, Ar–H), 7.49 (d, *J* = 8.5 Hz, 2H, Ar–H), 6.97 (d, *J* = 2.5 Hz, 1H, Ar–H), 6.92 (d, *J* = 9.0 Hz, 1H, Ar–H), 6.70 (dd, *J* = 9.0, 2.5 Hz, 1H, Ar–H), 5.39 (s, 2H, CH_2_O), 3.80 (s, 3H, OCH_3_), 3.76 (s, 2H, CH_2_), 2.40 (s, 3H, CH_3_); ^13^C NMR δ: 170.7, 168.4, 156.0, 147.4, 144.5, 140.9, 139.5, 136.2, 133.7, 131.2, 130.9, 130.4, 129.2, 125.5, 121.6, 120.5, 115.0, 112.0, 111.6, 101.5, 58.0, 55.8, 30.3, 13.4; HRMS: *m*/*z* for C_28_H_22_ClN_5_O_6_ [M]^+^ Calcd.: 559.1258. Found: 559.1255.

### 3.5. Biological and Computational Studies

The RAW 264.7 macrophage cell line (ATCC TIB-71) was grown at 37 °C in a 5% CO_2_ humidified incubator. The media used for culturing as suggested by ATCC was high glucose DMEM supplemented with 10% heat inactivated fetal bovine serum (FBS) and 1% Pen-Strep (100 units/mL penicillin and 100 μg/mL streptomycin). Details of the experimental techniques utilized for biological and computational studies are mentioned in the Supplementary Materials file. All the biological procedures utilized obey the standards and were approved by the Research Ethics Committee, Faculty of Pharmacy, Cairo University, Egypt (number PC: 2989). All the experiments were performed in accordance with the relevant guidelines and regulations.

## 4. Conclusions

In summary, novel sets of ibuprofen and indomethacin-containing compounds (**5** and **8**) were designed and synthesized using a molecular hybridization approach. Compounds **5a**, **5b**, **5d**, and **5e** exhibited promising anti-inflammatory properties relative to their parent drugs ibuprofen and indomethacin. No ulcerogenic liability was shown by all the potent conjugates (**5a**, **5b**, **5d**, and **5e**) synthesized supporting their enhanced properties. Considerable selectivity towards COX-2 was noticed by the most promising anti-inflammatory agents synthesized through in vitro COX-1/COX-2 inhibitory testing compared to parent drugs, ibuprofen, and indomethacin. The suppression effect of LPS-induced production of NO, and cytokines IL-6, TNF-α, and iNOS in RAW264.7 cells support the promising anti-inflammatory properties observed in the ibuprofen conjugates. Molecular modeling explained the observed biological properties.

## Figures and Tables

**Table 1:** Anti-inflammatory properties of the synthesized conjugates.

Entry	Compd.	Mean Edema Thickness “mm” (% Inhibition of Edema ± Standard Error)					% Potency ^a^
		1 h	2 h	3 h	4 h	24 h	
1	Control	0.863 ± 0.02 (0.00 ± 0.01)	0.713 ± 0.03 (0.00 ± 0.02)	0.723 ± 0.02 (0.00 ± 0.01)	0.713 ± 0.01 (0.00 ± 0.03)	0.920 ± 0.07 (0.00 ± 0.04)	---
2	Indo	0.510 ± 0.03 ** (40.9 ± 1.2)	0.353 ± 0.05 ** (50.5 ± 2.1)	0.137 ± 0.01 ** (81.1 ± 1.8)	0.350 ± 0.04 ** (50.9 ± 1.1)	0.810 ± 0.03 ** (12.0 ± 2.1)	100
3	Ibu	0.480 ± 0.04 ** (44.4 ± 2.5)	0.403 ± 0.09 ** (43.5 ± 2.9)	0.153 ± 0.01 ** (78.8 ± 0.9)	0.563 ± 0.02 * (21.0 ± 1.1)	0.817 ± 0.07 * (11.2 ± 0.6)	97.2
4	**3**	0.727 ± 0.05 * (15.8 ± 1.9)	0.589 ± 0.01 * (17.4 ± 0.9)	0.103 ± 0.04 ** (85.8 ± 1.1)	0.473 ± 0.03 * (33.7 ± 1.2)	0.920 ± 0.02 * (0.00 ± 0.3)	105.8
5	**5a**	0.607 ± 0.04 * (29.7 ± 1.1)	0.490 ± 0.03 * (31.3 ± 0.8)	0.033 ± 0.01 ** (95.4 ± 0.7)	0.387 ± 0.06 ** (45.7 ± 1.4)	0.713 ± 0.02 ** (22.5 ± 0.9)	117.6
6	**5b**	0.360 ± 0.01 ** (58.3 ± 0.7)	0.217 ± 0.07 ** (69.6 ± 2.1)	0.040 ± 0.01 ** (94.5 ± 0.4)	0.237 ± 0.08 ** (66.8 ± 1.6)	0.843 ± 0.09 ** (8.4 ± 1.2)	116.5
7	**5c**	0.863 ± 0.08 * (0.00 ± 1.0)	0.540 ± 0.01 * (24.3 ± 1.4)	0.527 ± 0.03 * (27.1 ± 0.7)	0.530 ± 0.05 * (25.7 ± 0.9)	0.920 ± 0.01 * (0.00 ± 0.2)	33.4
8	**5d**	0.257 ± 0.08 ** (70.2 ± 2.2)	0.206 ± 0.04 ** (71.1 ± 1.5)	0.173 ± 0.01 ** (76.1 ± 0.8)	0.490 ± 0.07 ** (31.3 ± 1.1)	0.920 ± 0.09 * (0.00 ± 1.7)	93.8
9	**5e**	0.197 ± 0.02 ** (77.2 ± 1.9)	0.161 ± 0.08 ** (77.4 ± 2.2)	0.083 ± 0.02 ** (88.5 ± 2.0)	0.660 ± 0.09 * (7.4 ± 1.8)	0.533 ± 0.01 * (42.1 ± 0.4)	109.1
10	**5f**	0.703 ± 0.09 * (18.5 ± 2.5)	0.270 ± 0.05 * (62.1 ± 2.2)	0.300 ± 0.07 * (58.5 ± 2.4)	0.700 ± 0.10 * (1.8 ± 0.7)	0.897 ± 0.08 * (2.5 ± 0.9)	72.1
11	**5g**	0.570 ± 0.05 ** (34.0 ± 1.8)	0.230 ± 0.02 ** (67.7 ± 1.1)	0.249 ± 0.07 ** (65.6 ± 2.9)	0.373 ± 0.04 ** (47.7 ± 1.5)	0.920 ± 0.02 * (0.00 ± 0.5)	80.9
12	**8a**	0.863 ± 0.07 * (0.00 ± 1.5)	0.393 ± 0.08 * (44.9 ± 1.7)	0.488 ± 0.07 * (32.5 ± 1.5)	0.521 ± 0.03 * (26.9 ± 1.1)	0.614 ± 0.09 * (33.3 ± 2.4)	40.1
13	**8c**	0.750 ± 0.08 * (13.1 ± 1.7)	0.466 ± 0.02 * (34.6 ± 0.9)	0.403 ± 0.01 ** (44.3 ± 0.6)	0.340 ± 0.04 ** (52.3 ± 0.8)	0.920 ± 0.04 * (0.00 ± 1.0)	54.6
14	**8d**	0.863 ± 0.07 * (0.00 ± 0.8)	0.618 ± 0.09 * (13.3 ± 1.9)	0.723 ± 0.04 * (0.00 ± 0.5)	0.713 ± 0.03 * (0.00 ± 0.6)	0.920 ± 0.02 * (0.00 ± 0.4)	---
15	**8e**	0.863 ± 0.01 * (0.00 ± 0.2)	0.667 ± 0.05 * (6.5 ± 1.0)	0.536 ± 0.07 * (25.9 ± 1.3)	0.683 ± 0.08 * (4.2 ± 1.9)	0.920 ± 0.01 * (0.00 ± 0.3)	31.8
16	**8g**	0.690 ± 0.02 ** (20.0 ± 0.7)	0.394 ± 0.08 ** (44.7 ± 1.7)	0.456 ± 0.01 ** (36.9 ± 0.5)	0.462 ± 0.04 ** (35.2 ± 0.9)	0.920 ± 0.05 * (0.00 ± 1.2)	45.5

^a^ Potency is the % inhibition of the edema thickness of the synthesized agent relative to indomethacin at 3 h. Statistical analysis was conducted by one-way ANOVA (* *p* < 0.05, ** *p* < 0.001).

**Table 2:** Peripheral analgesic properties of the synthesized agents.

Entry	Compd.	Writing Reflex ± SE	% Inhibition/Protection	Potency ^a^
1	Control	40 ± 3.2	0	---
2	Indo	11.3 ± 1.6 **	71.8	100.0
3	Ibu	16.6 ± 0.7 **	58.5	81.5
4	**3**	11.3 ± 0.9 **	71.8	100.0
5	**5a**	6.7 ± 0.7 **	83.3	116.0
6	**5b**	5.0 ± 0.9 **	87.5	121.9
7	**5c**	6.7 ± 0.5 **	83.3	116.0
8	**5d**	7.0 ± 1.3 **	82.5	114.9
9	**5e**	14.7 ± 1.6 **	63.3	88.2
10	**5f**	8.0 ± 1.4 **	80.0	111.4
11	**5g**	14.0 ± 1.9 **	65.0	90.5
12	**7**	4.3 ± 0.5 **	89.3	124.4
13	**8a**	20.3 ± 1.8 *	49.3	68.7
14	**8b**	21.5 ± 1.5 *	46.3	64.5
15	**8c**	23.5 ± 1.4 *	41.3	57.5
16	**8d**	15.0 ± 1.6 *	62.5	87.0
17	**8e**	7.7 ± 1.3 **	80.8	112.5
18	**8f**	13.3 ± 1.9 **	66.8	93.0
19	**8g**	1.5 ± 0.4 **	96.3	134.1

^a^ Potency is the % inhibition/protection compared to indomethacin. * *p* < 0.05, ** *p* < 0.001.

**Table 3:** Central analgesic properties of the prepared agents.

Entry	Compd.	Latency Period ± SE “Standard Error”, Second (% Protection)				Potency ^a^
		After 30 min.	After 60 min.	After 90 min.	After 120 min.	
1	Control	6.68 ± 1.0 (0.0)	7.27 ± 1.4 (0.0)	8.91 ± 0.9 (0.0)	9.01 ± 1.1 (0.0)	---
2	Indo	12.50 ± 2.0 ** (87.1)	13.15 ± 1.6 ** (80.9)	14.07 ± 1.2 ** (57.9)	14.10 ± 1.9 ** (56.5)	100
3	Ibu	10.80 ± 0.8 ** (61.7)	11.00 ± 1.3 ** (51.3)	11.90 ± 1.5 ** (33.6)	13.90 ±2.2 ** (54.3)	96.1
4	**3**	10.37 ± 1.6 ** (55.2)	10.78 ± 2.1 ** (48.3)	10.62 ± 2.2 ** (19.2)	10.35 ± 1.7 ** (14.9)	26.4
5	**5a**	11.97 ± 2.5 ** (79.2)	12.47 ± 2.0 ** (71.5)	11.98 ± 2.6 ** (34.5)	13.92 ± 2.3 ** (54.5)	96.5
6	**5b**	12.30 ± 1.6 ** (84.1)	10.27 ± 1.9 ** (41.3)	11.14 ± 1.5 ** (25.0)	11.45 ± 2.0 ** (27.1)	48.0
7	**5c**	14.17 ± 2.6 ** (112.1)	15.0 ± 2.3 **(106.3)	14.91 ± 2.5 ** (67.3)	10.63 ± 1.8 ** (18.0)	31.9
8	**5d**	11.02 ± 1.4 ** (65.0)	9.86 ± 0.7 * (35.6)	10.69 ± 0.9 * (20.0)	9.12 ± 1.1 * (1.2)	2.1
9	**5e**	12.61 ± 2.1 ** (88.8)	10.53 ± 1.8 * (44.8)	12.12 ± 1.7 * (36.03)	11.53 ± 1.3 * (28.0)	49.6
10	**5f**	12.7 ± 0.9 * (90.1)	11.40 ± 1.5 * (56.8)	10.60 ± 1.9 * (19.0)	9.03 ± 1.6 * (0.2)	0.4
11	**5g**	12.5 ± 2.1 ** (87.1)	12.59 ± 1.5 ** (73.2)	10.60 ± 2.6 ** (19.0)	11.54 ± 2.2 ** (28.1)	49.7
12	**7**	6.68 ± 2.0 * (0.0)	9.54 ± 1.6 * (31.2)	11.18 ± 1.5 * (25.5)	10.78 ± 1.8 * (19.6)	34.7
13	**8a**	12.19 ± 1.9 ** (82.5)	13.51 ± 1.7 ** (85.8)	13.85 ± 1.4 ** (55.4)	13.63 ± 1.3 ** (51.3)	90.8
14	**8b**	6.84 ± 1.4 * (2.4)	8.31 ± 1.9 * (14.3)	9.91 ± 1.7 * (11.2)	9.88 ± 2.0 * (9.7)	17.2
15	**8c**	11.32 ± 0.8 * (69.5)	9.39 ± 1.1 * (29.2)	11.40 ± 1.6 * (27.9)	13.34 ± 1.8 * (48.1)	85.1
16	**8d**	13.21 ± 2.5 ** (97.8)	12.65 ± 2.0 ** (74.0)	13.37 ± 1.6 ** (50.1)	13.39 ± 1.2 ** (48.6)	86.0
17	**8e**	11.30 ± 1.1 ** (69.2)	13.23 ± 1.6 ** (82.0)	11.36 ± 2.5 ** (27.5)	11.61 ± 2.2 ** (28.9)	51.2
18	**8f**	11.11 ± 1.6 ** (66.3)	13.99 ± 1.9 ** (92.4)	15.00 ± 2.5 ** (68.4)	15.00 ± 2.6 ** (66.5)	117.7
19	**8g**	10.46 ± 2.4 ** (56.6)	10.57 ± 2.7 ** (45.4)	12.58 ± 2.0 ** (41.2)	9.25 ± 2.3 * (2.7)	4.8

^a^ Potency is that at 120 min. compared to indomethacin. * *p* < 0.05, ** *p* < 0.001.

**Table 4:** Ulcerogenic liability of the tested analogs.

Entry	Compd.	Number of Animals with Ulcer	% Incidence of Ulcer Divided by 10	Average of Ulcer Number	Average Severity of Ulcer	Ulcer Index
1	Control	0/6	0	0	0	0
2	Indo	6/6	10	2	1.67	13.67
3	Ibu	2/6	3.33	0.33	0.67	4.33
4	**3**	2/6	3.33	0.33	0.67	4.33
5	**5a**	0	0	0	0	0
6	**5b**	0	0	0	0	0
7	**5d**	0	0	0	0	0
8	**5e**	0	0	0	0	0

**Table 5:** COX-1 and COX-2 inhibitory properties of the tested compounds.

Entry	Compd.	IC_50_, μM ± SD		SI
		COX-1	COX-2	
1	Indo	0.354 ± 0.01 **	3.239 ± 0.09 **	0.109
2	IBU	13.16 ± 0.37 **	124.2 ± 3.53 *	0.106
3	**3**	19.45 ± 0.55 **	8.599 ± 0.24 **	2.262
4	**5a**	17.16 ± 0.41 **	0.743 ± 0.02 **	23.096
5	**5b**	118.8 ± 3.4 **	12.35 ± 0.35 **	9.619
6	**5d**	9.638 ± 0.27 **	4.467 ± 0.13 **	2.158
7	**5e**	5.417 ± 0.15 **	13.99 ± 0.40 **	0.387

* *p* < 0.05, ** *p* < 0.001.

## Data Availability

Not applicable.

## Supplementary Materials

The following supporting information can be downloaded at: https://www.mdpi.com/article/10.3390/molecules28041945/s1, Figure S1: (a) Overlay of the bioactive conformation of Flurbiprofen (green) with the Glide XP docked pose of the ligand in COX-1 crystal structure, PDB entry 3N8W; (b) Overlay of the bioactive conformation of SC-558 (green) with the Glide XP docked pose of the ligand in COX-2 crystal structure, PDB entry 6COX; Figure S2: QSAR plot representing the observed versus predicted log[% inhibition of edema thickness for the tested compounds at 10 mg/kg (rat body weight) indomethacin mol equivalent at 3 h effect]; Figure S3: QSAR plot representing the observed versus predicted 1/property “% inhibition of peripheral analgesic properties for the tested compounds at 10 mg/kg (rat body weight) indomethacin mol equivalent”; Figure S4: QSAR plot representing the observed versus predicted property “% protection for the central analgesic tested compounds at 10 mg/kg (rat body weight) indomethacin mol equivalent”; Table S1: mRNA sequences used for RT-qPCR; Table S2: Descriptors of the QSAR model for the tested anti-inflammatory active agents; Table S3: Observed and estimated anti-inflammatory properties for the tested compounds according to the BMLR-QSAR model; Table S4: Molecular descriptor values of the QSAR model for the tested compounds; Table S5: Descriptors of the QSAR model for the tested peripheral analgesic active agents; Table S6: Observed and estimated peripheral analgesic properties for the tested compounds according to the BMLR-QSAR model; Table S7: Molecular descriptor values of QSAR model for the peripheral analgesic active agents; Table S8: Descriptors of the QSAR model for the tested central analgesic active agents; Table S9: Observed and estimated central analgesic properties for the tested compounds according to the QSAR model; Table S10: Molecular descriptor values of the QSAR model for the central analgesic tested compounds.; ^1^HNMR spectra and ^13^C NMR spectra of **5a**–**g** and **8a**–**g**.

## Sample Availability

Samples of the compounds **5a**–**g** and **8a**–**g** are available from the authors.
