# Supplementary material for: New NSAID Conjugates as Potent and Selective COX-2 Inhibitors: Synthesis, Molecular Modeling and Biological Investigation
Source: Molecules. 2023 Feb 17;28(4):1945. doi: 10.3390/molecules28041945 (PMC9965125; doi:10.3390/molecules28041945)
Supplement: Supplementary file 1 [file molecules-28-01945-s001.zip › molecules-2188210-supplementary.pdf]

Supplementary Materials

Experimental

Biological studies

Anti-inflammatory activity screening

The anti-inflammatory activity of the tested compounds was determined *in-vivo* by the acute carrageenan induced paw edema standard method in rats [1-3]. Adult Wister rats of either sex (pregnant female animals were excluded) weighing 120–150 g were divided into 19 groups of 6 animals each. The tested compounds dissolved in DMSO, at a dose of 10 mg kg^{-1} (rat body weight) indomethacin mol equivalent, were given intraperitoneally 1 h before induction of inflammation. The control group was given DMSO only. Paw edema was induced by subcutaneous injection of freshly prepared 1% solution of carrageenan in saline (0.9%, 0.1 ml per rat) into subplantar tissue of the right hind paw of rats. The thickness of the paw was measured (in mm) after successive time intervals (1, 2, 3, 4 and 24 h) and compared with the initial hind paw thickness of each rat for determining the edema thickness. Data were collected, checked, revised and analyzed (SPSS 16). Quantitative variables from normal distribution were expressed as means ± SE "standard error". The anti-inflammatory activity was expressed as percentage inhibition of edema thickness in treated animals in comparison with the control group according to eqn. (1) (Table 1, Fig. 3).

$$\%\ Inhibition\ of\ edema = \frac{Vc-Vt}{Vc} \times 100 \quad \ldots\ldots\ldots\ldots\ldots\ldots\ldots\ldots\ (1)$$

Where, V_c and V_t are the means of edema paw thickness for the control and tested compound treated animal groups, respectively.

The potency of the tested compounds was expressed as % inhibition of edema thickness for the tested compounds relative to % inhibition of edema for indomethacin "reference standard" at 3 h effect "indomethacin reveals its maximum bio-properties at the mentioned time" according to eqn. (2) (Table 1).

$$\%\ \text{Potency} = \frac{\%\ \text{inhibition of edema for the tested compound treated group}}{\%\ \text{inhibition of edema for indomethacin treated group}} \times 100 \quad \ldots\ldots\ldots\ (2)$$

Analgesic properties

Peripheral analgesic testing

Peripheral analgesic activity was measured by the standard acetic acid-induced writhing test in mice [1,2]. Six albino mice of either sex (20–25 g) were used in each group (19 groups). One hour after intraperitoneally (i,p.) administration of 10 mg kg^{-1} (mice body weight) indomethacin mol equivalent of the test compound, ibuprofen or indomethacin suspended in distilled/sterile water by the aid of two drops of Tween 80, each mouse was injected with 0.1 ml of 1% acetic acid solution i.p. The control group animals were given sterile/distilled water only with few drops of Tween 80. Starting 5 min after the acetic acid injection, the number of muscular contractions in each mouse was counted for 30 min. A significant reduction in the number of writhing by any test compound as compared to control animals was considered as a positive analgesic response. Percentage protection was calculated according to equ. (3), where n stands for the average number of writhing in the control group and n' is the average number of writhing in the treated group. The % potency was calculated by equ. (4) (Table 2).

$$\% \text{ Inhibition/protection} = \frac{n-n'}{n} \text{x}100 \ \ldots\ldots\ldots. (3)$$

$$\% \text{ Potency} = \frac{\% \text{ protection of the tested compound}}{\% \text{ protection of indomethacin}} \text{ x } 100 \ \ldots\ldots (4)$$

Central analgesic (hot plate) testing

The standard hot plate technique was undertaken by in vivo standard hot plate test [1,2]. Six albino mice of either sex (20–25 g) were used in each group (19 groups). Mice were screened by placing them on a hot plate maintained at 55 ± 1ºC, 30 min. (LSI Letica hot plate LE-7406), after intraperitoneally (i.p.) administration of 10 mg kg^{-1} (mice body weight) indomethacin mol equivalent of the tested compounds and reference standards (indomethacin and ibuprofen) suspended in sterile/distilled water by the aid of two drops of Tween 80, and recording the reaction time in seconds for forepaw licking or jumping. The control group animals were given sterile/distilled water only with few drops of Tween 80. The maximum cutoff time was chosen as 15 sec. to prevent tissue damage. Response latencies were measured 30, 60, 90 and 120 min. after the application. The % protection was calculated by equ. (5) where, T_1, T_0 are the latency time mean of the tested compound and control group, respectively (Table 3).

$$\% \text{ Protection} = \frac{T_1 - T_0}{T_0} \text{x}100 \ldots\ldots\ldots\ldots\ldots\ldots\ldots\ldots (5)$$

Ulcerogenic liability

The ulcerogenic liability was determined in albino mice obeying the standard method [1,2]. Animals of either sex (pregnant females were excluded) weighing 20–25 g were divided into 8 groups of 6 animals each. The animals fasted 18 h before drug administration. The most promising anti-inflammatory active agents (**3**, **5a**, **5b**, **5d**, **5e**) in addition to the reference standard (indomethacin and ibuprofen) were suspended in distilled water by the aid of few drops of Tween 80 administered orally for three successive days to fasted animals at a dose of 10 mg kg^{-1} (animal body weight) indomethacin mol equivalent. The control group animals were given water only with few drops of Tween 80. The animals were sacrificed by cervical dislocation and the stomach was removed, opened along the greater curvature and rinsed with saline. The gastric mucosa was examined with a magnifying lens (10x) for the presence of lesions and erosions. The ulcer index was calculated (Table 4) and the degree of ulcerogenic effect was expressed in terms of.

1. The percentage incidence of ulcers divided by 10.
2. Average number of ulcers per stomach.
3. Average severity of ulcers.

The ulcer index is the value that resulted from the sum of the above three values.

Toxicological bio-assay

Toxicological study of the most promising anti-inflammatory active agents synthesized (**3**, **5a**, **5b**, **5d**, **5e**) was determined utilizing the standard method in mice [1,2]. Albino mice weighing 20–25 g were divided into 6 groups of 6 mice each. Administrations of the tested compounds dissolved in distilled water by the aid of few drops of Tween 80 were given intraperitoneally in 50 mg kg^{-1} (mice body weight) indomethacin mol equivalent (i.e. 5 folds of the used anti-inflammatory dosage). The control group was given water only with few drops of Tween 80. The toxic symptoms and mortality rates were recorded 24 h post-administration in each group.

COX-1 and COX-2 inhibitory properties

The inhibitory properties of the promising anti-inflammatory active agents synthesized (**3**, **5a**, **5b**, **5d**, **5e**) against COX-1 and COX-2 were undertaken by the standard techniques obeying the manufactor's instructions [4].

The COX-1/2 inhibitor screening protocols can be summarized in following:

The test compound is dissolved in DMSO. Dilute to 10X the desired test concentration with COX-1 Assay Buffer before use. Add 10 µl diluted test inhibitor or Assay Buffer into assigned wells as sample screen [S] or Enzyme Control [EC] (no inhibitor) respectively. Add 2 µl of SC560 (in case of COX-1 assay) or Celecoxib (in case of COX-2 assay) and 8 µl COX Assay Buffer into one of the wells as Inhibitor Control [IC].

Dilute COX Cofactor 200 times by adding 2 µl of COX Cofactor to 398 µl of COX Assay Buffer just before use. Mix well. Prepare Arachidonic Acid solution by adding 5 µl of supplied Arachidonic Acid to 5 µl of NaOH just before use. Vortex briefly to mix. Dilute Arachidonic Acid/NaOH solution 10 times by adding 90 µl ddH2O, vortex briefly to mix. Make as much as needed.

For each well, prepare 80 µl of master mix as follows:

	Reaction Master Mix
COX Assay Buffer	76 µl
COX Probe	1 µl
Diluted COX Cofactor	2 µl
COX-1	1 µl

Add 80 µl of Reaction Mix into each well. Use a multi-channel pipette to add 10 µl of diluted Arachidonic Acid/NaOH solution into each well to initiate all the reactions at the same time.

Measure fluorescence (Ex/Em = 535/587 nm) kinetically at 25°C for 5-10 min. Choose two points (T1 and T2) in the linear range of the plot and obtain the corresponding fluorescence values (RFU_1 and RFU_2).

Calculate the slope for all samples, including Enzyme Control (EC), by dividing the net ΔRFU ($RFU_2 - RFU_1$) values by the time ΔT ($T_2 - T_1$). Calculate % Relative Inhibition as follows:

$$\%\ Relative\ Inhibition = \frac{Slope\ of\ EC\ - Slope\ of\ S}{Slope\ of\ EC}\ X\ 100$$

Measurement of nitrite concentration using Griess method

The Griess method was used to determine the nitrite concentration in cell culture medium [5]. RAW264.7 macrophages were seeded at a density of 1×10^6 cells/mL in a 96-well plate for 2 h. The experimental data included normal control, LPS-stimulated cells only (10 ng/mL) and LPS-stimulated cells treated with the isolated ibuprofen conjugates 5a, 5b, 5d and 5e samples. Treatment of LPS-stimulated RAW264.7 macrophages was done with 40 µg/mL of the isolated ibuprofen conjugates and left overnight. The next day, a volume of 150 µL of the cell supernatant was transferred to a new 96-well plate and 130 µL of deionized water was added on top of it. Lastly, a volume of 20 µL of Griess reagent was added to the cell supernatant and incubated for 30 min in the dark at room temperature. Griess reagent was prepared according to protocol instructions: a 1:1 ratio of N-1-naphthyl-ethylenediamine (0.1%) and phosphoric acid (5%) in sulphanilamide (1%) in distilled water. After the incubation, the absorbance of azo chromophore formed color was measured spectrophotometrically at 548 nm (Nano SPECTROstar microplate reader (BMG LABTECH, Germany). The concentration of nitrite was finally measured using a $NaNO_2$ standard curve.

Measurement of cell viability using MTT assay

In order to ensure that the inflammatory response is not due to cell toxicity, cell viability measurements were done using MTT colorimetric assay. On the same cell cultured 96-well plate from the previous experiment, a concentration of 1 mg/mL and a volume 100 µL of MTT solution was added for 2h to the adherent RAW264.7 macrophages cells at 37°C. After that, 100 µL of DMSO was added to the wells where the insoluble formazan crystals were dissolved. Absorbance was the measured spectrophotometrically at 540 nm (NanoSPECTROstar microplate reader, BMG LABTECH, Germany). Cell viability was then calculated as a percentage of untreated control.

Evaluation of inflammatory mRNA expression levels

Inflammatory mRNA expression levels were evaluated using RT-qPCR (Applied Biosystems, CA, USA). Total RNA was extracted by lysing RAW264.7 cells using Qiazol Lysis Reagent. The extracted RNA was used to synthesize cDNA using RevertAid First Strand cDNA synthesis kit according to manufacturer's protocol. The cDNA was then added with forward and reverse

primers, nuclease free water and maxima SYBR green mix (Thermo Fisher Scientific, MA, USA). The inflammation-related genes of mRNA expression levels are as follows: IL-6, TNF-α, and iNOS. All expression was normalized to GAPDH (endogenous control) and relative fold mRNA expression levels were calculated using the comparative $2^{-\Delta\Delta CT}$ method. The PCR reaction consists of initiation stage at 95 °C for 10 min, after that 40 PCR cycles of denaturation stage for 15 s at 95 °C and annealing and extension for 1 min at 60 °C. NCBI Primer-Blast tool (https://www.ncbi.nlm.nih.gov/tools/primer-blast/) was used for primer generation (Thermo Fisher scientific, MA, USA). Table 6 shows primer sequence of the inflammation-related genes. Table S1 indicates the mRNA sequences used for the primers in RT-qPCR.

Table S1. mRNA sequences used for RT-qPCR.

Target mRNA	Primer sequence (5′–3′)		Tm
**IL-6**	Forward:	GATGCTACCAAACTGGATATAATCAG	55
	Reverse:	CTCTGAAGGACTCTGGCTTTG	58
TNF-α	Forward:	GAACTCCAGGCGGTGCCTAT	63
	Reverse:	TGAGAGGGAGGCCATTTGGG	63
iNOS	Forward:	GGAACCTACCAGCTCACTCTGG	63
	Reverse:	TGCTGAAACATTTCCTGTGCTGT	60
GAPDH	Forward:	CTTTGTCAAGCTCATTTCCTGG	57
	Reverse:	TCTTGCTCAGTGTCCTTGC	58

Docking studies

The compounds were energy minimized using the MacroModel (v9.9) molecular mechanics program and prepared for docking using the LigPrep module in the Schrödinger modeling package. Protein crystal structures were prepared using the protein preparation wizard. Hydrogens were added, bond orders were assigned, and the missing side chains and loops were added using the Prime package in Schrödinger. The hydrogen bonding network was optimized by reorienting the hydroxyl and thiol groups and amide groups of Asn, Gln, and His side chains. Neutral and protonated states of His, Asp, and Glu and tautomeric states of His were sampled at pH 7.0 using PROPKA. Following H-bond optimization, the protein was minimized using the OPLS-2005 force field until the RMSD of heavy atoms converged to 0.30 Å. The receptor grid

was constructed, and the docking site was set around the crystalized ligand without constraints. Ligands with a length up to 20 Å were allowed to dock. We used Glide XP for pose generation, and the docking was terminated if two consecutive solutions were within an RMSD of 0.5 Å. The docked poses were again minimized using in Prime, using the VSGB solvation model, and the energies were calculated using the OPLS3e force field. Residues within 5 Å of the ligand were treated as flexible.

2D-QSAR studies

The geometry of the tested conjugates were optimized by the molecular mechanics force field (MM^+), followed by semi-empirical AM1 method implemented in the HyperChem 8.0 package (http://www.hyper.com). The structures were fully optimized without constraining any parameters, thus bringing all geometric variables to their equilibrium values. The energy minimization protocol employed the Polak–Ribiere conjugated gradient algorithm [6]. Convergence to a local minimum was achieved when the energy gradient was $\leq$0.01 kcal mol^{-1}. The RHF (Restricted Hartree–Fock) method was used in the spin pairing for the semi-empirical tool.

2D-QSAR studies were undertaken to utilize the comprehensive descriptors for structural and statistical analysis (CODESSA-Pro) software [2]. The optimized structures of the tested bio-active compounds were uploaded to CODESSA-Pro that includes MOPAC capability for the final geometry optimization. CODESSA-Pro calculated molecular descriptors (constitutional, topological, geometrical, charge-related, semi-empirical, thermodynamical, molecular-type, atomic-type and bond-type descriptors) for the exported agents. Different mathematical transformations [including property, 1/property, log(property) and 1/log(property)] of the experimentally observed property/activity of the training set compounds were utilized searching for the best QSAR model. The best multi-linear regression (BMLR) technique was utilized which is a stepwise search for the best *n* parameter regression equations (where *n* stands for the number of descriptors used), based on the highest R^2 (squared correlation coefficient), R^2cvOO (squared cross-validation "leave-one-out, LOO" coefficient), R^2cvMO (squared cross-validation "leave-many-out, LMO" coefficient), *F* (Fisher statistical significance criteria) values, and *s* (standard

deviation). The QSAR models describing the bioactivity of the agents were generated (obeying the thumb rule) (Tables S2–S10, Fig. S1-S3).

References

[1] Panda, S.S.; Girgis, A.S.; Honkanadavar, H.H.; George, R.F.; Srour, A.M. Synthesis of new ibuprofen hybrid conjugates as potential anti-inflammatory and analgesic agents. *Futur. Med. Chem.* **2020**, *12*, 1369–1386, https://doi.org/10.4155/fmc-2020-0109.

[2] Panda, S.S.; Girgis, A.S.; Thomas, S.J.; Capito, J.E.; George, R.F.; Salman, A.; El-Manawaty, M.A.; Samir, A. Synthesis, pharmacological profile and 2D-QSAR studies of curcumin-amino acid conjugates as potential drug candidates. *Eur. J. Med. Chem.* **2020**, *196*, 112293, https://doi.org/10.1016/j.ejmech.2020.112293.

[3] Girgis, A.S.; Tala, S.R.; Oliferenko, P.V.; Oliferenko, A.A.; Katritzky, A.R. Computer-assisted rational design, synthesis, and bioassay of non-steroidal anti-inflammatory agents. *Eur. J. Med. Chem.* **2012**, *50*, 1–8, https://doi.org/10.1016/j.ejmech.2011.11.034.

[4] *COX-1/2 (Human) Inhibitor Screening Assay Kits (Fluorometric, Catalog # K547-100 and K548-100)*; BioVision Incorporated: Milpitas, CA, USA. Available online: www.biovision.com (accessed on).

[5] Wang, W.; Liu, P.; Hao, C.; Wu, L.; Wan, W.; Mao, X. Neoagaro-oligosaccharide monomers inhibit inflammation in LPS-stimulated macrophages through suppression of MAPK and NF-κB pathways. *Sci. Rep.* **2017**, *7*, srep44252, https://doi.org/10.1038/srep44252.

[6] Nofal, Z.M.; Srour, A.M.; El-Eraky, W.I.; Saleh, D.O.; Girgis, A.S. Rational design, synthesis and QSAR study of vasorelaxant active 3-pyridinecarbonitriles incorporating 1H-benzimidazol-2-yl function. *Eur. J. Med. Chem.* **2013**, *63*, 14–21, https://doi.org/10.1016/j.ejmech.2013.01.042.

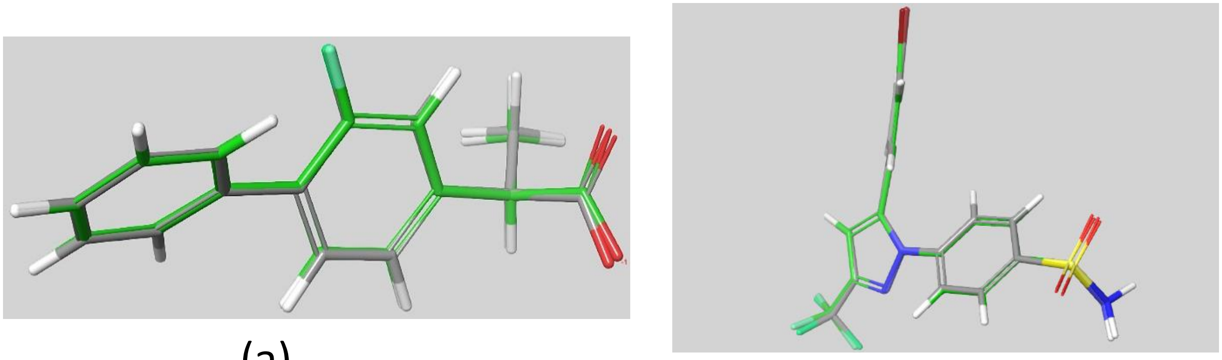

(a) (b)

Figure S1. (a) Overlay of the bioactive conformation of Flurbiprofen (green) with the Glide XP docked pose of the ligand in COX-1 crystal structure, PDB entry 3N8W; (b) Overlay of the bioactive conformation of SC-558 (green) with the Glide XP docked pose of the ligand in COX-2 crystal structure, PDB entry 6COX.

Table S2. Descriptors of the QSAR model for the tested anti-inflammatory active agents.

Entry	ID	Coefficient	*s*	*t*	Descriptor
1	0	2.4754	0.045	54.69	Intercept
2	D_1	-0.0148805	0.003	-5.545	H-donors PSA (version 2)
3	D_2	-0.0021036	0.0001	-13.16	WNSA-1 Weighted PNSA (PNSA1*TMSA/1000) (MOPAC PC)
4	D_3	-0.0598335	0.004	-13.426	RNCS Relative negative charged SA (SAMNEG*RNCG) (Zefirov PC)

$N = 12$, $n = 3$, $R^2 = 0.979$, R^2cvOO = 0.951, R^2cvMO = 0.963, $F = 123.9$, $s^2 = 0.001$

log(% inhibition of edema thickness at 3h) = 2.4754 – (0.0148805 x D_1) – (0.0021036 x D_2) – (0.0598335 x D_3)

Table S3. Observed and estimated anti-inflammatory properties for the tested compounds according to the BMLR-QSAR model.

Entry	Compd.	log(observed property)	Observed property	log(estimated property)	Estimated property	Error[a]
1	**3**	1.93349	85.8	1.93775	86.6	-0.8
2	**5a**	1.97955	95.4	1.95999	91.2	4.2
3	**5b**	1.97543	94.5	1.93178	85.5	9.0
4	**5c**	1.43297	27.1	1.41998	26.3	0.8
5	**5d**	1.88138	76.1	1.92863	84.8	-8.7
6	**5e**	1.94694	88.5	1.95295	89.7	-1.2
7	**5f**	1.76716	58.5	1.77554	59.6	-1.1
8	**5g**	1.8169	65.6	1.82804	67.3	-1.7
9	**8a**	1.51188	32.5	1.48976	30.9	1.6
10	**8c**	1.6464	44.3	1.63371	43.0	1.3
11	**8e**	1.4133	25.9	1.4783	30.1	-4.2
12	**8g**	1.56703	36.9	1.536	34.4	2.5

[a] Error is the difference between the observed and estimated property.

Table S4. Molecular descriptor values of the QSAR model for the tested compounds.

Entry	Compd	Descriptors[a]		
		D_1	D_2	D_3
1	**3**	11.92823	89.51916	2.87195
2	**5a**	14.79101	140.3812	0
3	**5b**	12.88249	163.8141	0.1223
4	**5c**	11.92823	148.1222	9.46504
5	**5d**	18.60805	121.181	0.24984
6	**5e**	1.90852	150.1909	2.97673
7	**5f**	12.88249	136.0808	3.70859
8	**5g**	9.06546	216.654	0.94766
9	**8a**	15.74527	271.1091	3.02568
10	**8c**	12.88249	248.1973	2.13735
11	**8e**	14.31388	298.4915	2.61049
12	**8g**	18.13092	289.9739	0.99622

[a]D_1 = H-donors PSA (version 2), D_2 = WNSA-1 Weighted PNSA (PNSA1*TMSA/1000) (MOPAC PC), D_3 = RNCS Relative negative charged SA (SAMNEG*RNCG) (Zefirov PC).

Table S5. Descriptors of the QSAR model for the tested peripheral analgesic active agents.

Entry	ID	Coefficient	*s*	*t*	Descriptor
1	0	2.58125	0.448	5.757	Intercept
2	D_1	-0.981123	0.203	-4.827	Avg. nucleoph. react. index for atom N
3	D_2	-0.323865	0.057	-5.659	Max. atomic state energy for atom H
4	D_3	-1.28007	0.158	-8.096	Max. electroph. react. index for atom C

N = 14, n = 3, R^2 = 0.895, R^2cvOO = 0.811, R^2cvMO = 0.831, F = 28.355, s^2 = 2.435e-006

1/(% inhibition) = 2.58125 – (0.981123 x D_1) – (0.323865 x D_2) – (1.28007 x D_3)

Table S6. Observed and estimated peripheral analgesic properties for the tested compounds according to the BMLR-QSAR model.

Entry	Compd.	1/(observed property)	Observed property	1/(estimated property)	Estimated property	Error[a]
1	**5a**	0.012005	83.3	0.013395	74.7	8.6
2	**5b**	0.011429	87.5	0.012091	82.7	4.8
3	**5c**	0.012005	83.3	0.011625	86.0	-2.7
4	**5d**	0.012121	82.5	0.012785	78.2	4.3
5	**5e**	0.015798	63.3	0.014143	70.7	-7.4
6	**5f**	0.0125	80.0	0.014098	70.9	9.1
7	**5g**	0.015385	65.0	0.013976	71.6	-6.6
8	**8a**	0.020284	49.3	0.021096	47.4	1.9
9	**8b**	0.021598	46.3	0.020818	48.0	-1.7
10	**8c**	0.024213	41.3	0.023524	42.5	-1.2
11	**8d**	0.016	62.5	0.01749	57.2	5.3
12	**8e**	0.012376	80.8	0.011431	87.5	-6.7
13	**8f**	0.01497	66.8	0.012473	80.2	-13.4
14	**8g**	0.010384	96.3	0.012123	82.5	13.8

[a] Error is the difference between the observed and estimated property.

Table S7. Molecular descriptor values of QSAR model for the peripheral analgesic active agents.

Entry	Compd	Descriptors[a]		
		D_1	D_2	D_3
1	**5a**	0.00012	7.8146	0.0288
2	**5b**	0.00595	7.815	0.02525
3	**5c**	0.00721	7.8103	0.02583
4	**5d**	0.00734	7.8159	0.02341
5	**5e**	0.00499	7.8144	0.02453
6	**5f**	0.00529	7.8237	0.02198
7	**5g**	0	7.8129	0.02886
8	**8a**	0.00364	7.8133	0.02041
9	**8b**	0.00259	7.8191	0.01997
10	**8c**	0.00365	7.7989	0.02215
11	**8d**	0.00209	7.8229	0.02199
12	**8e**	0.00389	7.8328	0.02284
13	**8f**	0.00365	7.8211	0.02517
14	**8g**	0.00278	7.8116	0.02851

[a]D_1 = Avg. nucleoph. react. index for atom N, D_2 = Max. atomic state energy for atom H, D_3 = Max. electroph. react. index for atom C.

Table S8. Descriptors of the QSAR model for the tested central analgesic active agents.

Entry	ID	Coefficient	*s*	*t*	Descriptor
1	0	-4500.61	371.777	-12.1057	Intercept
2	D_1	402.343	32.617	12.335	Max. resonance energy for bond H-C
3	D_2	615.863	93.4621	6.589	FNSA-3 Fractional PNSA (PNSA-3/TMSA) (MOPAC PC)
4	D_3	-8834.15	716.006	-12.3381	Max. 1-electron react. index for atom N

N = 14, n = 3, R^2 = 0.967, R^2cvOO = 0.941, R^2cvMO = 0.949, F = 97.863, s^2 = 20.067

% Protection = –4500.61 + (402.343 x D_1) + (615.863 x D_2) – (8834.15 x D_3)

Table S9. Observed and estimated central analgesic properties for the tested compounds according to the QSAR model.

Entry	Compd.	Observed property	Estimated property	Error[a]
1	**5a**	54.5	49.5	5.0
2	**5b**	27.1	28.0	-0.9
3	**5c**	18	20.5	-2.5
4	**5d**	1.2	-2.1	3.3
5	**5e**	28	31.3	-3.3
6	**5f**	0.2	2.1	-1.9
7	**5g**	28.1	25.2	2.9
8	**8a**	51.3	46.1	5.2
9	**8b**	9.7	13.3	-3.6
10	**8c**	48.1	44.3	3.8
11	**8d**	48.6	56.4	-7.8
12	**8e**	28.9	25.5	3.4
13	**8f**	66.5	68.4	-1.9
14	**8g**	2.7	4.4	-1.7

[a] Error is the difference between the observed and estimated property.

Table S10. Molecular descriptor values of the QSAR model for the central analgesic tested compounds.

Entry	Compd.	Descriptors[a]		
		D_1	D_2	D_3
1	**5a**	11.4069	-0.03877	0.00176
2	**5b**	11.4186	-0.03964	0.00466
3	**5c**	11.4263	-0.04921	0.00519
4	**5d**	11.3564	-0.03786	0.00536
5	**5e**	11.3569	-0.04154	0.00134
6	**5f**	11.358	-0.04395	0.00453
7	**5g**	11.3875	-0.08934	0.0001
8	**8a**	11.413	-0.05235	0.00147
9	**8b**	11.3319	-0.05631	0.00121
10	**8c**	11.3948	-0.06407	0.00003
11	**8d**	11.4089	-0.05398	0
12	**8e**	11.3236	-0.04844	0
13	**8f**	11.44	-0.0549	0
14	**8g**	11.3328	-0.08776	0.00007

[a]D_1 = Max. resonance energy for bond H-C, D_2 = FNSA-3 Fractional PNSA (PNSA-3/TMSA) (MOPAC PC), D_3 = Max. 1-electron react. index for atom N.

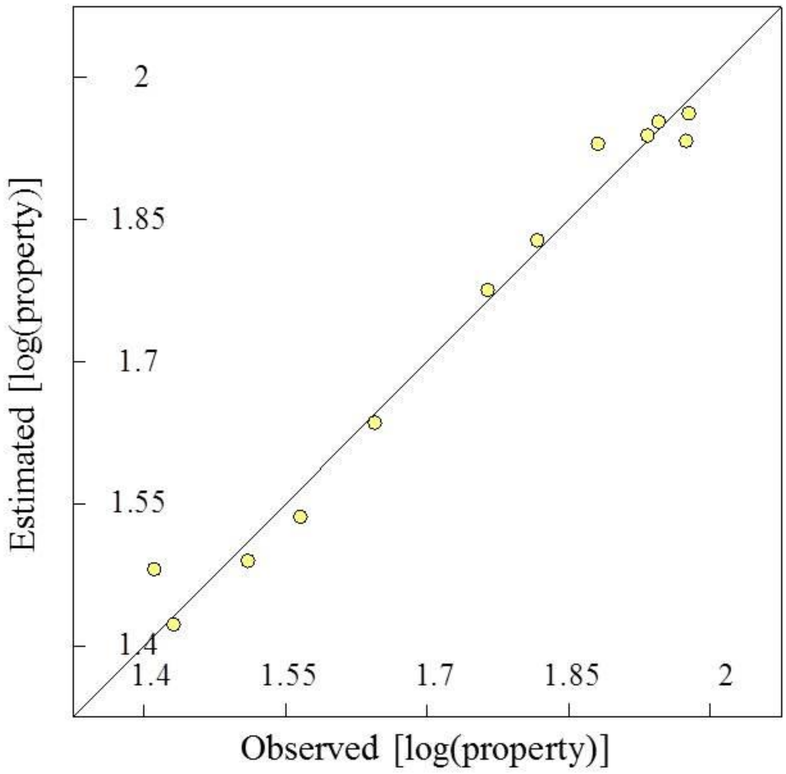

Fig. S2. QSAR plot representing the observed versus predicted log[% inhibition of edema thickness for the tested compounds at 10 mg/kg (rat body weight) indomethacin mol equivalent at 3 h effect].

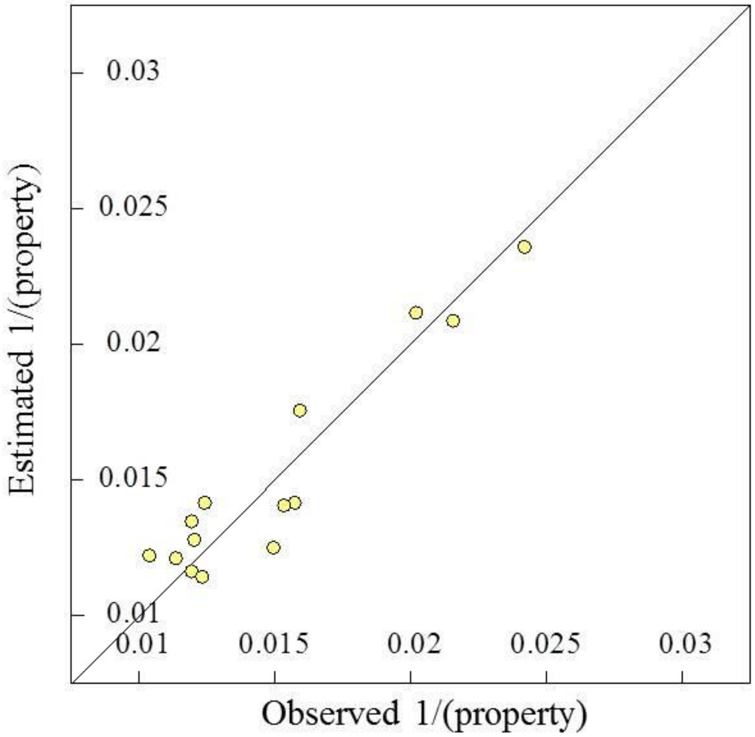

Fig. S3. QSAR plot representing the observed versus predicted 1/property "% inhibition of peripheral analgesic properties for the tested compounds at 10 mg/kg (rat body weight) indomethacin mol equivalent".

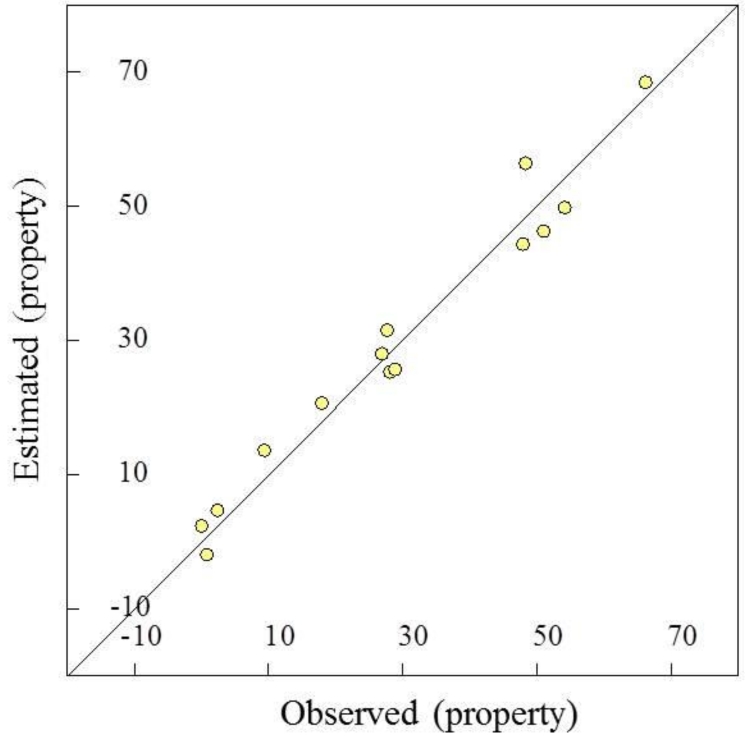

Fig. S4. QSAR plot representing the observed versus predicted property "% protection for the central analgesic tested compounds at 10 mg/kg (rat body weight) indomethacin mol equivalent".

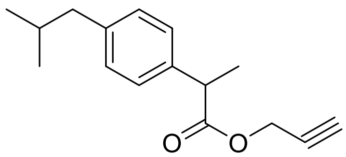

3

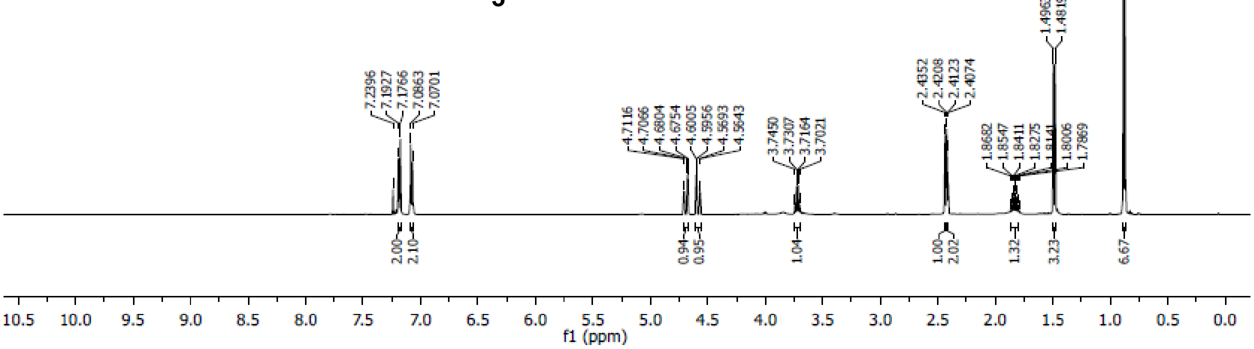

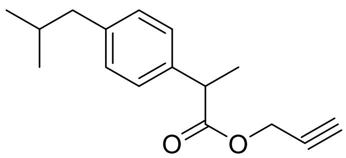

3

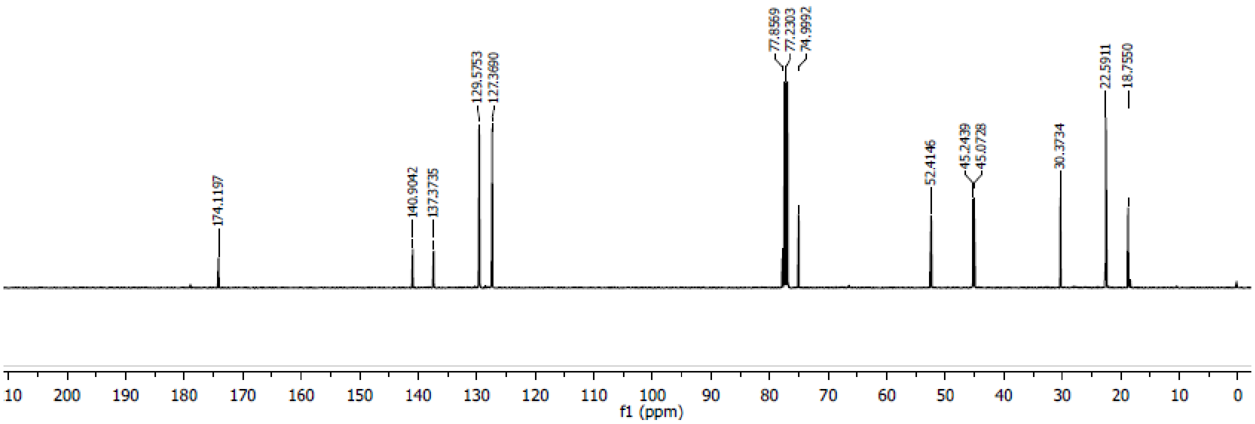

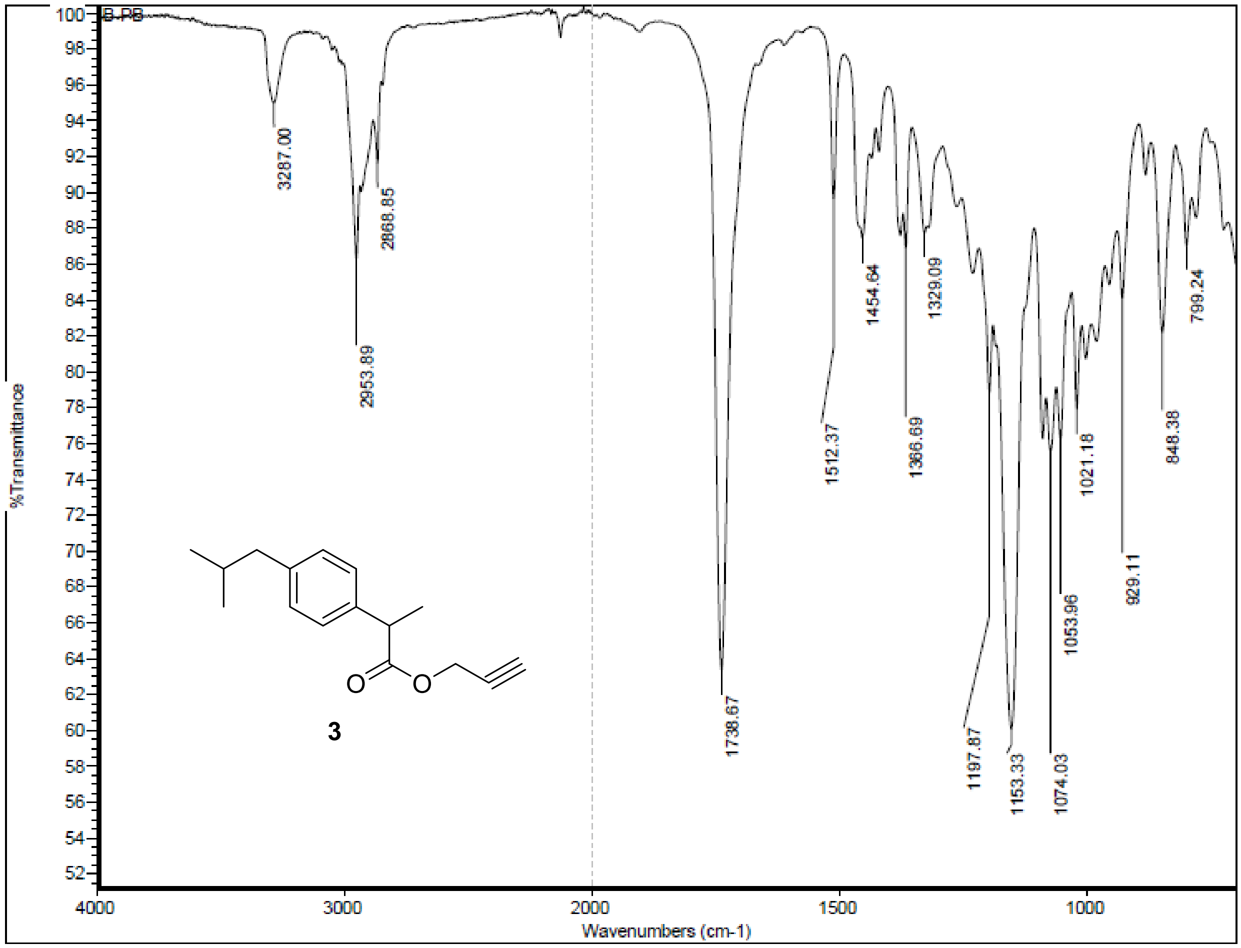
IB-PB
%Transmittance
Wavenumbers (cm-1)
3287.00
2953.89
2868.85
1738.67
1512.37
1454.64
1366.69
1329.09
1197.87
1153.33
1074.03
1053.96
1021.18
929.11
848.38
799.24
3

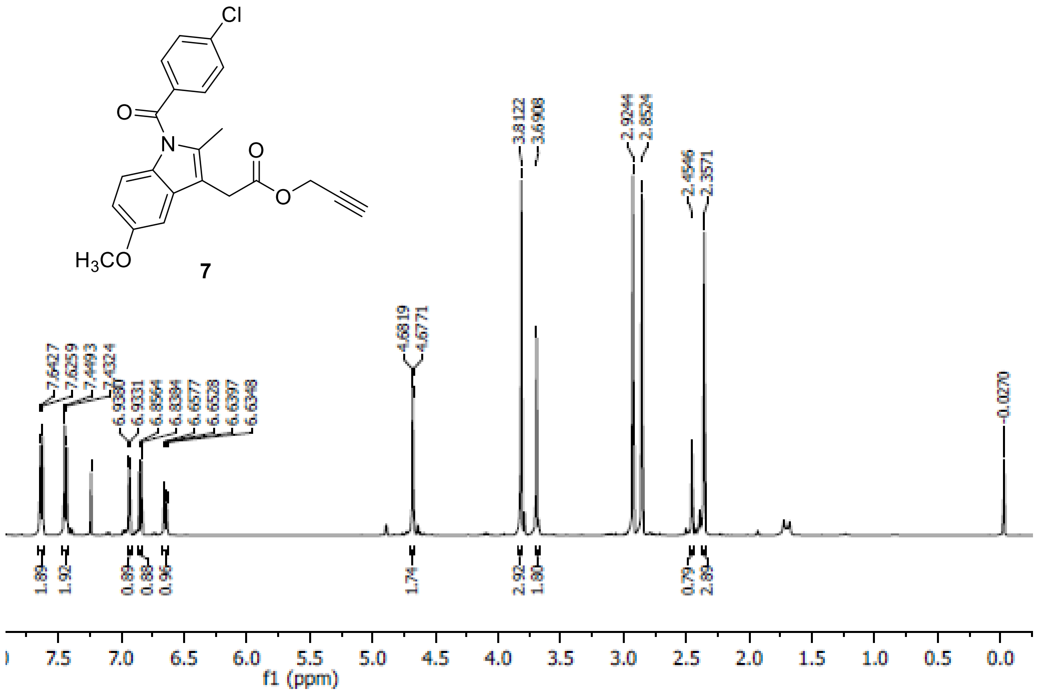

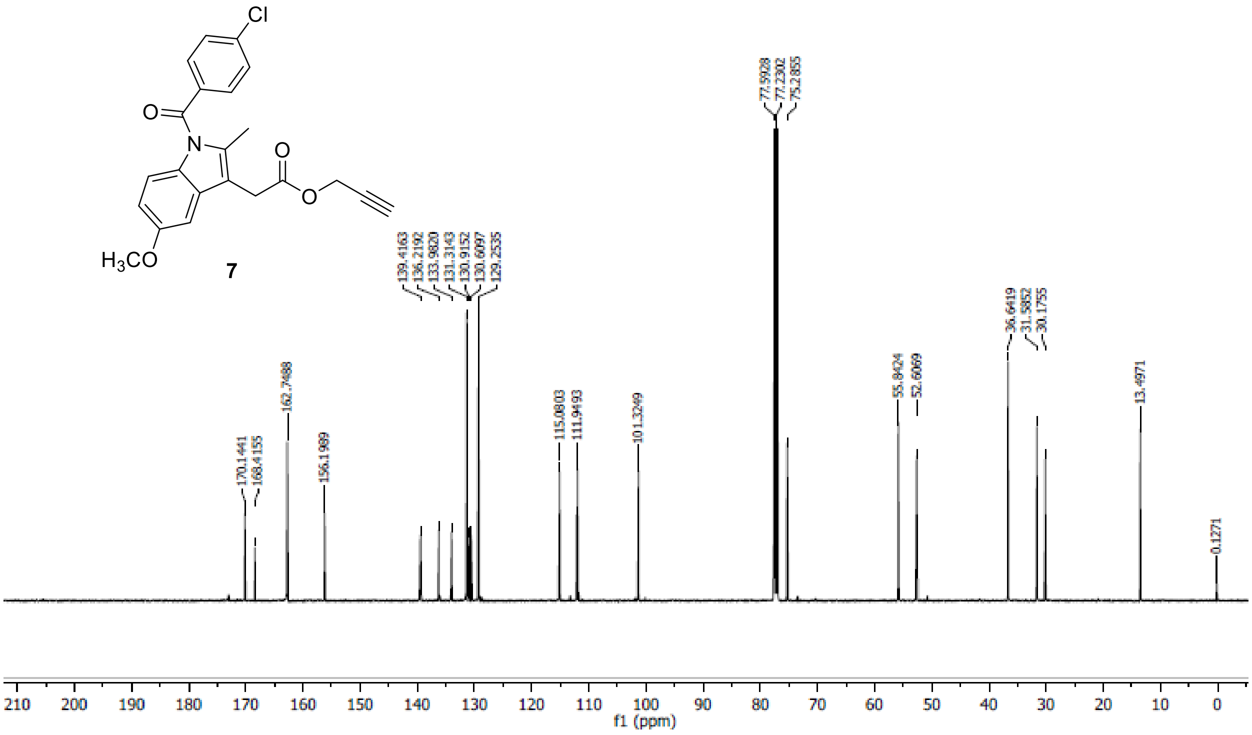

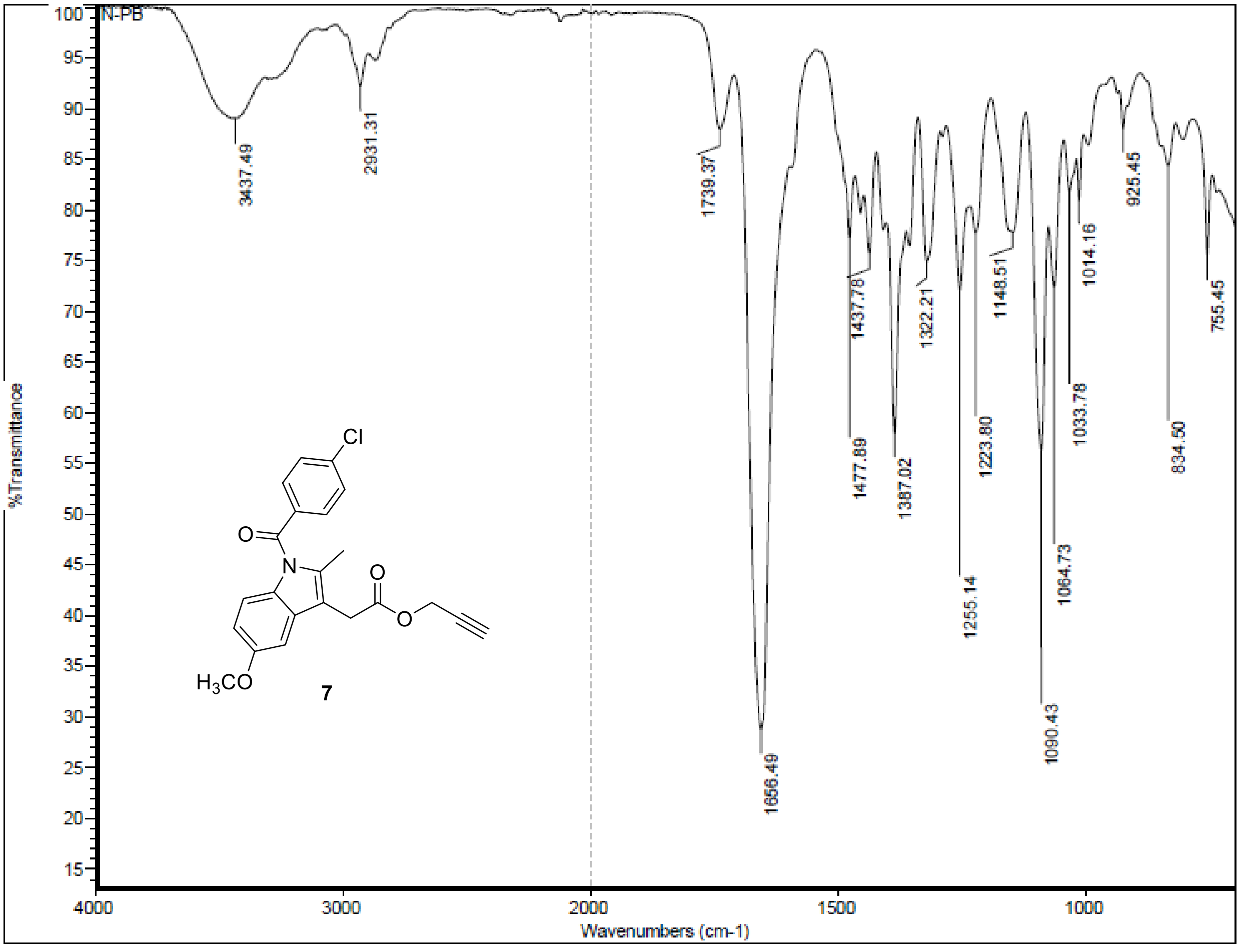

N-PB
%Transmittance
Wavenumbers (cm-1)
3437.49
2931.31
1739.37
1656.49
1477.89
1437.78
1387.02
1322.21
1255.14
1223.80
1148.51
1090.43
1064.73
1033.78
1014.16
925.45
834.50
755.45
Cl
O
N
O
O
H3CO
7

5a

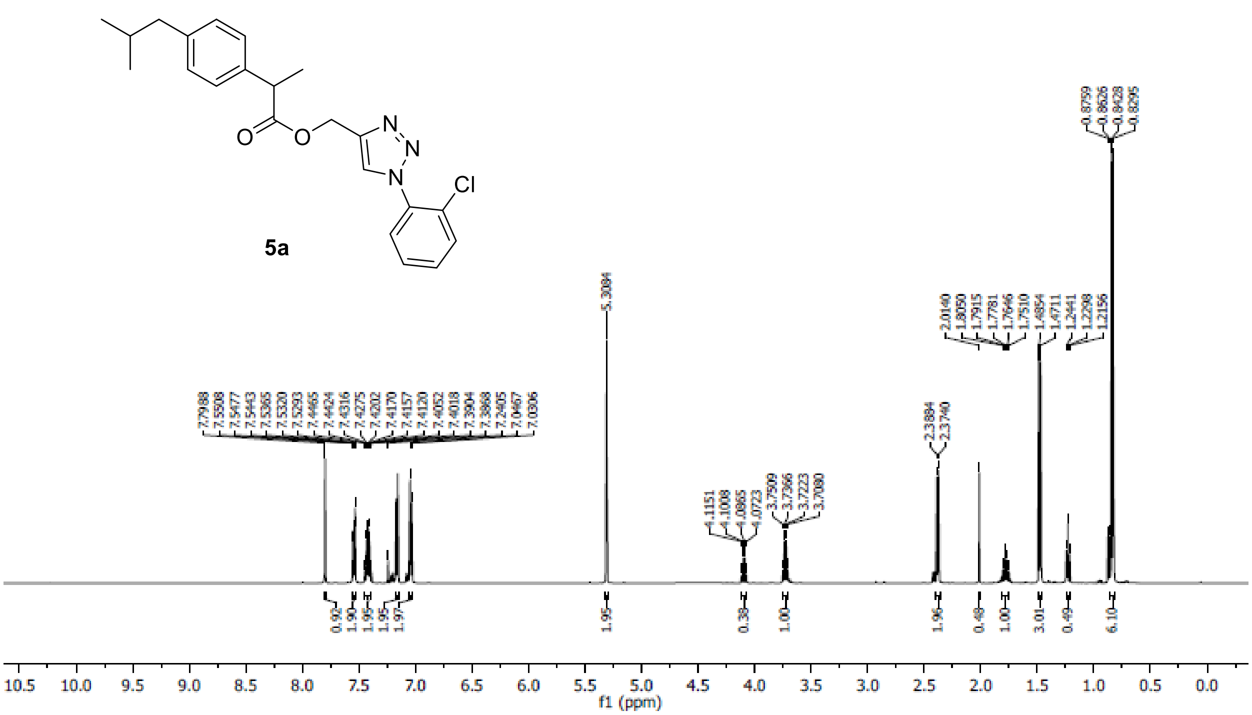

5a

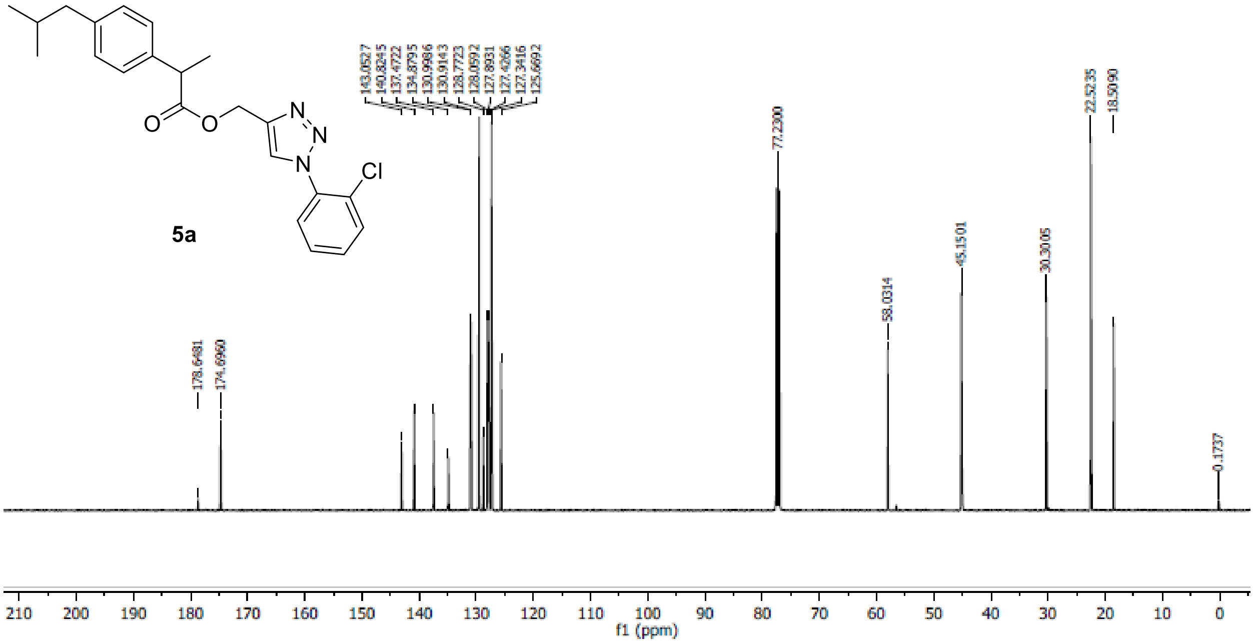

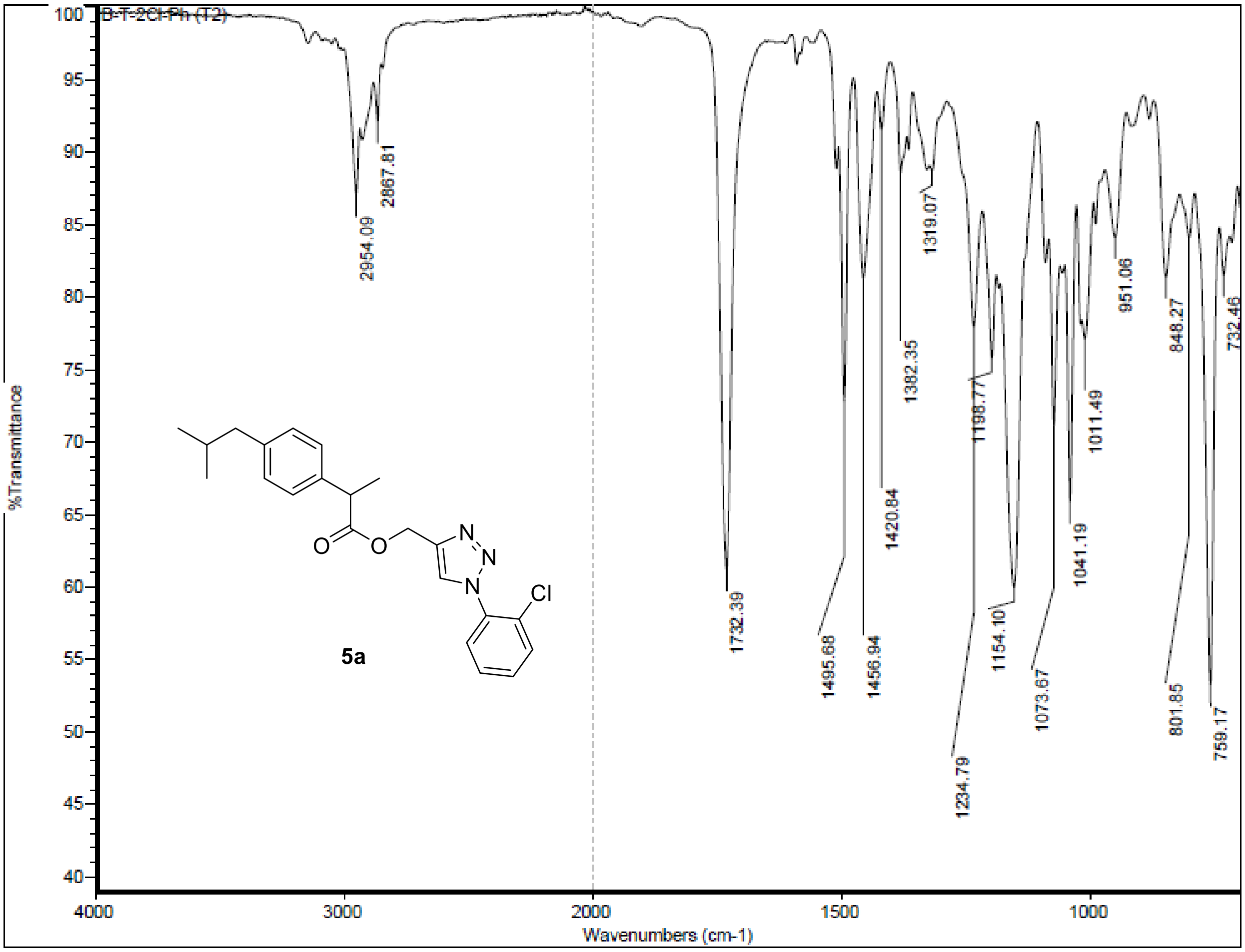
B-T-2Cl-Ph (T2)
%Transmittance
Wavenumbers (cm-1)
2954.09
2867.81
1732.39
1495.68
1456.94
1420.84
1382.35
1319.07
1234.79
1198.77
1154.10
1073.67
1041.19
1011.49
951.06
848.27
801.85
759.17
732.46
O
O
N
N
N
Cl
5a

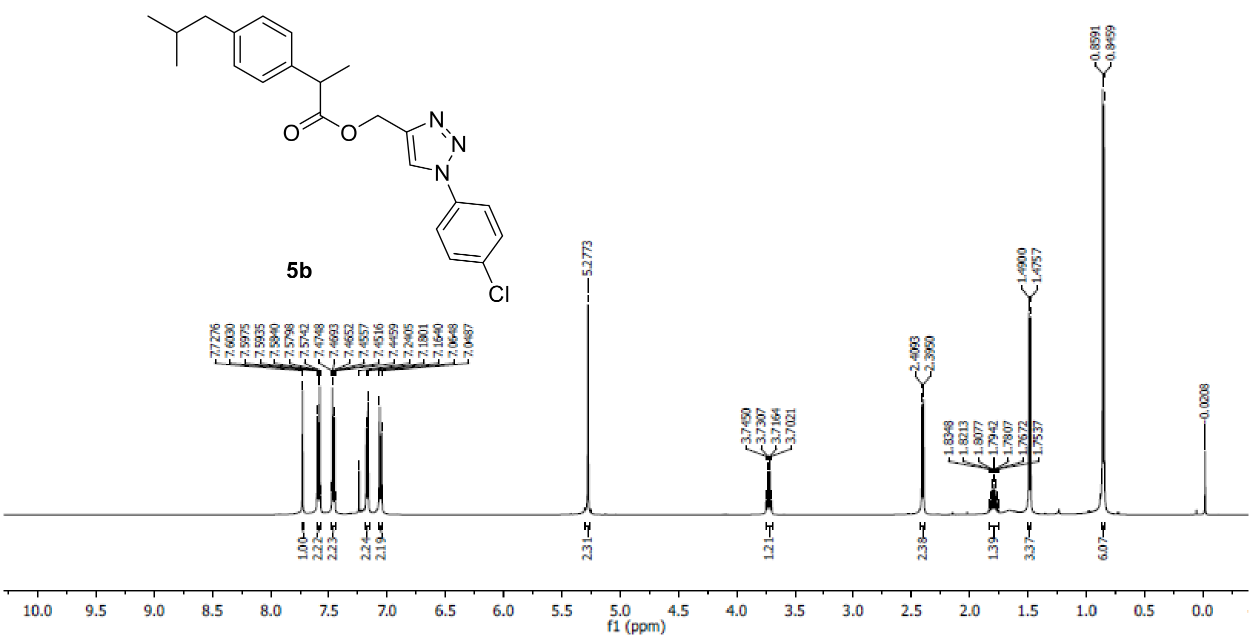
5b
Cl
f1 (ppm)

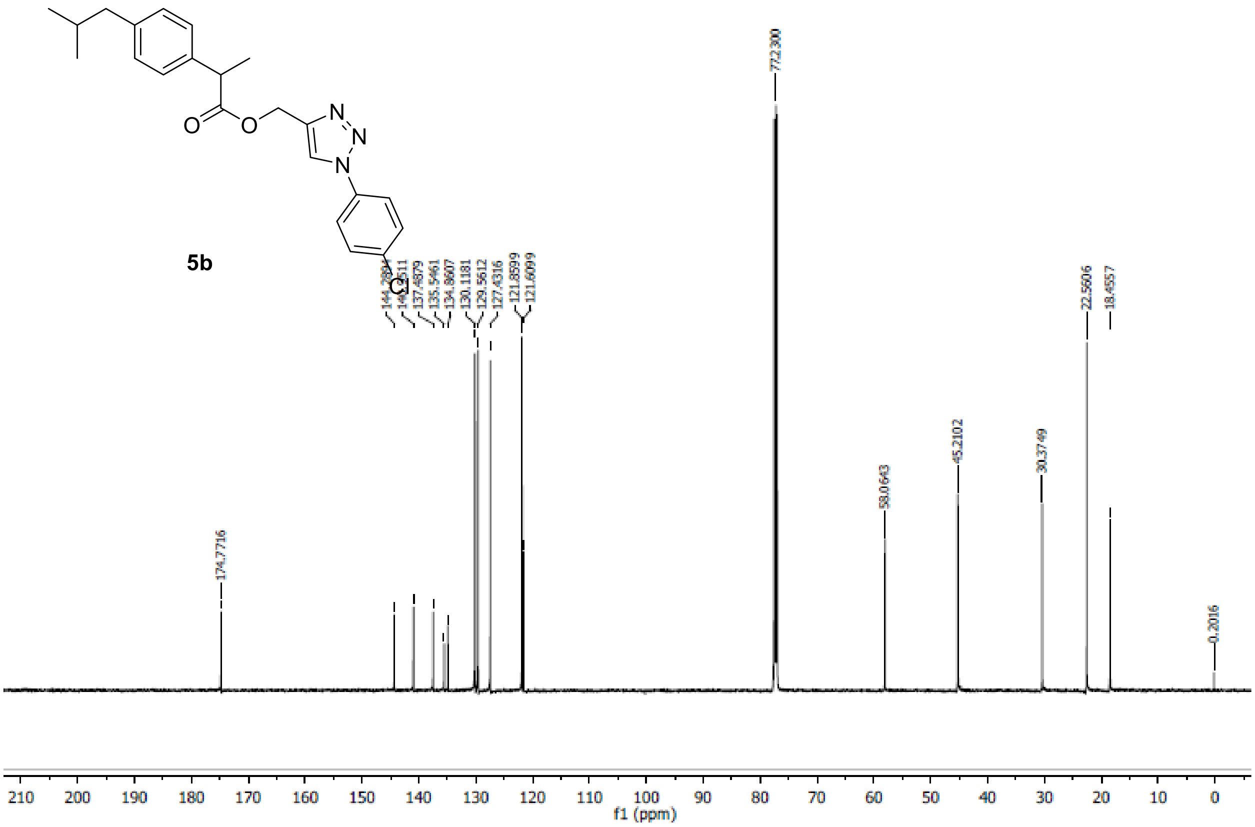
5b
f1 (ppm)

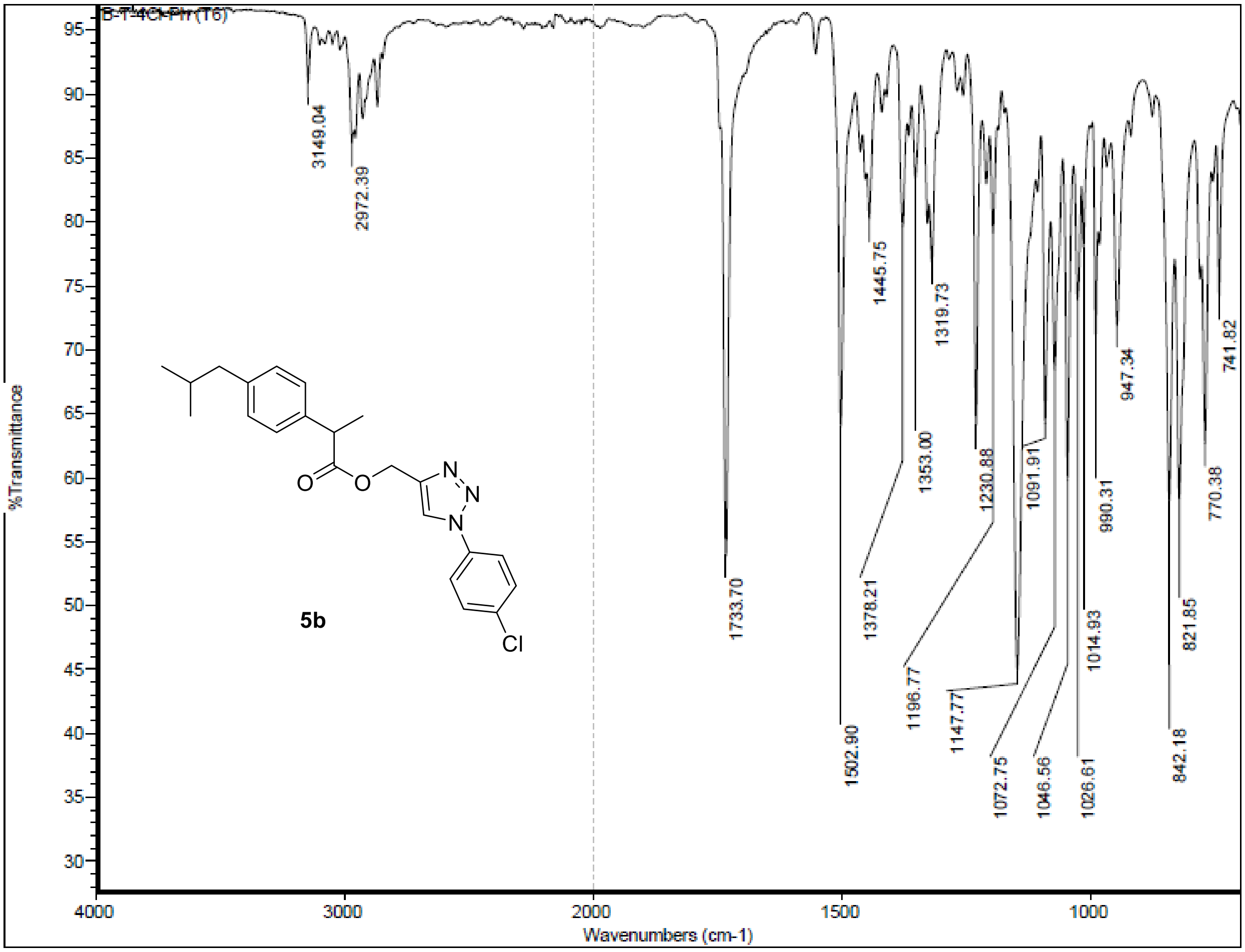
B-T-4Cl-Ph (T6)
%Transmittance
Wavenumbers (cm-1)
5b
3149.04
2972.39
1733.70
1502.90
1445.75
1378.21
1353.00
1319.73
1230.88
1196.77
1147.77
1091.91
1072.75
1046.56
1026.61
1014.93
990.31
947.34
842.18
821.85
770.38
741.82

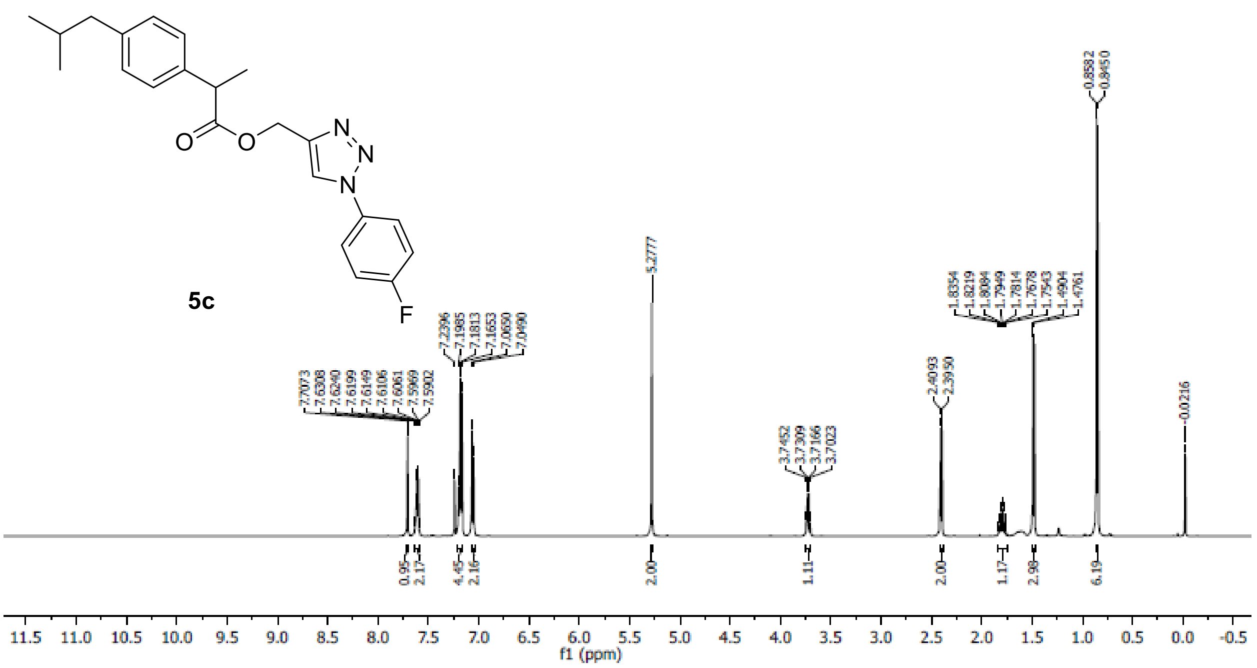
5c
O
N
F
7.7073
7.6308
7.6240
7.6199
7.6149
7.6106
7.6061
7.5969
7.5902
7.2296
7.1985
7.1813
7.1653
7.0650
7.0490
5.2777
3.7452
3.7309
3.7166
3.7023
2.4093
2.3950
1.8354
1.8219
1.8084
1.7949
1.7814
1.7678
1.7543
1.4904
1.4761
0.8582
0.8450
0.0216
0.95
2.17
4.45
2.16
2.00
1.11
2.00
1.17
2.98
6.19
f1 (ppm)

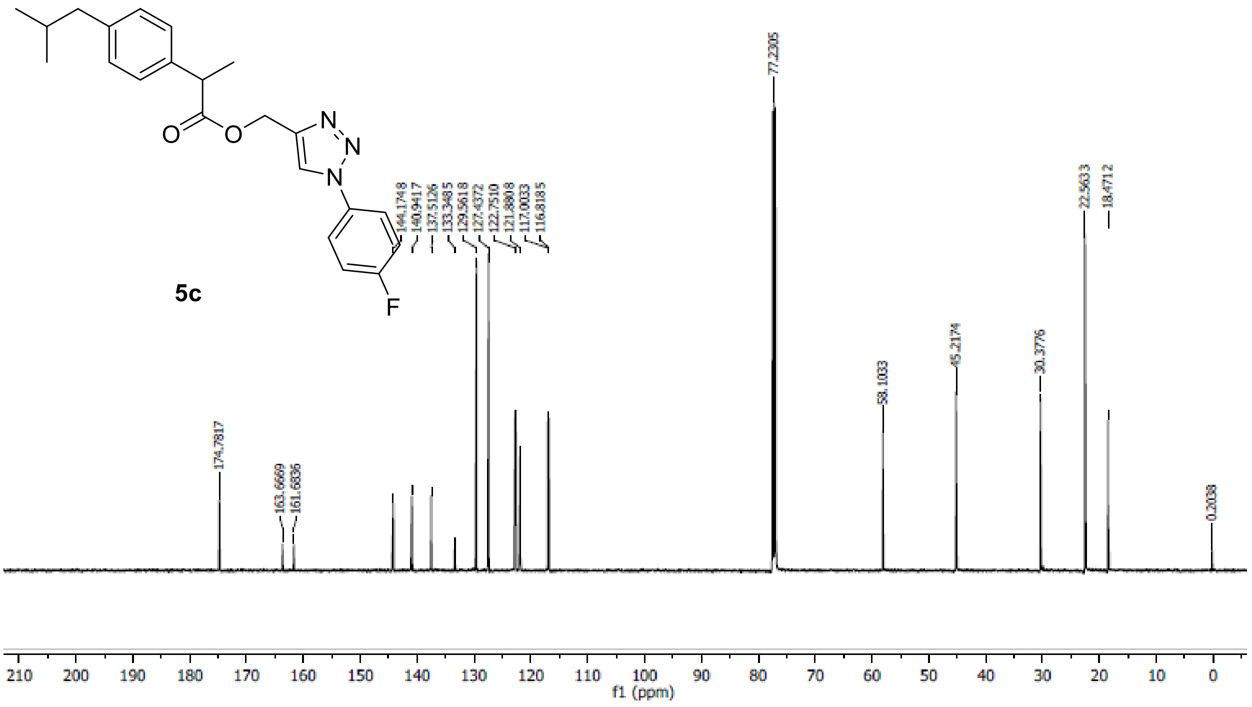
5c
O
N
F
174.7817
163.6669
161.6836
144.1748
140.9417
137.5126
133.3485
129.5618
127.4372
122.7510
121.8808
117.0033
116.8185
77.2305
58.1033
45.2174
30.3776
22.5633
18.4712
0.2038
f1 (ppm)

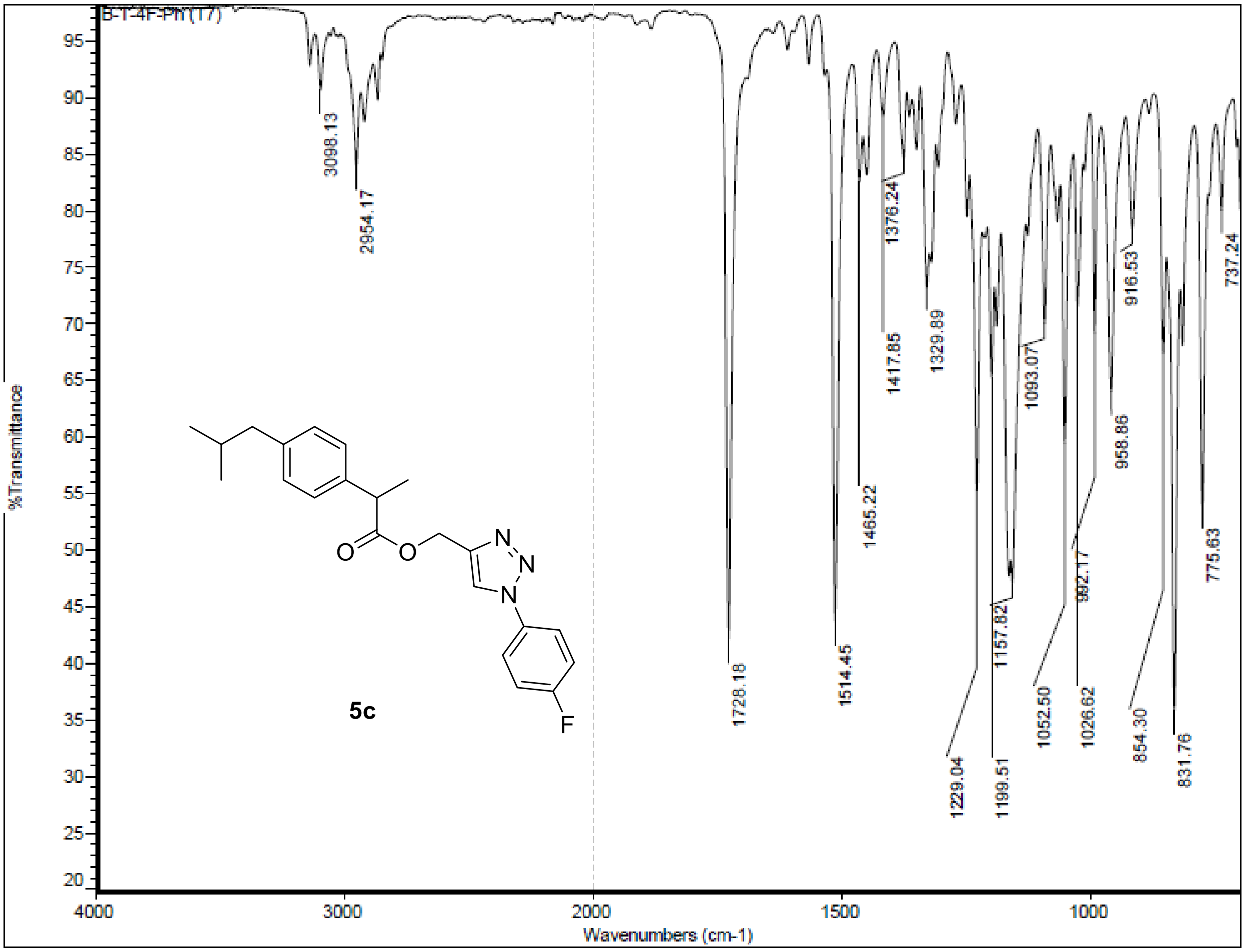
B-T-4F-Ph(17)
%Transmittance
Wavenumbers (cm-1)
4000
3000
2000
1500
1000
3098.13
2954.17
1728.18
1514.45
1465.22
1417.85
1376.24
1329.89
1229.04
1199.51
1157.82
1093.07
1052.50
1026.62
992.17
958.86
916.53
854.30
831.76
775.63
737.24
5c

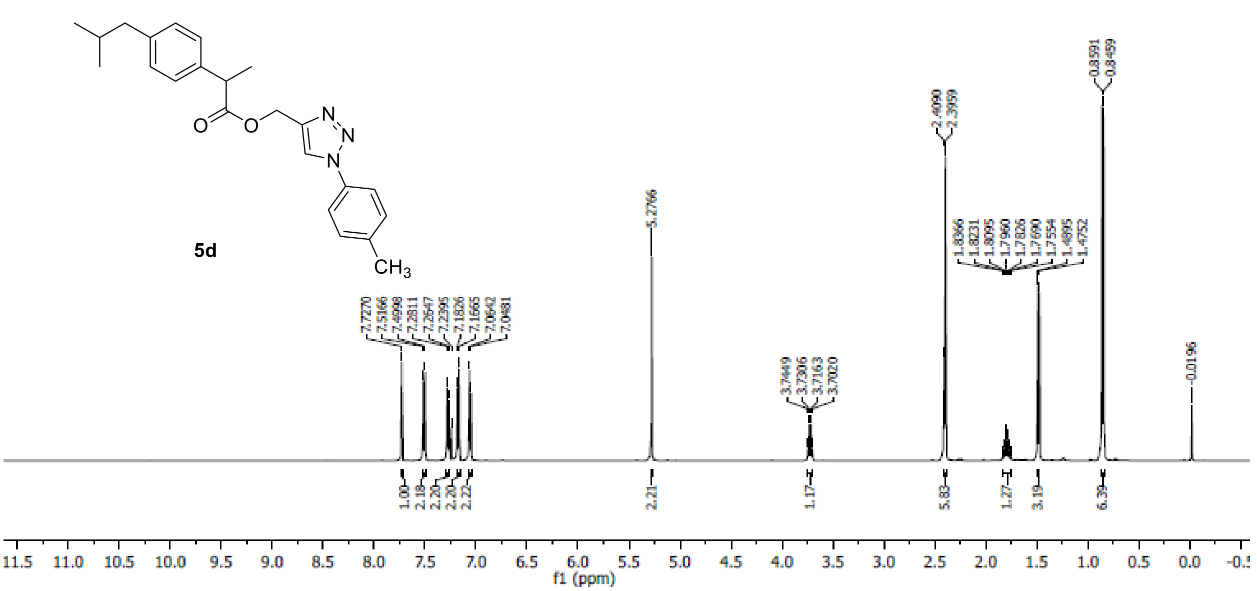
5d
7.7270 7.5166 7.4998 7.2811 7.2647 7.2395 7.1826 7.1665 7.0642 7.0481
5.2766
3.7449 3.7306 3.7163 3.7020
2.4090 2.3959
1.8366 1.8231 1.8095 1.7960 1.7826 1.7690 1.7554 1.4895 1.4752
0.8591 0.8459
0.0196
1.00 2.18 2.20 2.20 2.22 2.21 1.17 5.83 1.27 3.19 6.39
f1 (ppm)

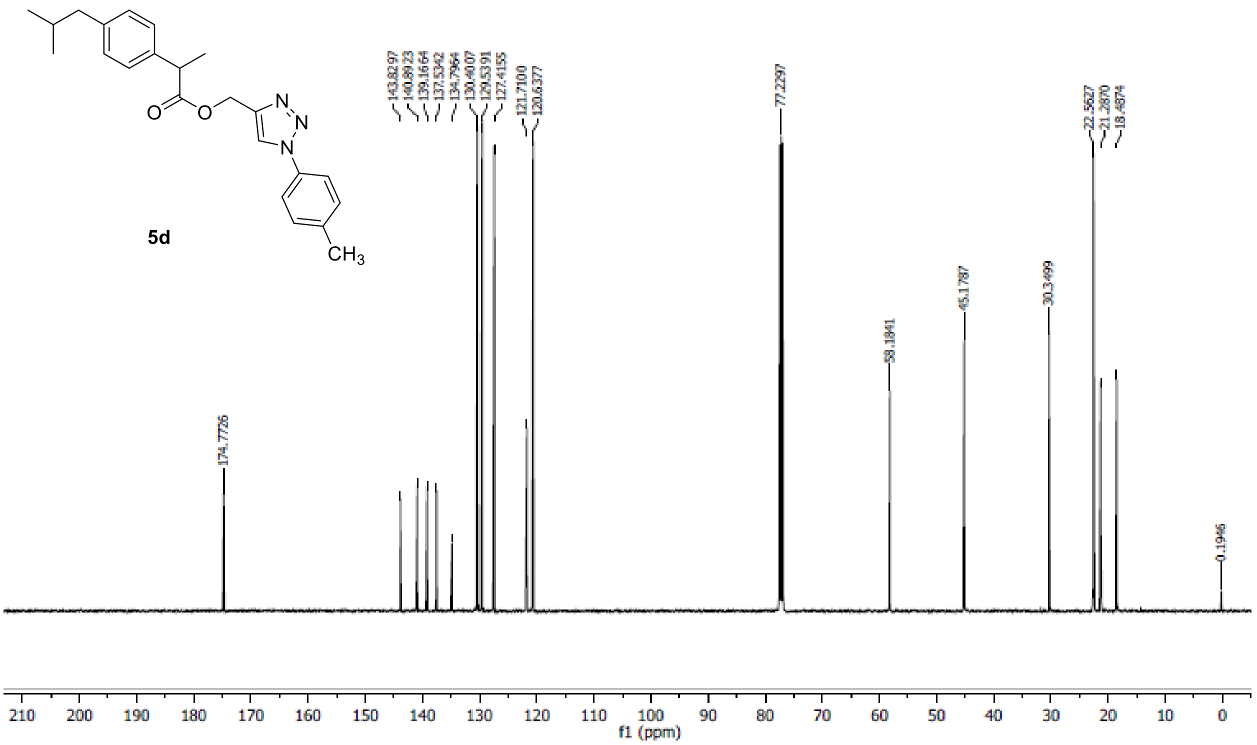
5d
174.7726
143.8297 140.8923 139.1664 137.5342 134.7964 130.4007 129.5391 127.4155 121.7100 120.6377
77.2297
58.1841
45.1787
30.3499
22.5627 21.2870 18.4874
0.1946
f1 (ppm)

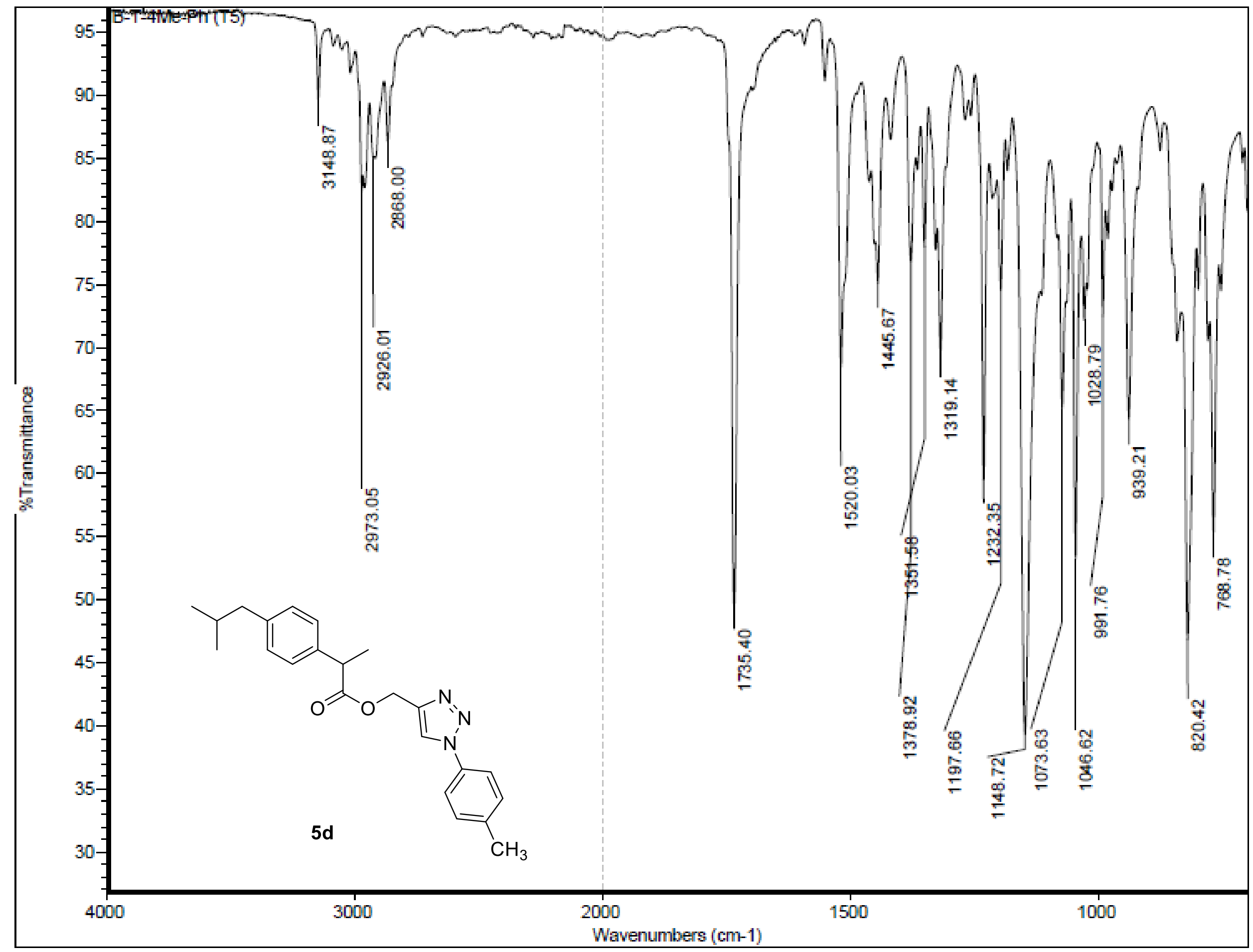
B-T-4Me-Ph (T5)
%Transmittance
Wavenumbers (cm-1)
3148.87
2973.05
2926.01
2868.00
1735.40
1520.03
1445.67
1378.92
1351.58
1319.14
1232.35
1197.66
1148.72
1073.63
1046.62
1028.79
991.76
939.21
820.42
768.78
5d
O
O
N
N
N
CH3

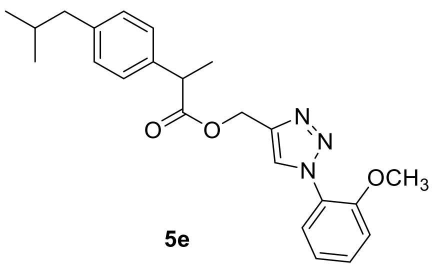
O
O
N
N
N
OCH3

5e

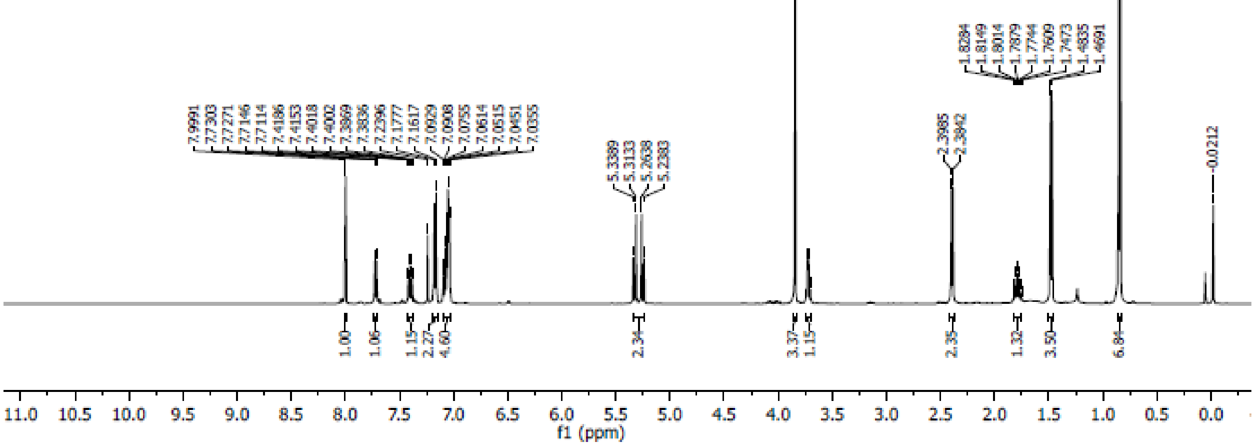
7.9991 7.7303 7.7271 7.7146 7.7114 7.4186 7.4153 7.4018 7.4002 7.3869 7.3836 7.2396 7.1777 7.1617 7.0929 7.0908 7.0755 7.0614 7.0515 7.0451 7.0355
5.3389 5.3133 5.2638 5.2383
3.8433 3.7293 3.7150 3.7007
2.3985 2.3842
1.8284 1.8149 1.8014 1.7879 1.7744 1.7609 1.7473 1.4835 1.4691
0.8525 0.8393
0.0212
1.00 1.06 1.15 2.27 4.60 2.34 3.37 1.15 2.35 1.32 3.50 6.84
f1 (ppm)

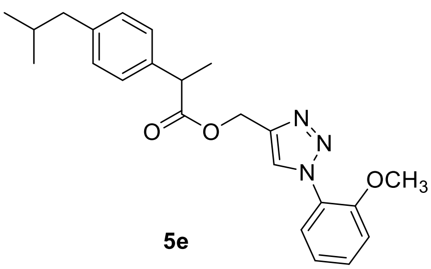
O
O
N
N
N
OCH3

5e

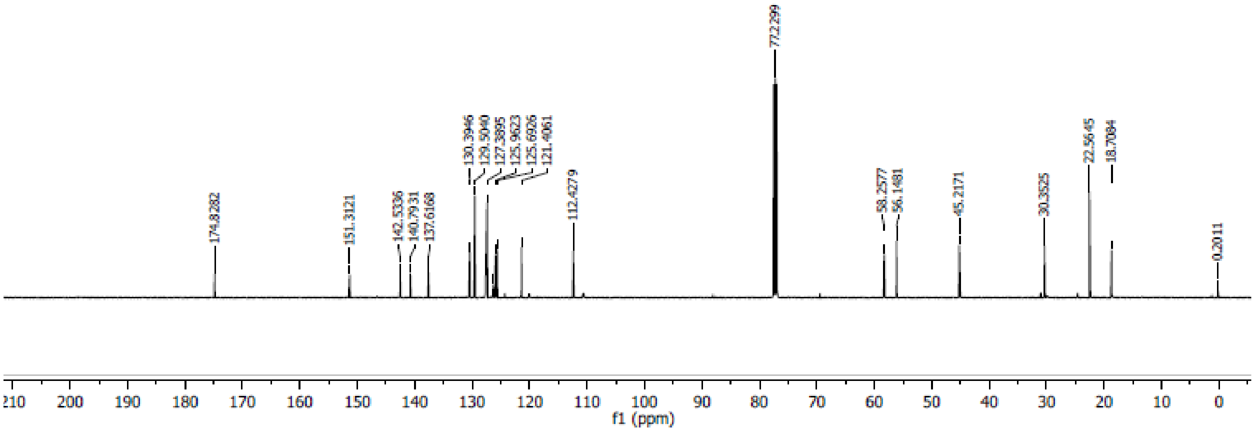
174.8282
151.3121
142.5336 140.7931 137.6168
130.3946 129.5040 127.3895 125.9623 125.6926 121.4061
112.4279
77.2299
58.2577 56.1481
45.2171
30.3525
22.5645 18.7084
0.2011
f1 (ppm)

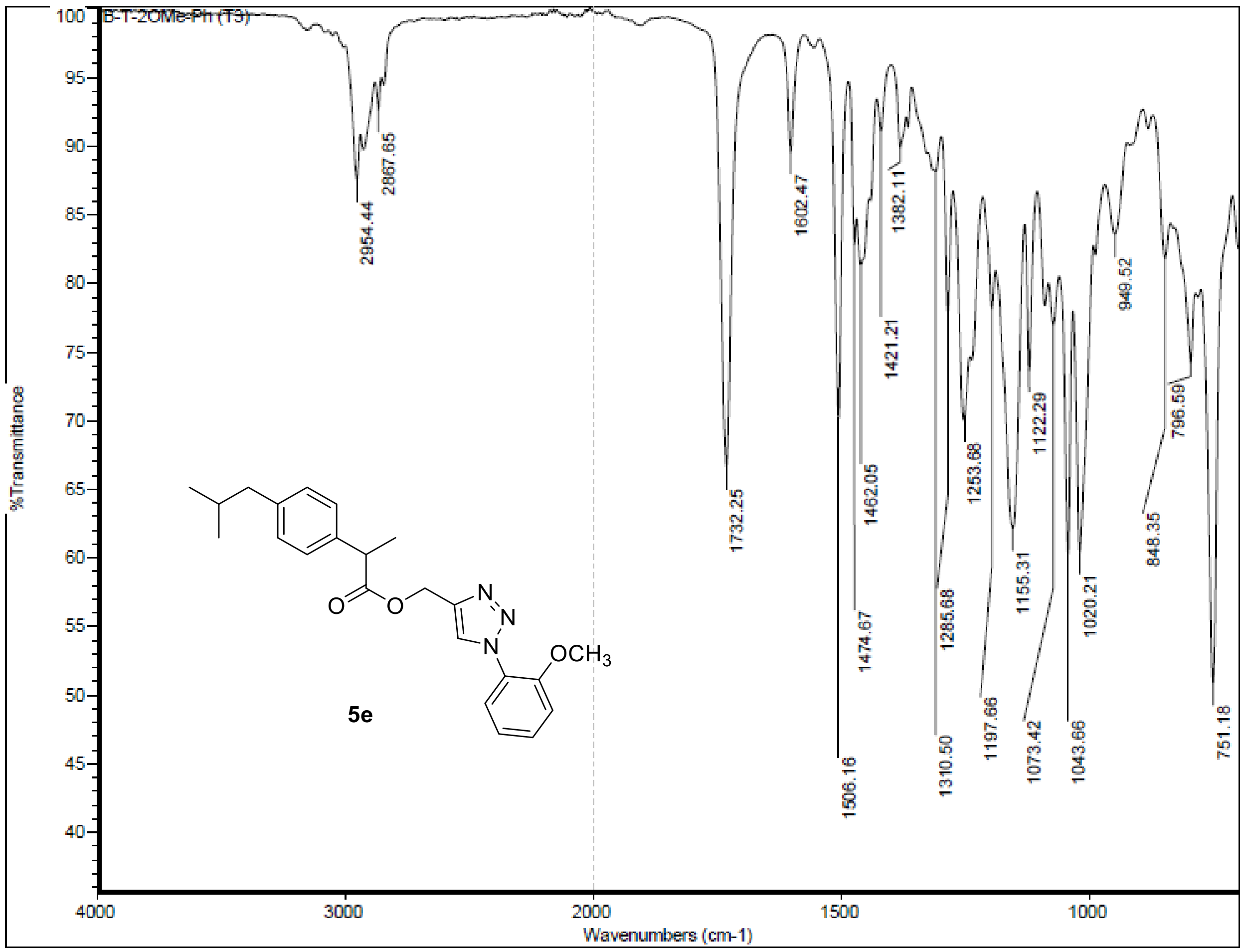
B-T-2OMe-Ph (T3)
%Transmittance
Wavenumbers (cm-1)
2954.44
2867.65
1732.25
1602.47
1506.16
1474.67
1462.05
1421.21
1382.11
1310.50
1285.68
1253.68
1197.66
1155.31
1122.29
1073.42
1043.66
1020.21
949.52
848.35
796.59
751.18
OCH3
N
O
5e

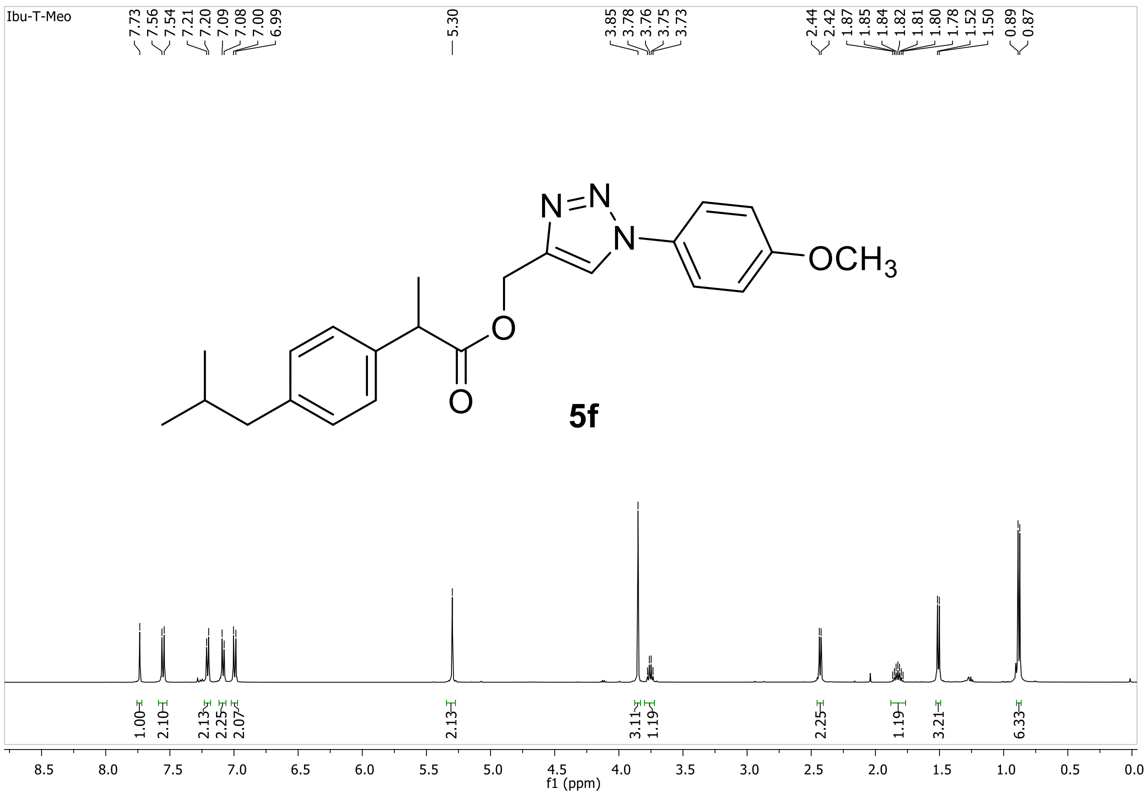
Ibu-T-Meo
7.73
7.56
7.54
7.21
7.20
7.09
7.08
7.00
6.99
5.30
3.85
3.78
3.76
3.75
3.73
2.44
2.42
1.87
1.85
1.84
1.82
1.81
1.80
1.78
1.52
1.50
0.89
0.87
N=N
N
OCH3
O
O
5f
1.00
2.10
2.13
2.25
2.07
2.13
3.11
1.19
2.25
1.19
3.21
6.33
8.5 8.0 7.5 7.0 6.5 6.0 5.5 5.0 4.5 4.0 3.5 3.0 2.5 2.0 1.5 1.0 0.5 0.0
f1 (ppm)

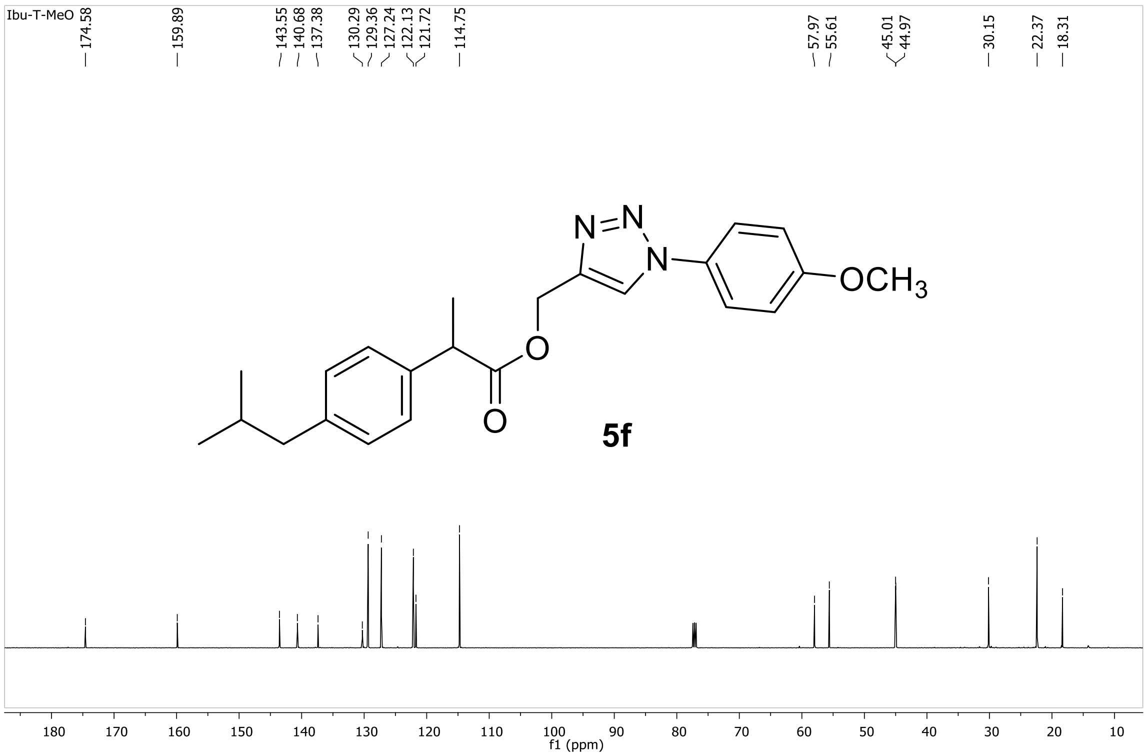
Ibu-T-MeO
174.58
159.89
143.55
140.68
137.38
130.29
129.36
127.24
122.13
121.72
114.75
57.97
55.61
45.01
44.97
30.15
22.37
18.31
N=N
N
OCH3
O
O
5f
180 170 160 150 140 130 120 110 100 90 80 70 60 50 40 30 20 10
f1 (ppm)

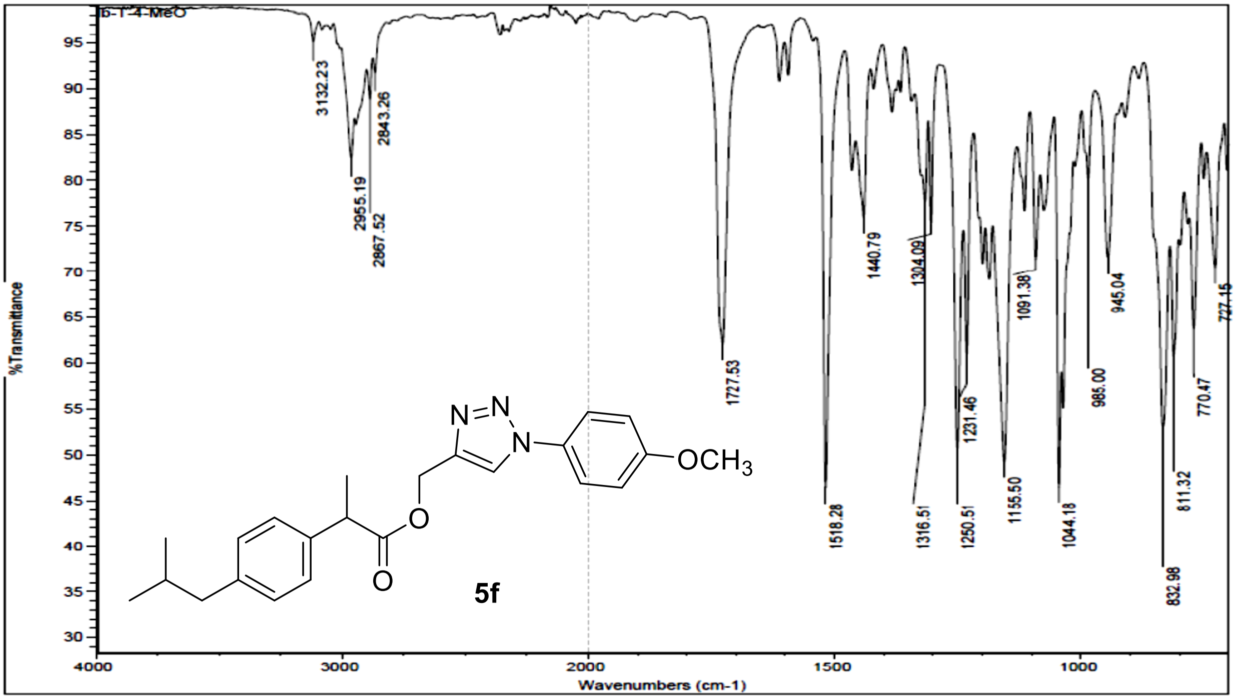

b-T-4-MeO
%Transmittance
Wavenumbers (cm-1)
3132.23
2955.19
2867.52
2843.26
1727.53
1518.28
1440.79
1316.51
1304.09
1250.51
1231.46
1155.50
1091.38
1044.18
985.00
945.04
832.98
811.32
770.47
727.15
OCH3
5f

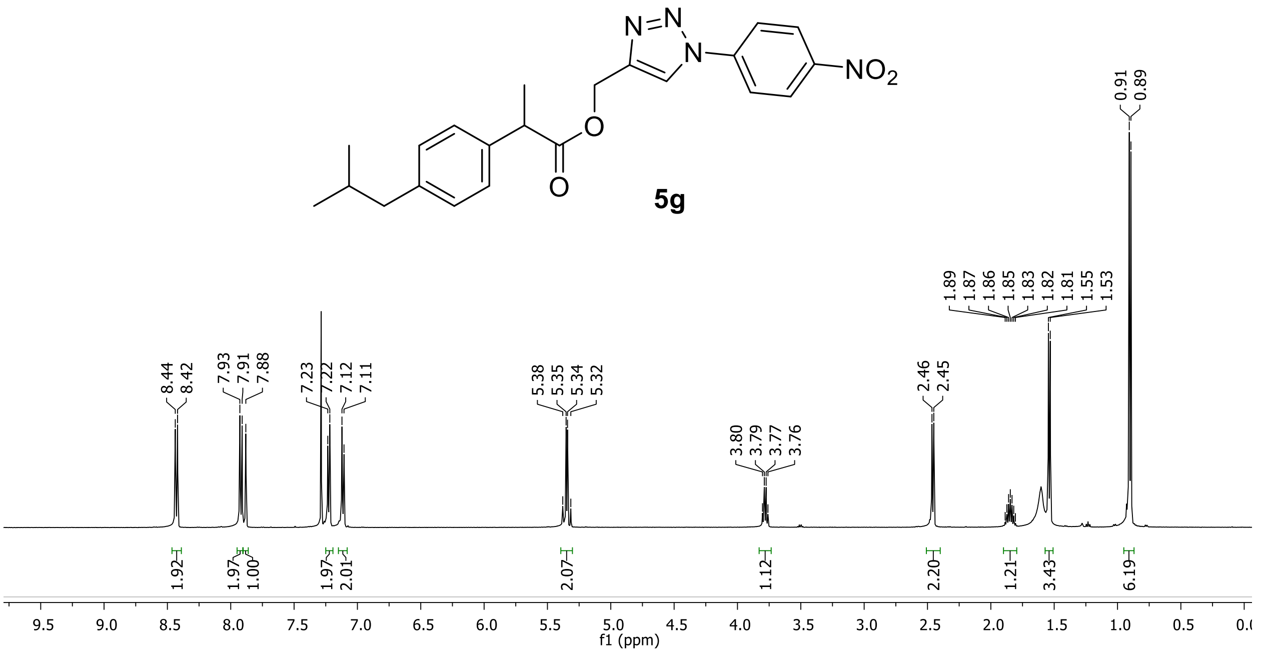

5g
NO2
8.44
8.42
7.93
7.91
7.88
7.23
7.22
7.12
7.11
5.38
5.35
5.34
5.32
3.80
3.79
3.77
3.76
2.46
2.45
1.89
1.87
1.86
1.85
1.83
1.82
1.81
1.55
1.53
0.91
0.89
1.92
1.97
1.00
1.97
2.01
2.07
1.12
2.20
1.21
3.43
6.19
f1 (ppm)

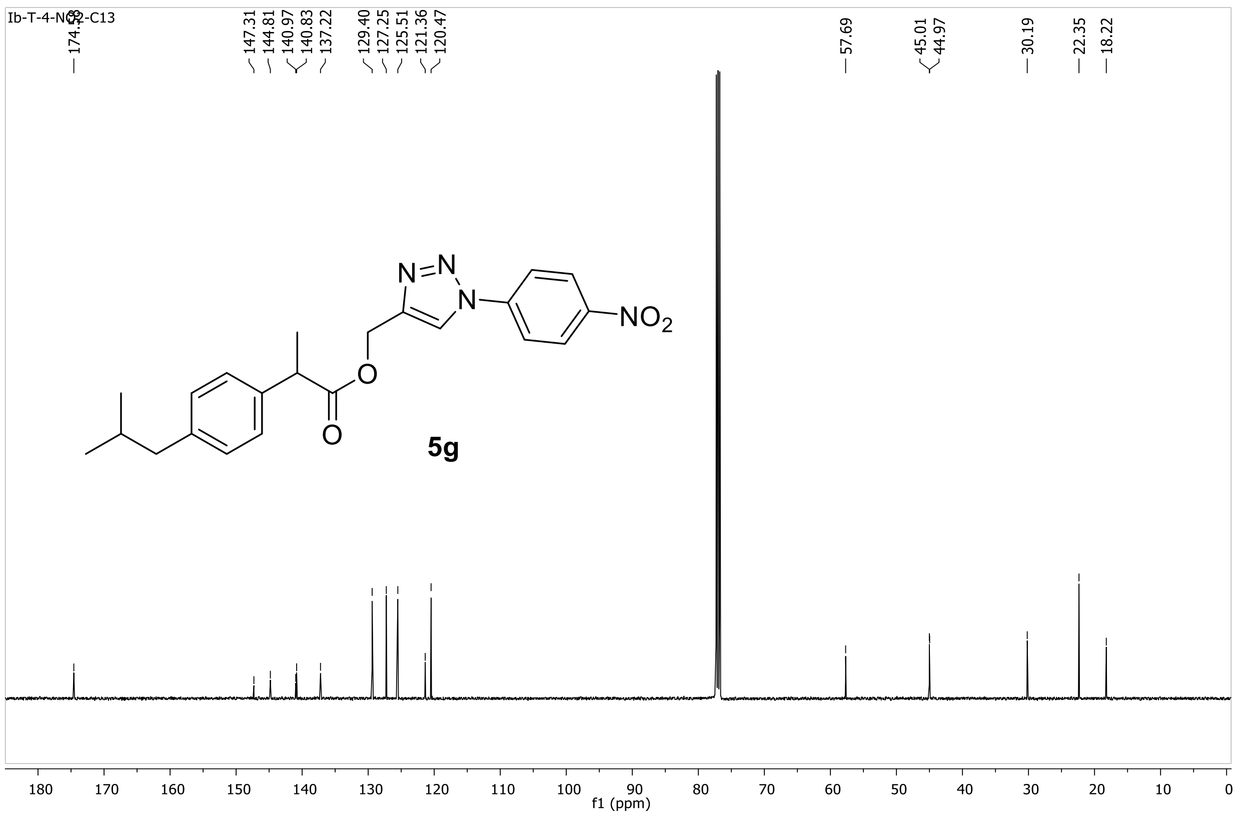

174.48
147.31
144.81
140.97
140.83
137.22
129.40
127.25
125.51
121.36
120.47
57.69
45.01
44.97
30.19
22.35
18.22
5g
NO2
f1 (ppm)

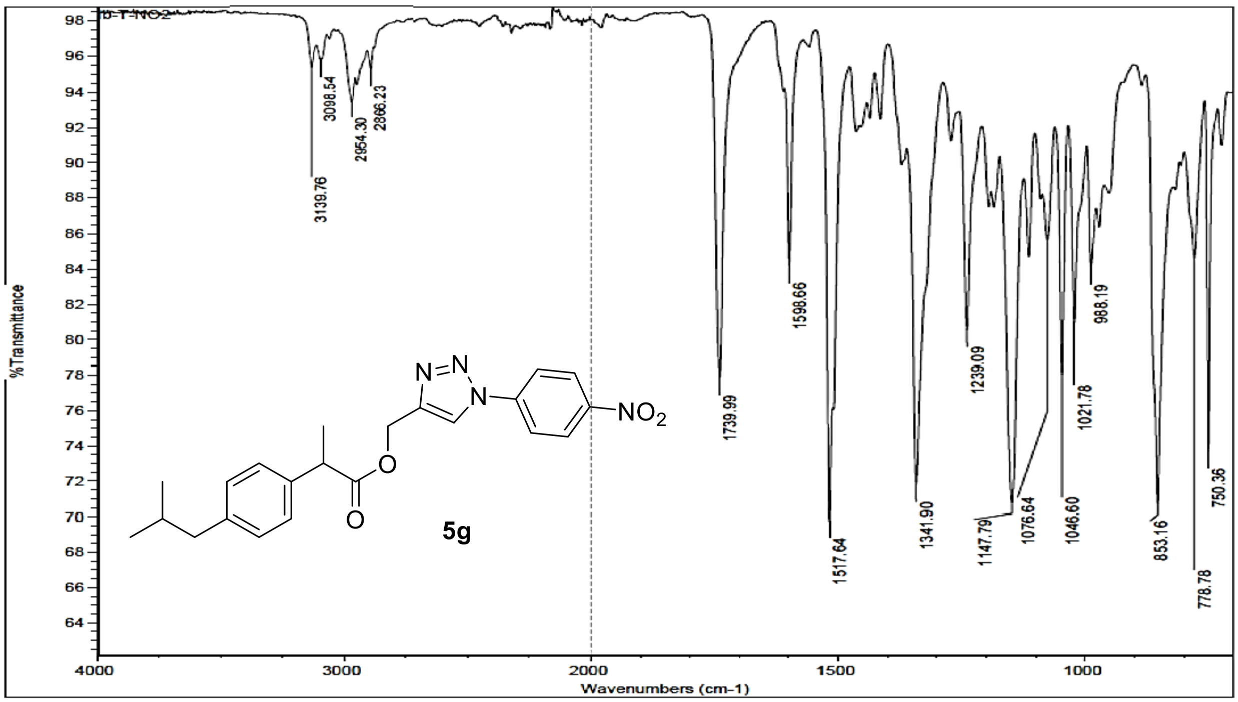

%Transmittance
Wavenumbers (cm-1)
3139.76
3098.54
2954.30
2866.23
1739.99
1598.66
1517.64
1341.90
1239.09
1147.79
1076.64
1046.60
1021.78
988.19
853.16
778.78
750.36
5g

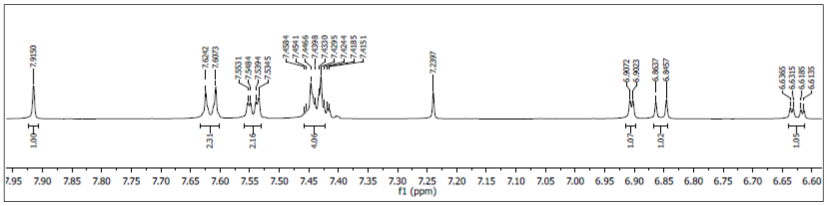
7.9150
7.6242
7.6073
7.5531
7.5484
7.5394
7.5345
7.4584
7.4541
7.4466
7.4398
7.4330
7.4295
7.4244
7.4185
7.4151
7.2397
6.9072
6.9023
6.8637
6.8457
6.6365
6.6315
6.6185
6.6135
1.00
2.31
2.16
4.06
1.07
1.02
1.05
f1 (ppm)

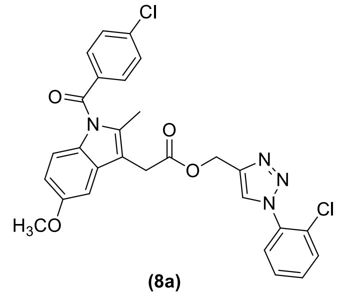
Cl
O
N
O
O
N
N
N
H3CO
Cl

(8a)

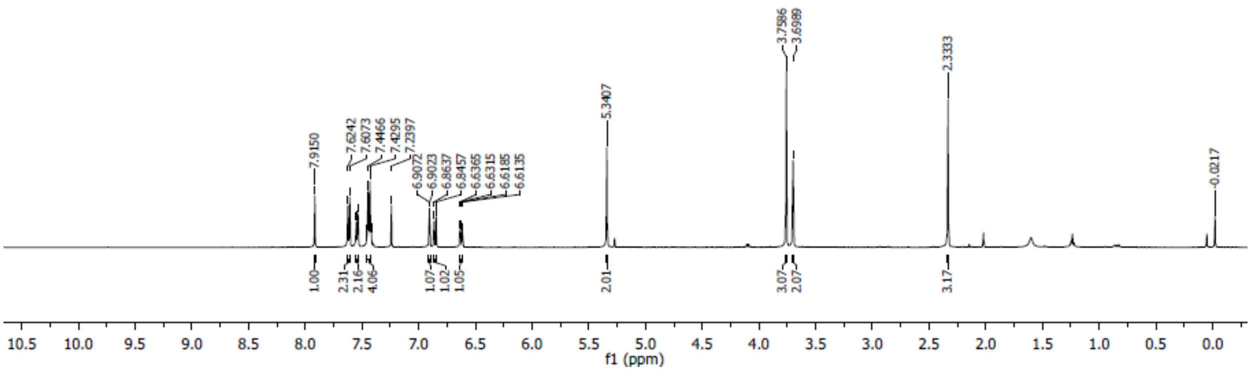
7.9150
7.6242
7.6073
7.4466
7.4295
7.2397
6.9072
6.9023
6.8637
6.8457
6.6365
6.6315
6.6185
6.6135
5.3407
3.7586
3.6989
2.3333
-0.0217
1.00
2.31
2.16
4.06
1.07
1.02
1.05
2.01
3.07
2.07
3.17
f1 (ppm)

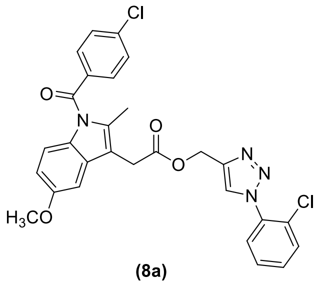
Cl
O
N
O
O
N
N
N
H3CO
Cl

(8a)

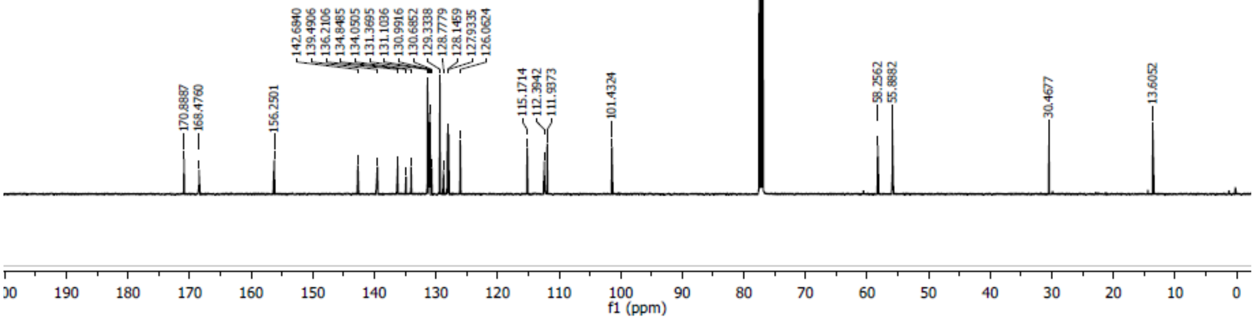
170.8887
168.4760
156.2501
142.6840
139.4906
136.2106
134.8485
134.0505
131.3695
131.1036
130.9916
130.6852
129.3338
128.7779
128.1459
127.9335
126.0624
115.1714
112.3942
111.9373
101.4324
77.2300
58.2562
55.8882
30.4677
13.6052
f1 (ppm)

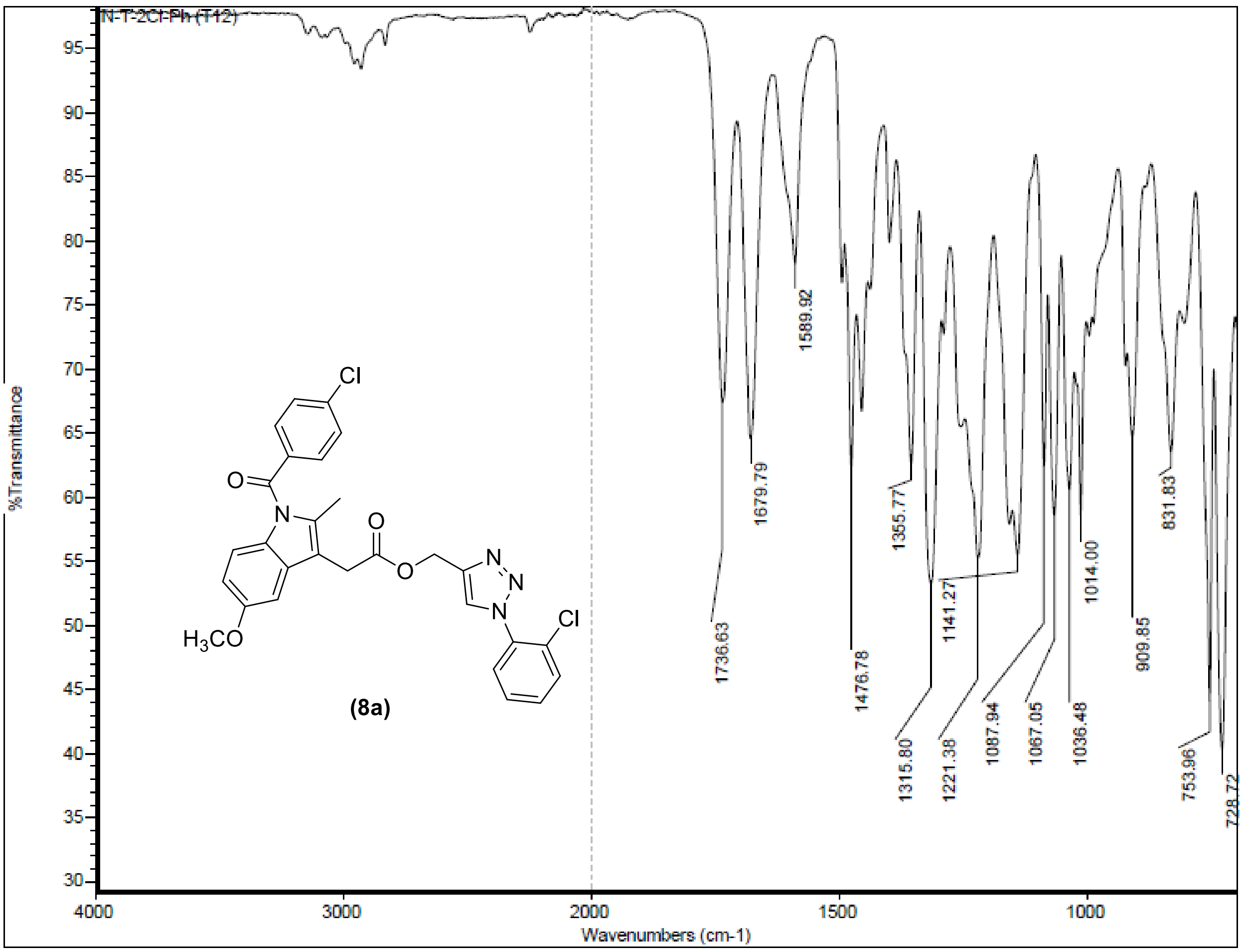
N-T-2Cl-Ph (T12)
%Transmittance
Wavenumbers (cm-1)
1736.63
1679.79
1589.92
1476.78
1355.77
1315.80
1221.38
1141.27
1087.94
1067.05
1036.48
1014.00
909.85
831.83
753.96
728.72
(8a)

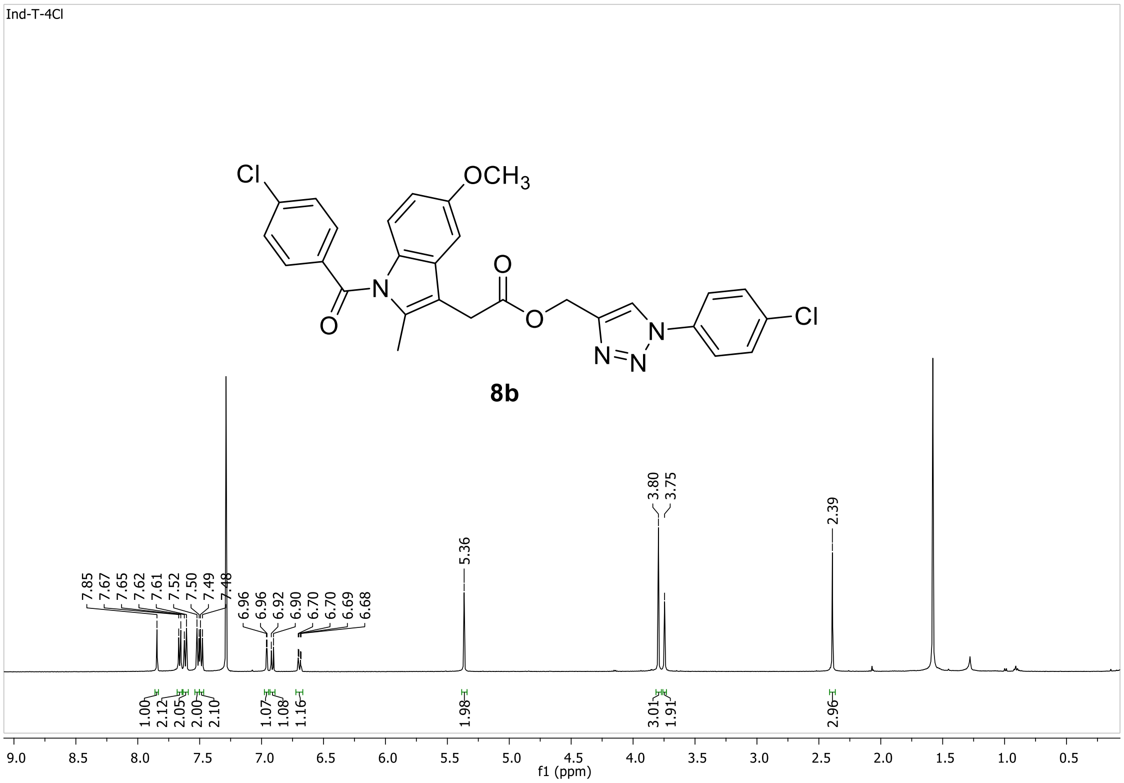

Ind-T-4Cl
Cl
OCH3
O
N
O
O
N
N=N
Cl
8b
7.85
7.67
7.65
7.62
7.61
7.52
7.50
7.49
7.48
6.96
6.96
6.92
6.90
6.70
6.70
6.69
6.68
5.36
3.80
3.75
2.39
1.00
2.12
2.05
2.00
2.10
1.07
1.08
1.16
1.98
3.01
1.91
2.96
9.0 8.5 8.0 7.5 7.0 6.5 6.0 5.5 5.0 4.5 4.0 3.5 3.0 2.5 2.0 1.5 1.0 0.5
f1 (ppm)

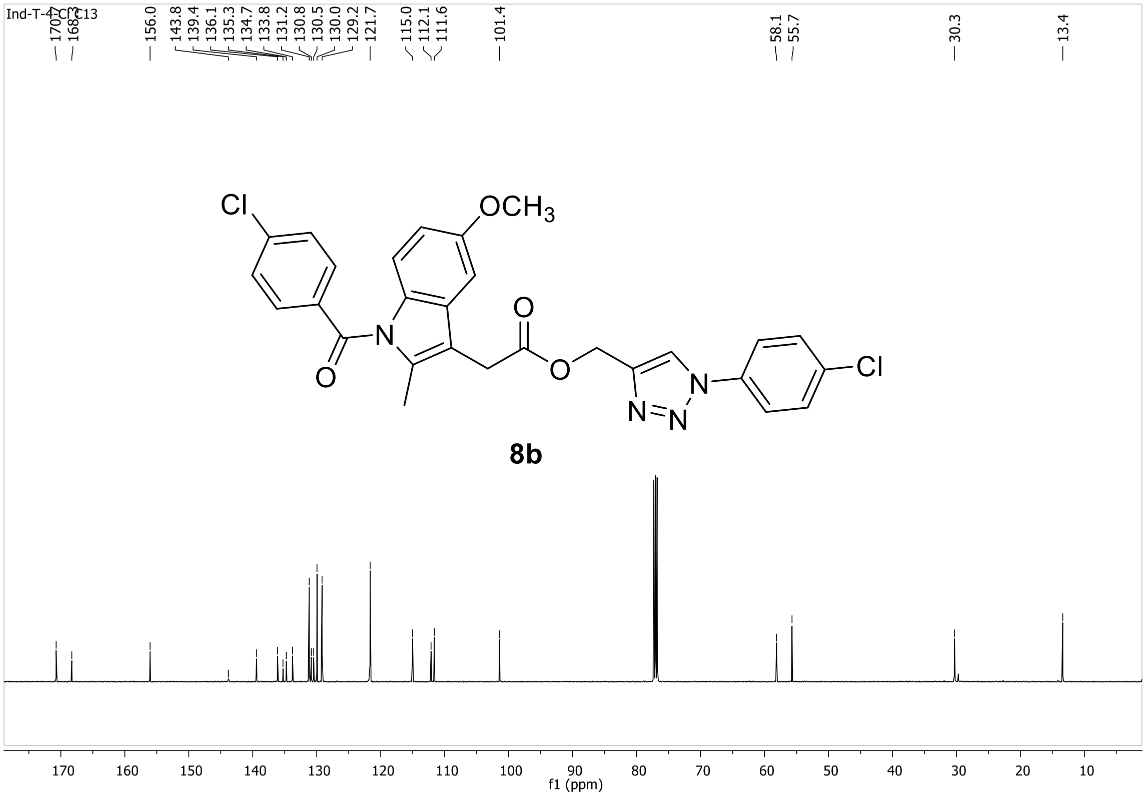

Ind-T-4-Cl C13
156.0
143.8
139.4
136.1
135.3
134.7
133.8
131.2
130.8
130.5
130.0
129.2
121.7
115.0
112.1
111.6
101.4
58.1
55.7
30.3
13.4
Cl
OCH3
O
N
O
O
N
N=N
Cl
8b
170 160 150 140 130 120 110 100 90 80 70 60 50 40 30 20 10
f1 (ppm)

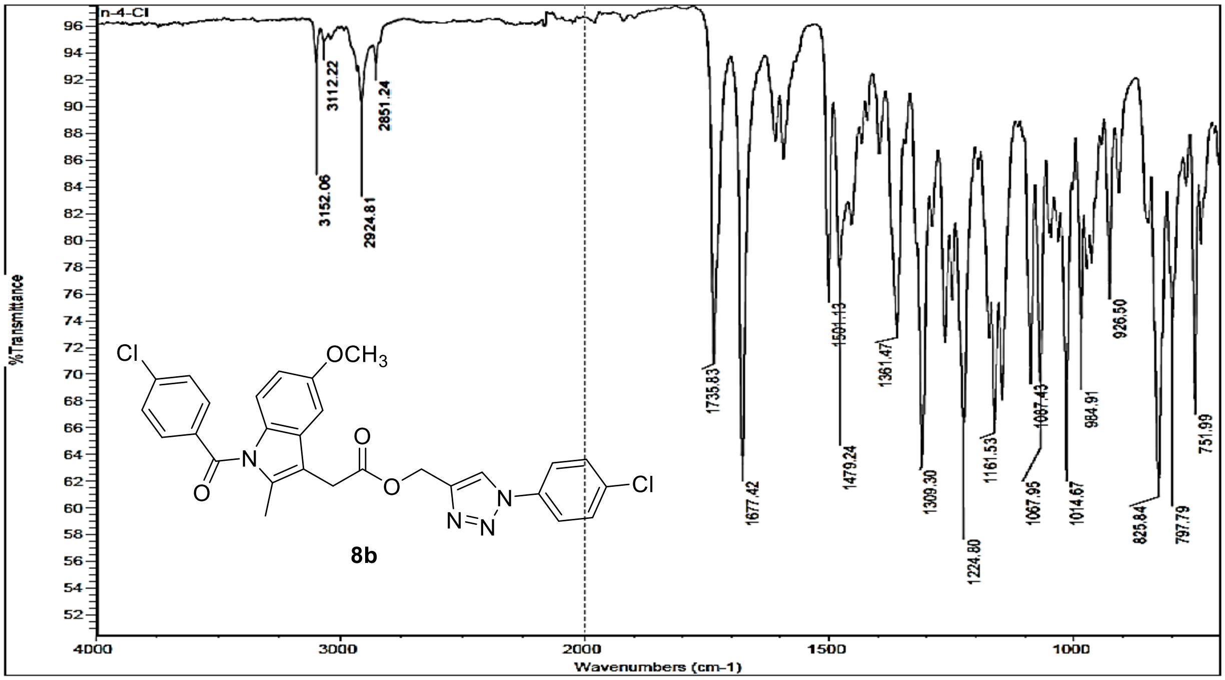

n-4-Cl
%Transmittance
Wavenumbers (cm-1)
3152.06
3112.22
2924.81
2851.24
1735.83
1677.42
1501.13
1479.24
1361.47
1309.30
1224.80
1161.53
1087.43
1067.95
1014.67
984.91
926.50
825.84
797.79
751.99
Cl
OCH3
N
O
N
N
N
8b

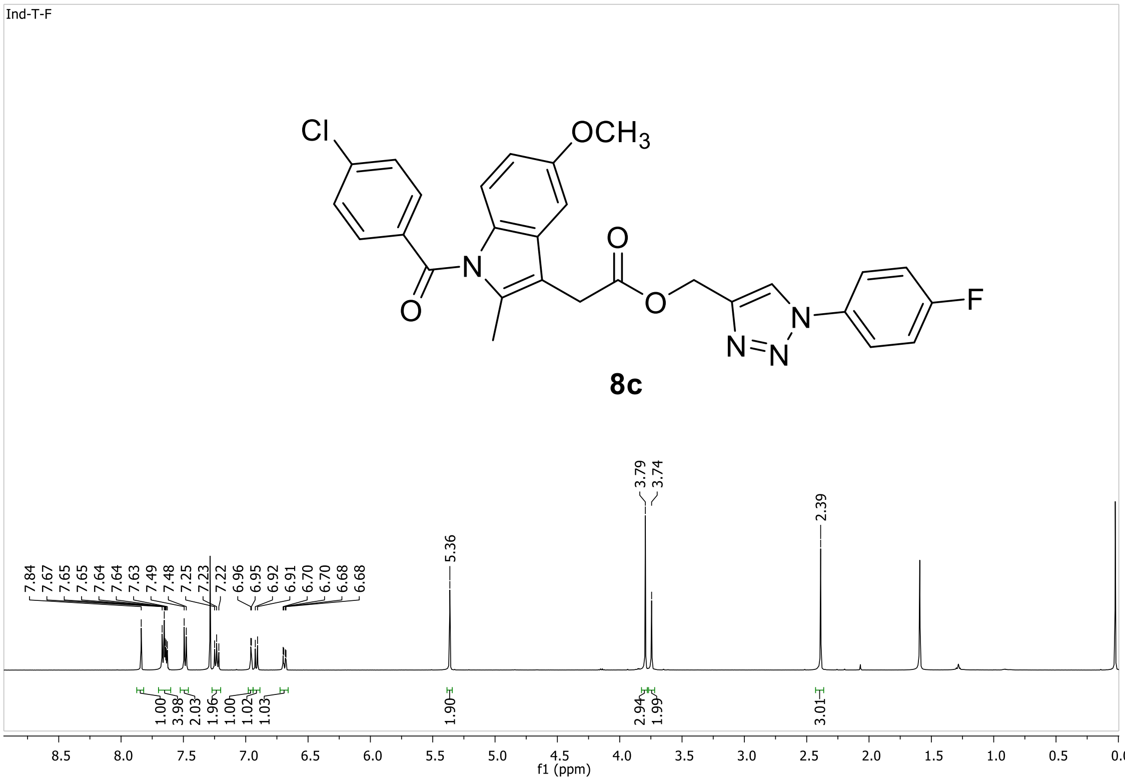
Ind-T-F
Cl
OCH3
O
N
O
N
N=N
F
8c
7.84 7.67 7.65 7.65 7.64 7.64 7.63 7.49 7.48 7.25 7.23 7.22 6.96 6.95 6.92 6.91 6.70 6.70 6.68 6.68
5.36
3.79 3.74
2.39
1.00 3.98 2.03 1.96 1.00 1.02 1.03
1.90
2.94 1.99
3.01
8.5 8.0 7.5 7.0 6.5 6.0 5.5 5.0 4.5 4.0 3.5 3.0 2.5 2.0 1.5 1.0 0.5 0.0
f1 (ppm)

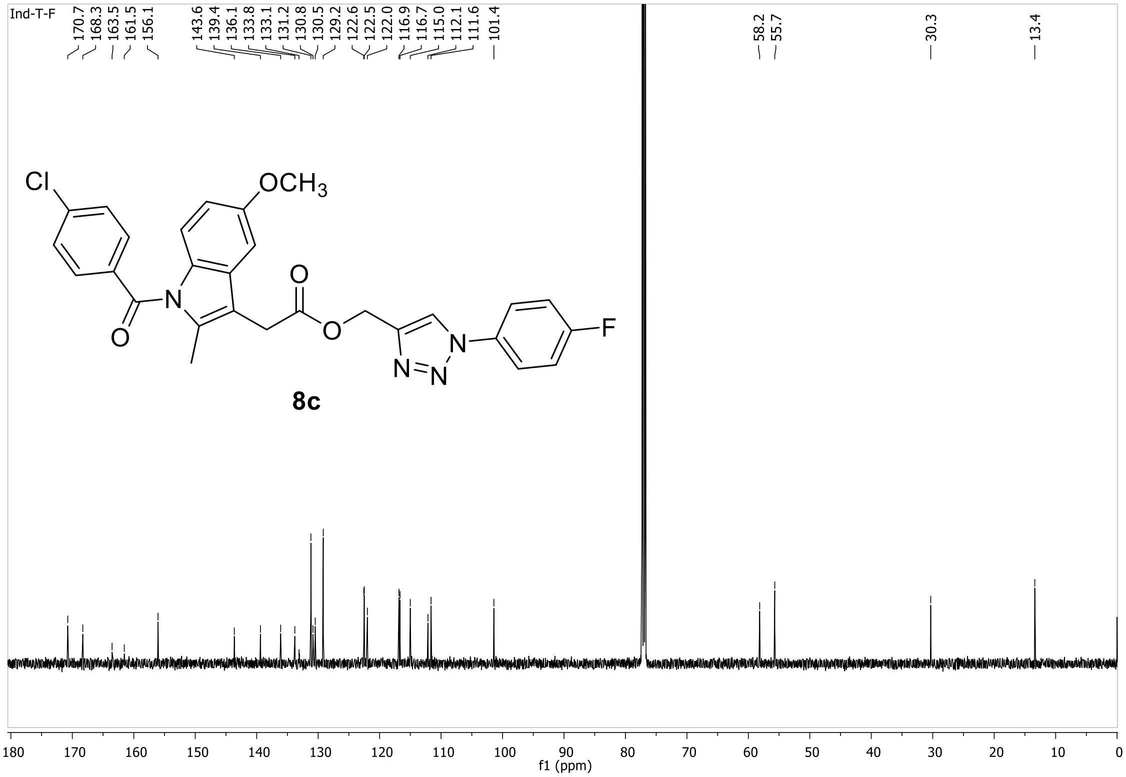
Ind-T-F
170.7 168.3 163.5 161.5 156.1
143.6 139.4 136.1 133.8 133.1 131.2 130.8 130.5 129.2 122.6 122.5 122.0 116.9 116.7 115.0 112.1 111.6 101.4
58.2 55.7
30.3
13.4
Cl
OCH3
O
N
O
N
N=N
F
8c
180 170 160 150 140 130 120 110 100 90 80 70 60 50 40 30 20 10 0
f1 (ppm)

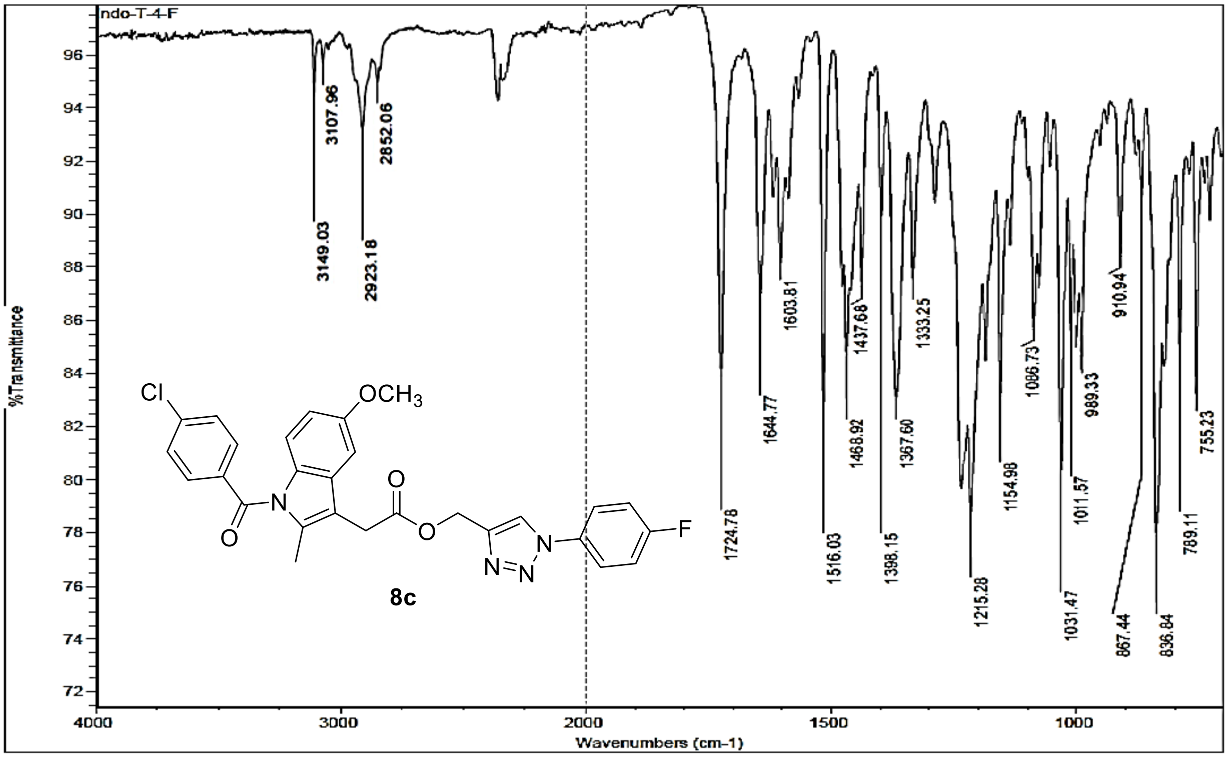
ndo-T-4-F
%Transmittance
Wavenumbers (cm-1)
4000
3000
2000
1500
1000
96
94
92
90
88
86
84
82
80
78
76
74
72
3149.03
3107.96
2923.18
2852.06
1724.78
1644.77
1603.81
1516.03
1468.92
1437.68
1398.15
1367.60
1333.25
1215.28
1154.98
1086.73
1031.47
1011.57
989.33
910.94
867.44
836.84
789.11
755.23
Cl
OCH_3
N
O
O
O
N
N
N
F
8c

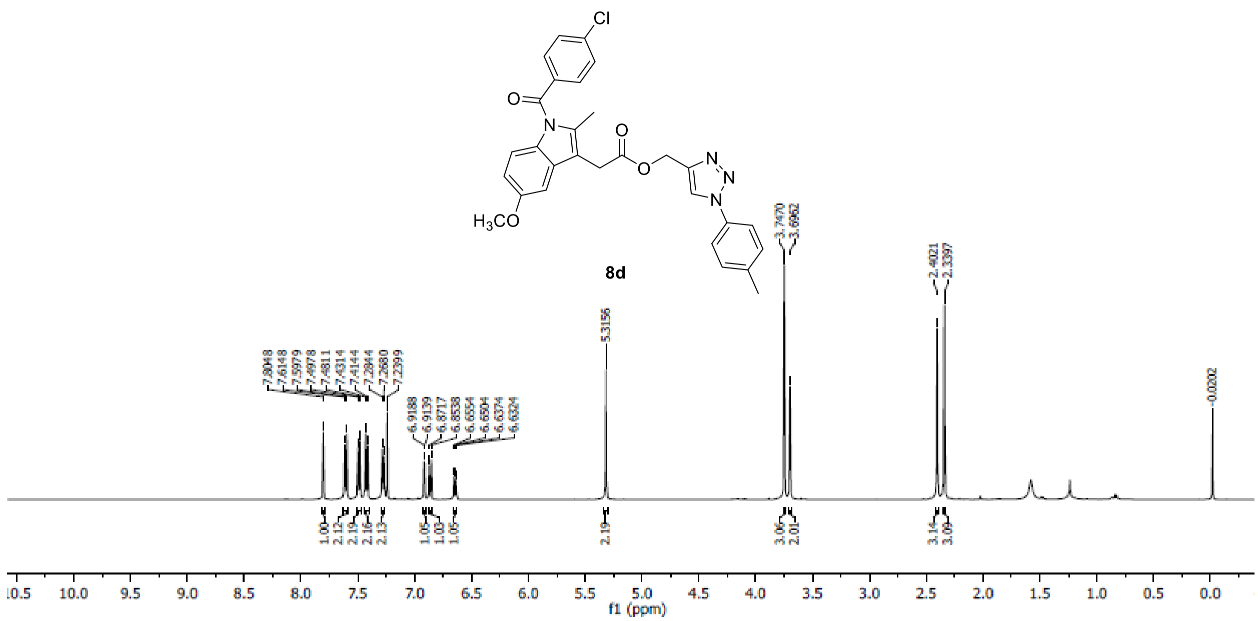
8d
f1 (ppm)

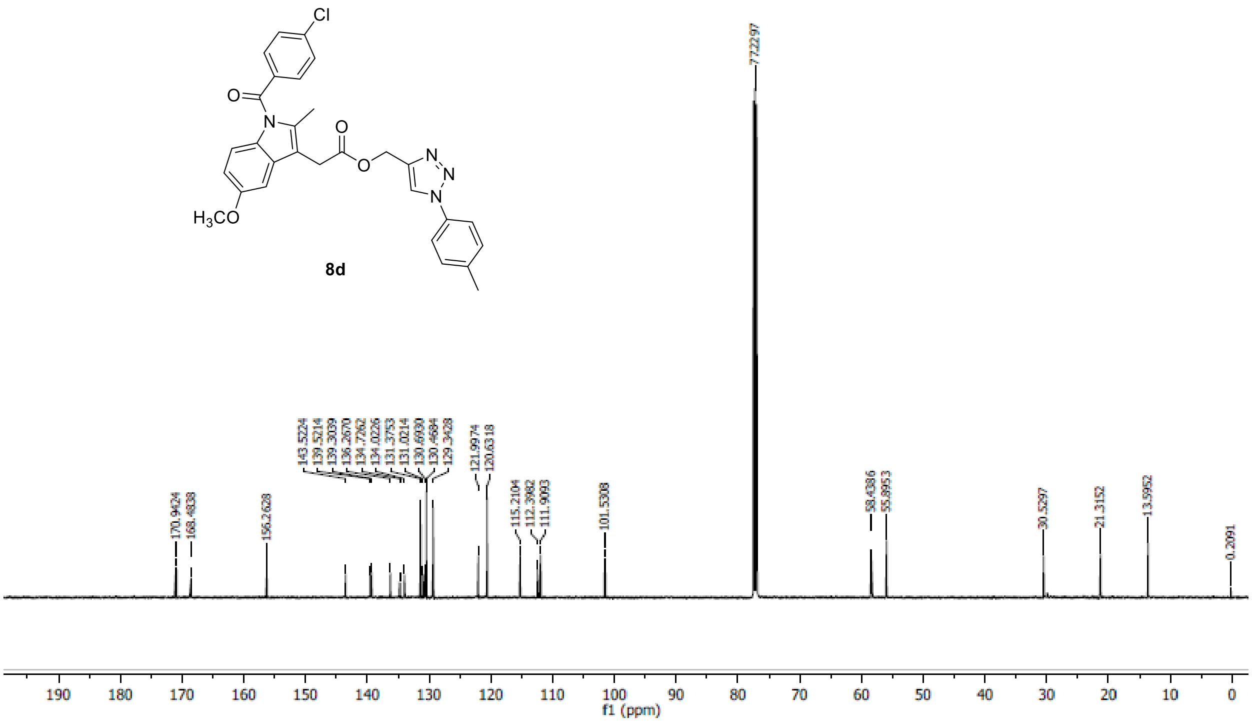
8d
f1 (ppm)

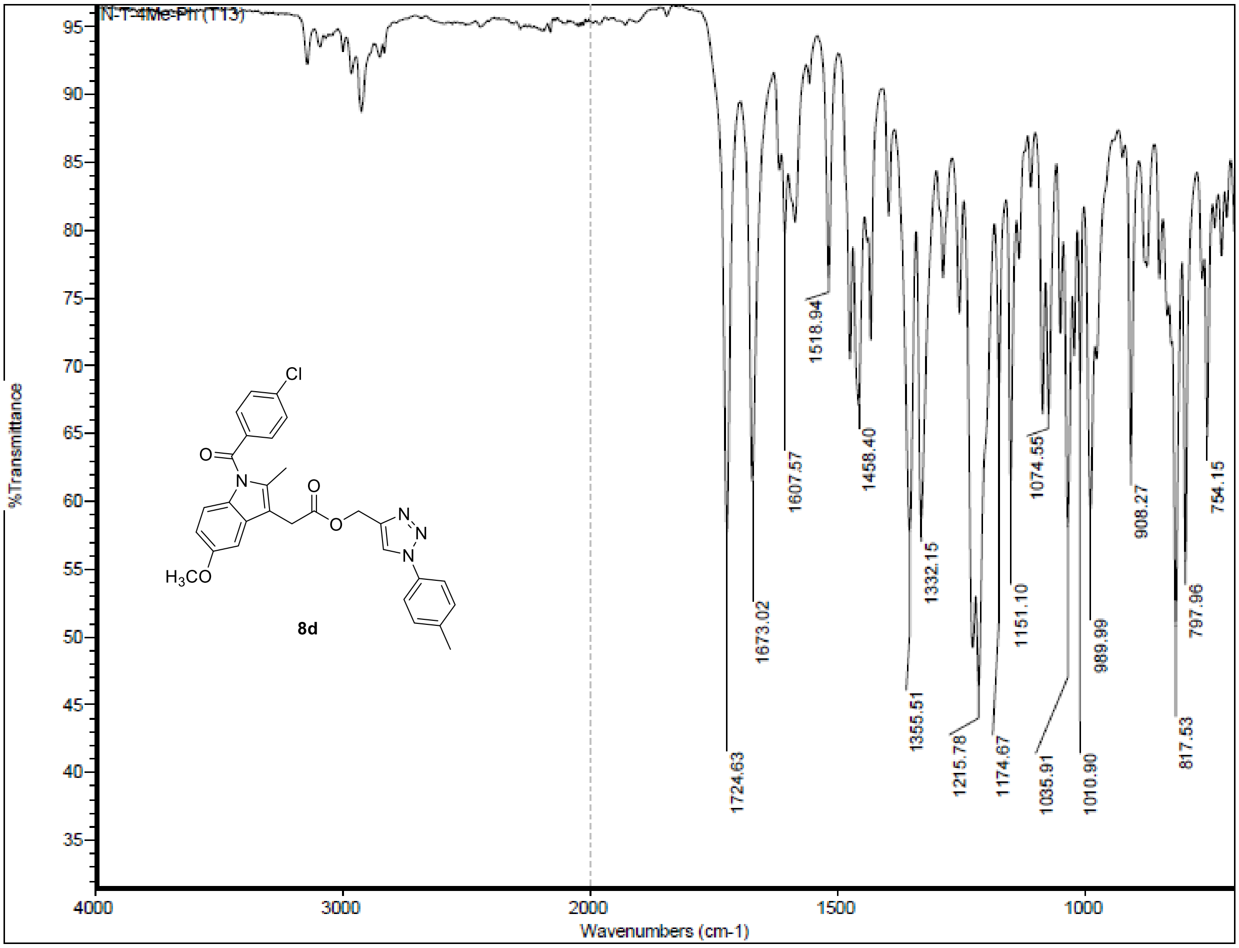
N-T-4Me-Ph (T13)
%Transmittance
Wavenumbers (cm-1)
1724.63
1673.02
1607.57
1518.94
1458.40
1355.51
1332.15
1215.78
1174.67
1151.10
1074.55
1035.91
1010.90
989.99
908.27
817.53
797.96
754.15
Cl
O
N
O
O
N
N
N
H_3CO
8d

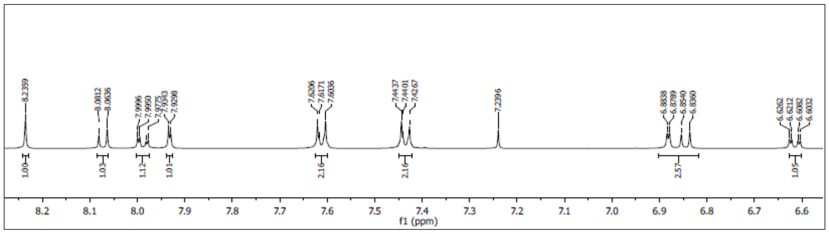

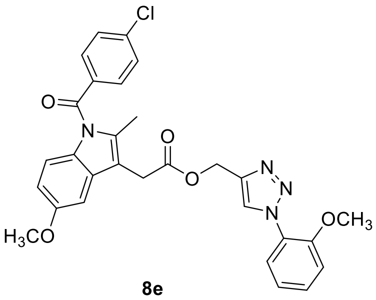

8e

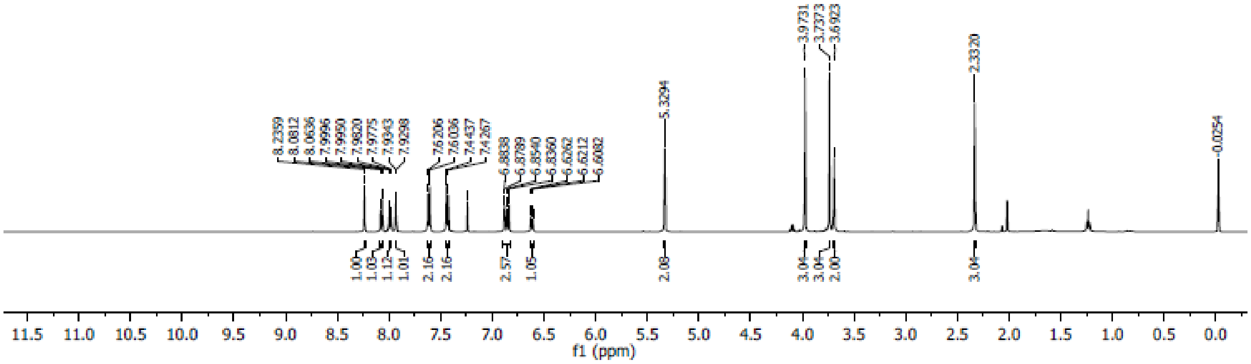

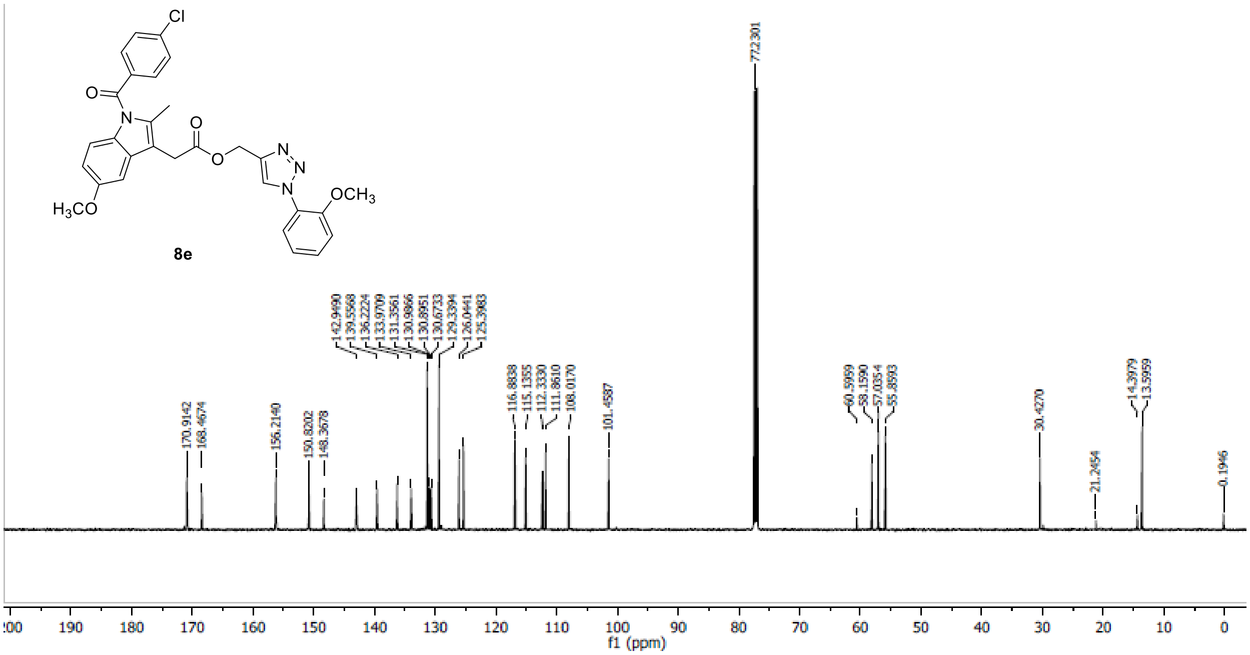

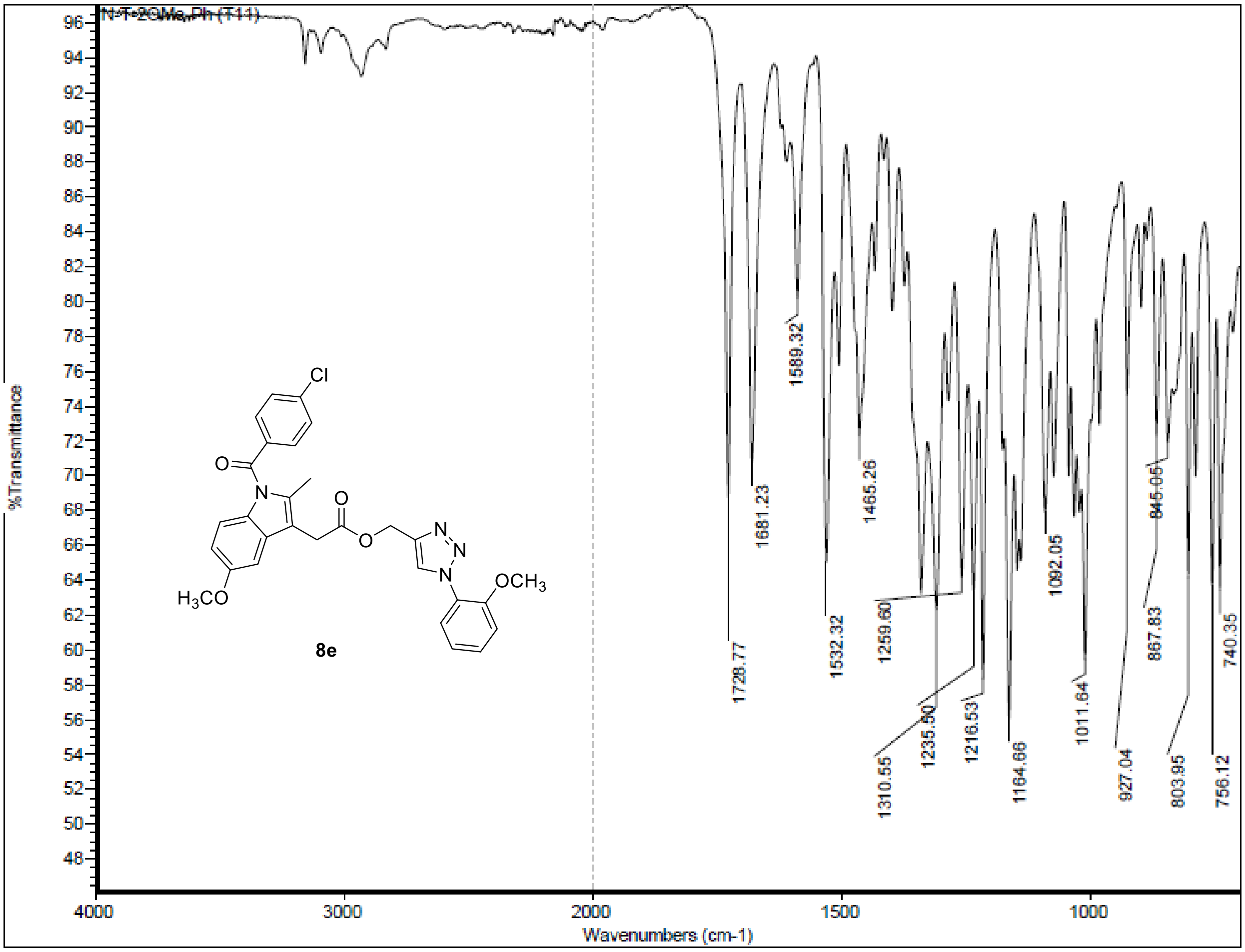

N-T-2OMe-Ph (T11)
%Transmittance
Wavenumbers (cm-1)
O
N
O
O
Cl
H3CO
N
N
N
OCH3
8e
1728.77
1681.23
1589.32
1532.32
1465.26
1310.55
1259.60
1235.50
1216.53
1164.66
1092.05
1011.64
927.04
867.83
845.05
803.95
756.12
740.35

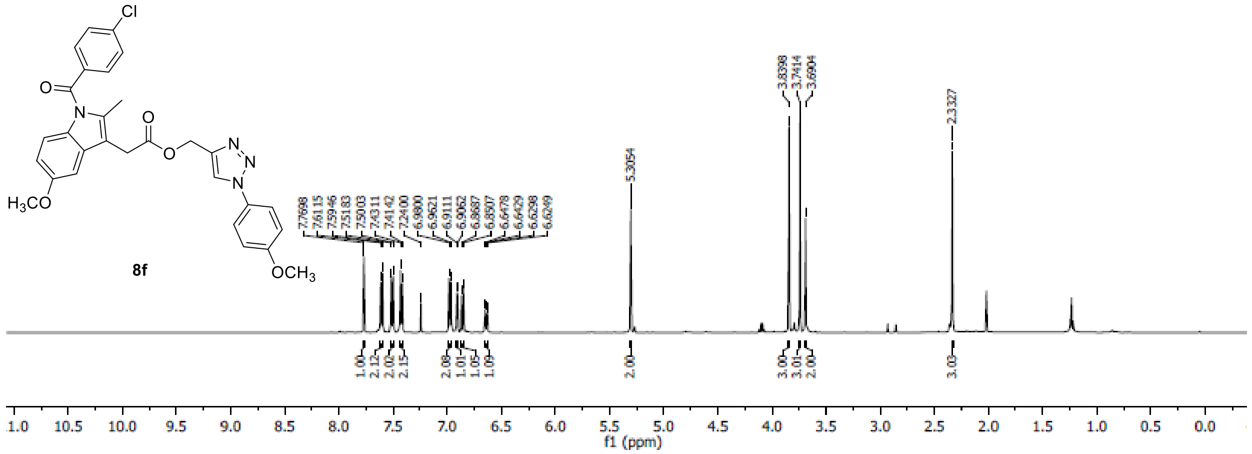
Cl
O
N
O
O
N
N
N
H3CO
OCH3
8f
7.7698 7.6115 7.5946 7.5183 7.5003 7.4311 7.4142 7.2400 6.9800 6.9621 6.9111 6.9062 6.8687 6.8507 6.6478 6.6429 6.6298 6.6249
5.3054
3.8398 3.7414 3.6904
2.3327
1.00 2.12 2.02 2.15 2.08 1.01 1.05 1.09
2.00
3.00 3.01 2.00
3.03
f1 (ppm)

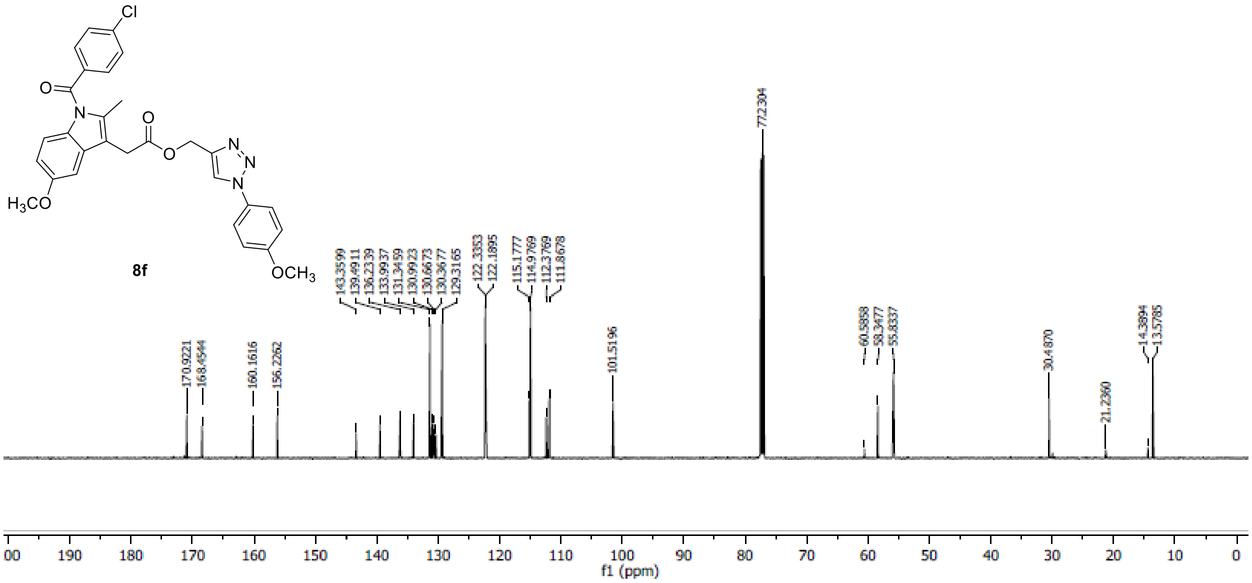
Cl
O
N
O
O
N
N
N
H3CO
OCH3
8f
170.9221 168.4544 160.1616 156.2262
143.3599 139.4911 136.2339 133.9937 131.3459 130.9923 130.6673 130.3677 129.3165
122.3353 122.1895
115.1777 114.9769 112.3769 111.8678
101.5196
77.2304
60.5858 58.3477 55.8337
30.4870
21.2360
14.3894 13.5785
f1 (ppm)

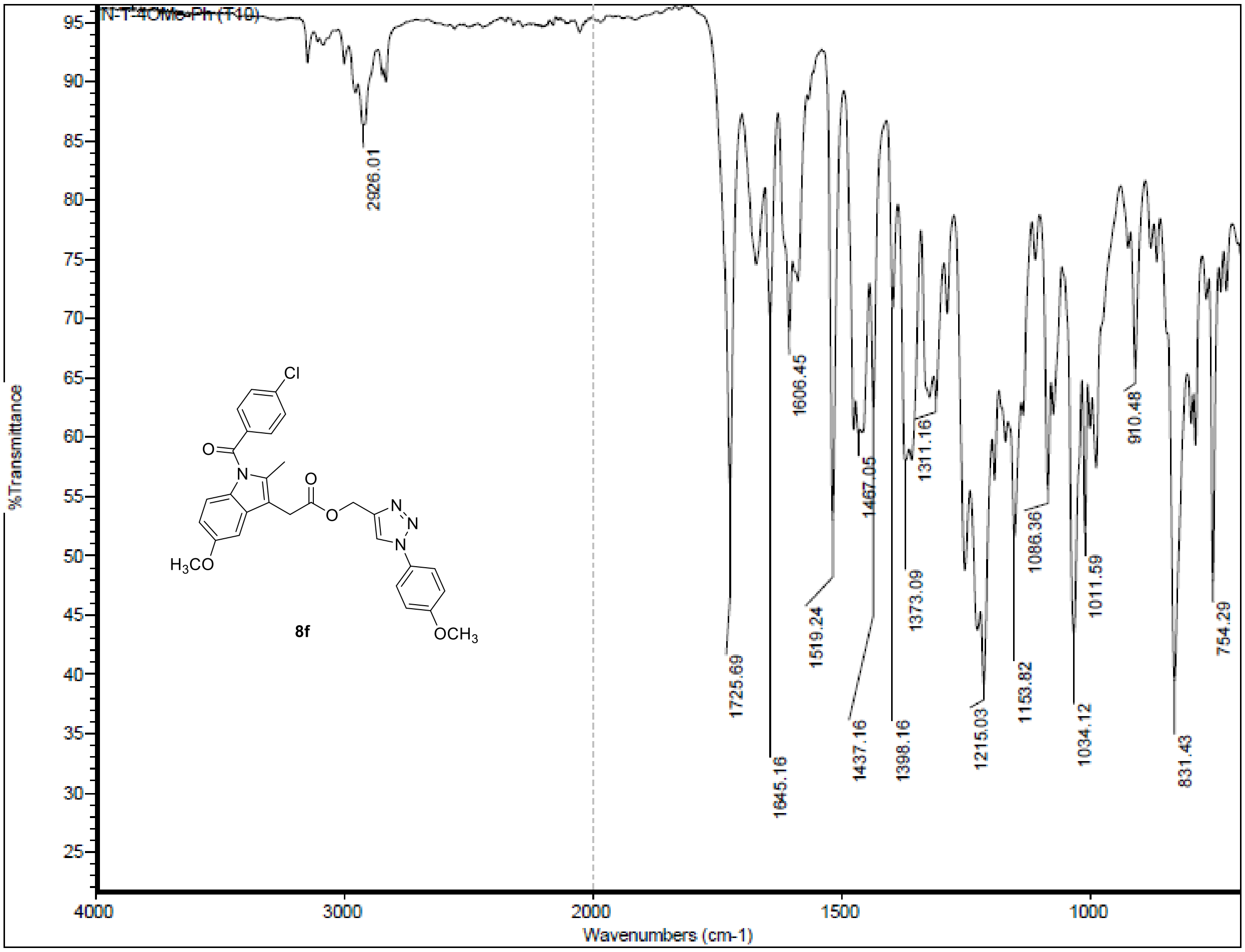
N-T-4OMe-Ph (T10)
%Transmittance
Wavenumbers (cm-1)
2926.01
1725.69
1645.16
1606.45
1519.24
1467.05
1437.16
1398.16
1373.09
1311.16
1215.03
1153.82
1086.36
1034.12
1011.59
910.48
831.43
754.29
8f

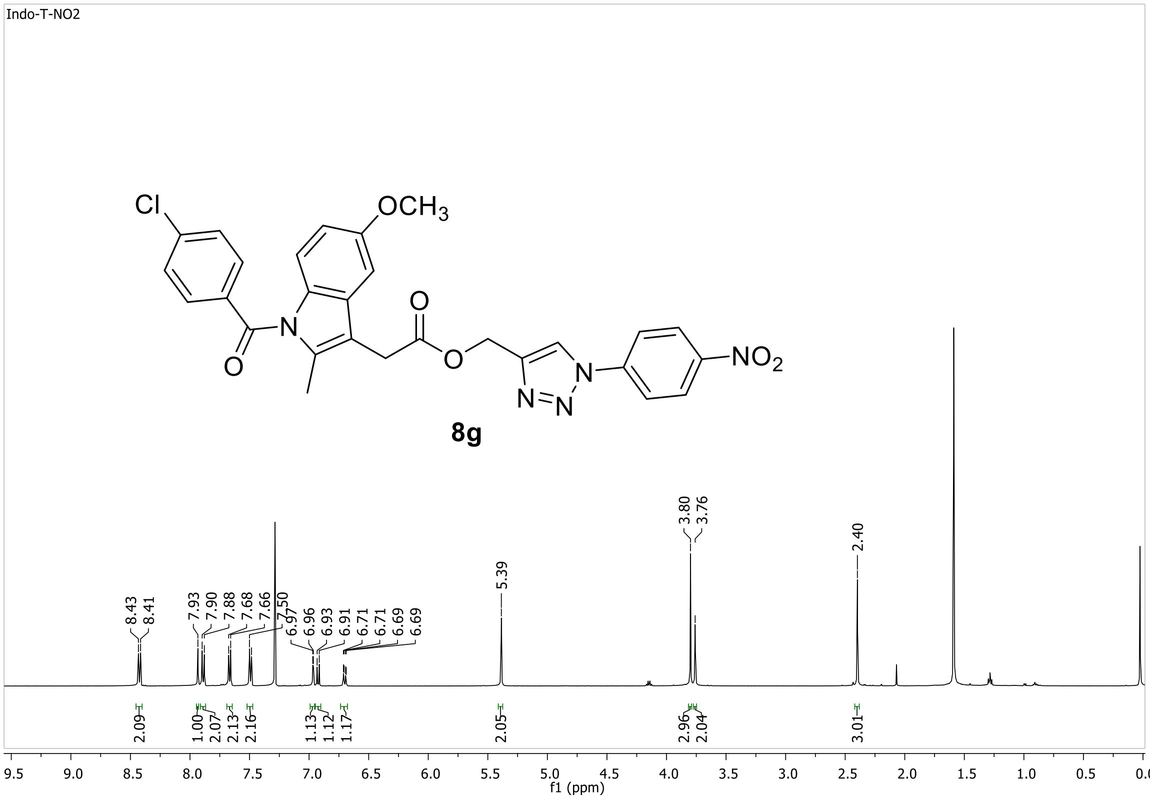
Indo-T-NO2
Cl
OCH3
O
N
O
O
N
N=N
NO2
8g
8.43
8.41
7.93
7.90
7.88
7.68
7.66
7.50
6.97
6.96
6.93
6.91
6.71
6.71
6.69
6.69
5.39
3.80
3.76
2.40
2.09
1.00
2.07
2.13
2.16
1.13
1.12
1.17
2.05
2.96
2.04
3.01
9.5 9.0 8.5 8.0 7.5 7.0 6.5 6.0 5.5 5.0 4.5 4.0 3.5 3.0 2.5 2.0 1.5 1.0 0.5 0.0
f1 (ppm)

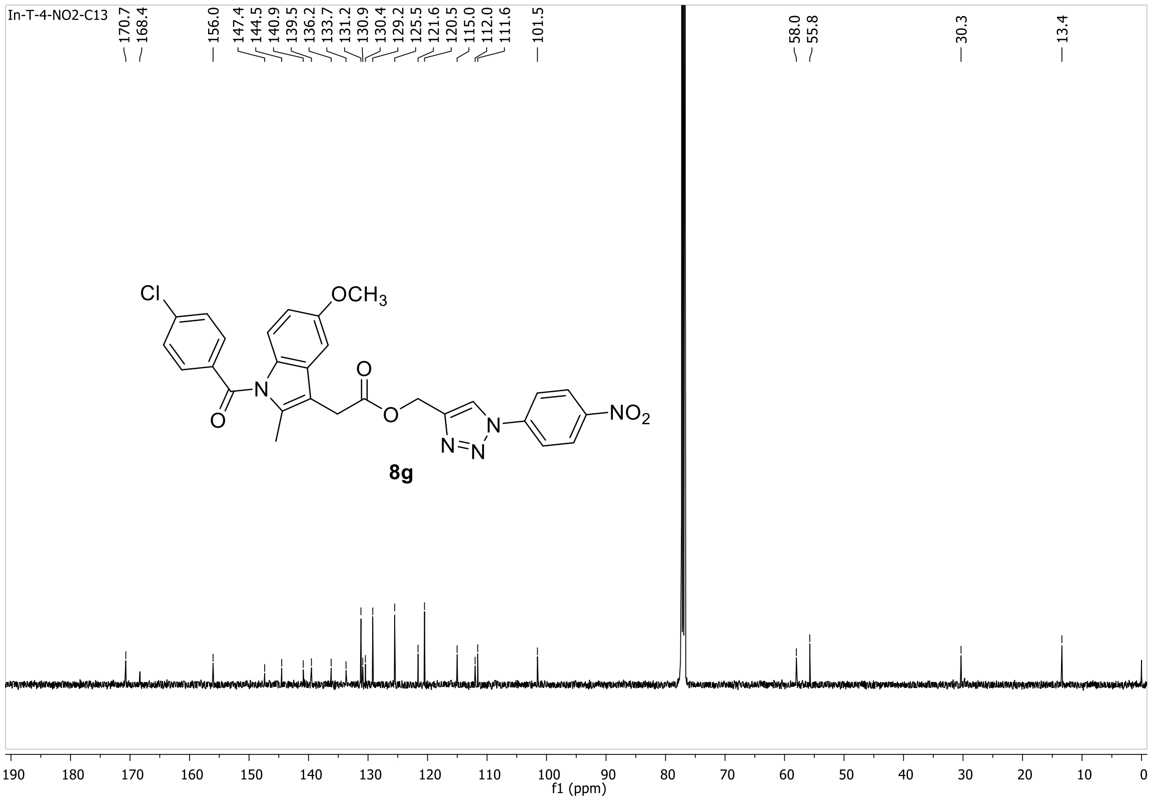
In-T-4-NO2-C13
170.7
168.4
156.0
147.4
144.5
140.9
139.5
136.2
133.7
131.2
130.9
130.4
129.2
125.5
121.6
120.5
115.0
112.0
111.6
101.5
58.0
55.8
30.3
13.4
Cl
OCH3
O
N
O
O
N
N=N
NO2
8g
190 180 170 160 150 140 130 120 110 100 90 80 70 60 50 40 30 20 10 0
f1 (ppm)

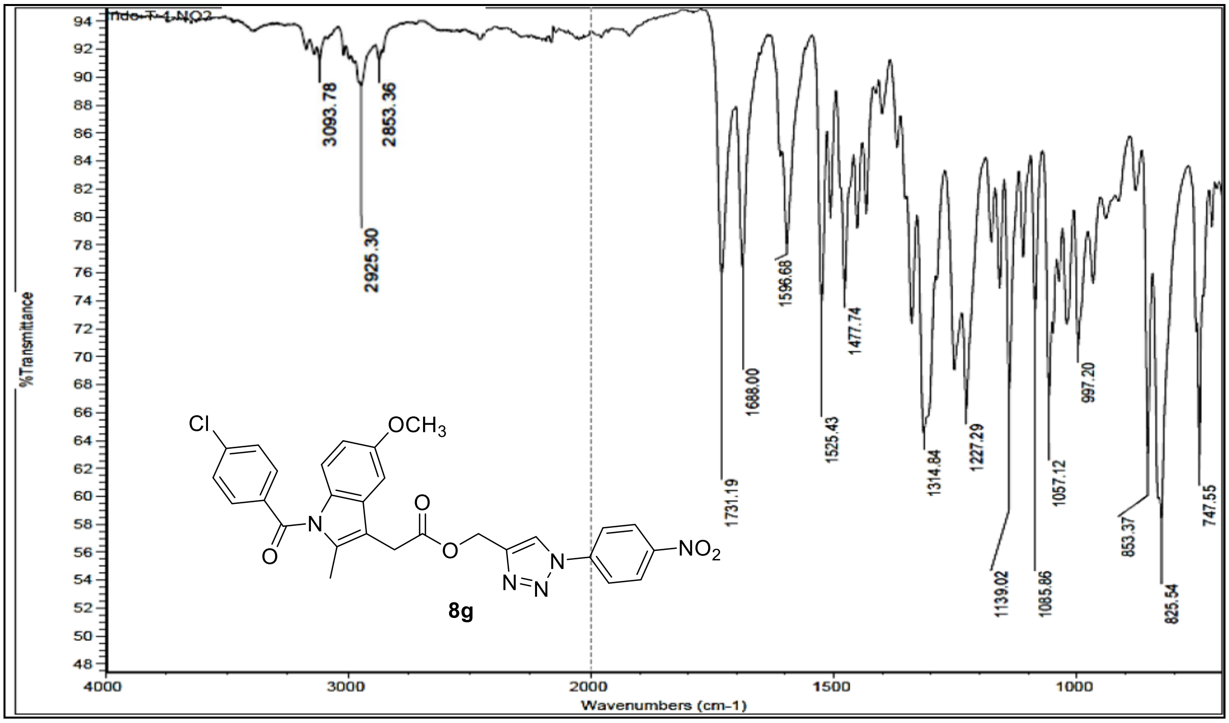
Indo-T-1-NO2
%Transmittance
Wavenumbers (cm-1)
3093.78
2925.30
2853.36
1731.19
1688.00
1596.68
1525.43
1477.74
1314.84
1227.29
1139.02
1085.86
1057.12
997.20
853.37
825.54
747.55
Cl
OCH3
N
O
O
O
N
N=N
NO2
8g
